# MetaGeneBank: a standardized database to study deep sequenced metagenomic data from human fecal specimen

**DOI:** 10.1186/s12866-021-02321-z

**Published:** 2021-09-30

**Authors:** Li Shao, Jie Liao, Jingyang Qian, Wenbin Chen, Xiaohui Fan

**Affiliations:** 1 Hangzhou Normal University, Institute of Translational Medicine, The Affiliated Hospital of Hangzhou Normal University, Hangzhou, 311121 Zhejiang China; 2 Pharmaceutical Informatics Institute, College of Pharmaceutical Sciences, Zhejiang University , Hangzhou, 310003 China; 3 The First Affiliated Hospital, School of Medicine, Zhejiang University , Hangzhou, 310003 Zhejiang China; 4 iMedicine Lab, Alibaba-Zhejiang University Joint Research Center for Future Digital Health , Hangzhou, 310018 Zhejiang China; 5 Westlake Laboratory of Life Sciences and Biomedicine, Hangzhou , Hangzhou, 310058 China

**Keywords:** Gut microbiome, Deep sequenced metagenomes, Human disease, Database

## Abstract

**Background:**

Microbiome big data from population-scale cohorts holds the key to unleash the power of microbiomes to overcome critical challenges in disease control, treatment and precision medicine. However, variations introduced during data generation and processing limit the comparisons among independent studies in respect of interpretability. Although multiple databases have been constructed as platforms for data reuse, they are of limited value since only raw sequencing files are considered.

**Description:**

Here, we present MetaGeneBank, a standardized database that provides details on sample collection and sequencing, and abundances of genes, microbiota and molecular functions for 4470 raw sequencing files (over 12 TB) collected from 16 studies covering over 10 types of diseases and 14 countries using a unified data-processing pipeline. The incorporation of tools that enable browsing and searching with descriptive attributes, gene sequences, microbiota and functions makes the database user-friendly. We found that the source of specimen contributes more than sequencing centers or platforms to the variations of microbiota. Special attention should be paid when re-analyzing sequencing files from different countries.

**Conclusions:**

Collectively, MetaGeneBank provides a gateway to utilize the untapped potential of gut metagenomic data in helping fighting against human diseases. With the continuous updating of the database in terms of data volume, data types and sample types, MetaGeneBank would undoubtedly be the benchmarking database in the future in respect of data reuse, and would be valuable in translational science.

**Supplementary Information:**

The online version contains supplementary material available at 10.1186/s12866-021-02321-z.

## Background

The microbial cells that colonize the human body, such as mucosal and skin environments, are at least as abundant as our somatic cells [1], and contain far more genes than our human genome. Among them, the gut microbiome has gained particular interest due to its large volume, high diversity and relevance to human health and disease. Thanks to metagenomic approaches that allow to study the structure, function and intercellular interactions of gut microbial communities, we know that changes in the microbiome, the microbial metabolome and their interactions with the immune, endocrine and nervous systems are associated with a wide array of illness, such as inflammatory bowel disease [2–5], obesity [6], cancer [7], type 2 diabetes [8, 9], and major depressive disordes [10, 11]. Nowadays, gut microbiome research is transitioning from a descriptive to a causal and finally to a translational science. Numerous independent studies have found specific microbial fingerprints that may be useful in providing markers for disease diagnosis and prognosis [12, 13], and new ideas for disease intervention and treatment [14–17]. It is also reported that a full spectrum of sources generated from collaborative projects such as Human Microbiome Project (HMP) [18] can generate meaningful interpretations that are impossible with independent studies [19]. Such results indicated that, as a data driven science [20], microbiome big data holds the key to unleash the power of microbiome to overcome critical challenges in disease control, treatment and even precision medicine [21].

Numerous reports have indicated that comparisons among independent studies are limited in respect of interpretability due to the batch effects [22] and known differences associated with data generation and processing procedures [23–25] utilized in independent studies. As a potential solution, multiple databases such as European Bioinformatics Institute (EBI) [26–28], Sequence Read Archive (SRA) [29] in National Center for Biotechnology Information (NCBI) are available to deposit raw metagenomic sequencing files generated in independent studies. gcMeta [19], a global catalogue of metagenomics platform developed for archiving, standardization and analysis of microbiome data, also provides the link address of raw sequencing reads in databases such as EBI [26]. However, carrying out meta-analyses using metagenomic data deposited in above databases is still challenging at present. In metagenomic studies, complex data-generation and bioinformatic processing procedures are required preceding the calculation of compositional profiles and ecological indices. The data-generation process consists of multiple steps including sample collection, DNA extraction, library preparation and sequencing, while the bioinformatic processing process involves quality control, removing host contamination, assembly, gene prediction, taxonomical and functional annotation and so on [30]. Each of the above steps is subject to technical variability [31]. Actually, independent studies have almost exclusively used their own methodology and a demographically distinct cohort. It is therefore necessary to standardize all sequencing files with the same pipeline to prelude the variations introduced by bioinformatic processing protocols, and take a consideration of detailed descriptions on data generation process in independent studies in respect of meta-analyses. However, existing databases store only raw sequencing files. The lack of details on data generation process and the undone laborious tasks of data processing using a unified bioinformatic pipeline weakens the value of them in respect of data reuse. These, in together with the ongoing effort to better standardize and integrate data resources to better understand microbial dynamics in human systems [32], confirm the urgent need of a new curated, standardized and user-friendly database.

Here, we present MetaGeneBank, a standardized database to study deep sequenced metagenomic data from human fecal specimen. The aim of the database is to provide a gateway to utilize the untapped potential of gut metagenomic data in disease control and treatment. To build the database, we collected a total of 4470 deep sequenced metagenomic sequencing files from human fecal specimen and corresponding details on data generation process provided in independent studies published prior to September 2018. More than 10 types of human diseases have been covered in current version of MetaGeneBank. In response to the calling to make all scientific data ‘findable, accessible, interoperable and reusable’ (FAIR) [33], all sequencing files are scaled by a unified bioinformatic processing pipeline and a unified non-redundant gene list covering genes from all sequencing files. The resulting multi-level processed data such as abundance tables for genes, microbiota and molecular functions in KEGG orthology (KO) as well as raw sequencing files, are available via a web interface (http://tcm.zju.edu.cn/mgb) for free search and download. It should be noted that all the processed data in the database are comparable and reusable. The tight connections with tools that allows user-friendly data search and export, data illustration using PCA (principal component analysis) makes it a powerful resource for metagenomic data integration and reuse.

## Construction and content

### Design of MetaGeneBank database

**Fig. 1:**
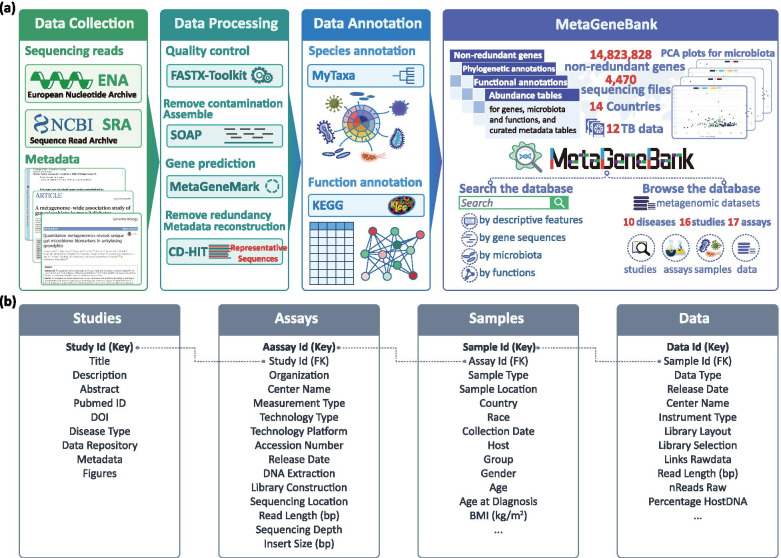
Overview of MetaGeneBank database. **a** General workflow for acquiring, processing and achiving metagenomic data. The workflow consists of four processes. The data acquisiton of raw sequencing files (FASTQ) and the study metadata, followed by data processing and annotation procedures. All outputs are integrated into the MetaGeneBank database. Users can browse, search and download the datasets, annotation results and corresponding figures. **b** Database scheme of MetaGeneBank. Main data structure and relationships between the different tables are illustrated

The methodology of the data production for MetaGeneBank is illustrated in Fig. 1. Figure 1(a) shows the method used for collecting, processing, annotating the deep sequenced metagenomic sequencing files, and the information and tools provided in MetaGeneBank. Figure 1(b) illustrates the scheme of the database. The major data record types are ‘Studies’, ‘Assays’, ‘Samples’ and ‘Data’. ‘Studies’ summarizes the details of each study, such as article title, article abstract, data repository, and disease type. ‘Assays’ illustrates the information of assays that have been carried out for each study, including measurement type, technology type, technology platform, release date and so on. ‘Samples’ demonstrates the clinical indices of samples used for each assay in each study. ‘Data’ hosts the details of each data, such as the links of raw sequencing data and the summary statistics of bioinformatic processing process for each sequencing file. To date, MetaGeneBank has archived a total of 4470 deep sequenced metagenomic sequencing files in gzip format (more than 12 TB) collected from 17 assays of 16 studies carried out in over 14 different countries to associate human fecal microbiota with more than 10 types of diseases [5, 30, 34–45]. Detailed information about the data source is shown in Table 1.

**Table 1:** Detailed information about the data source collected in the database

Assay	Accession number	Number of sequencing files	Number of paired files	Number of single files
AS1.as1	SRP100575	211	211	
ACD1.as1	ERP023788	385	385	
CFS1.as1	SRP102150	100	41	59
CC1.as1	ERP008729	310	154	156
IBD1.as1, IBD2.as1, IBD3.as1, IBD4.as1	PRJNA389280; ERA000116; PRJEB15371;ERP002061	1476	1476	
LC1.as1	ERP005860	237	237	
Obesity1.as1	ERP003612	595	595	
RA1.as1	PRJEB6997	137	137	
T1D1.as1	PRJNA231909	126	126	
T2D1.as1, T2D1.as2, T2D2.as1, T2D3.as1	SRA050230; SRA045646; ERP004605; ERP02469	807	802	5
NAFLD1.as1	PRJNA373901	86	86	

### Data acquisition, metadata reconstruction

All the deep sequenced metagenomic datasets were collected by searching PubMed for human gut metagenomic articles. Considering that the bioinformatic processing workflow is time-consuming, only references before September 2018 were considered in this study. After removing articles in which raw sequencing files or grouping information were not available, we retrieved study accession numbers and metadata from the retained articles. The study accession numbers were used to retrieve sequencing read files deposited in databases such as EBI. For samples which have both pair-end and single-end sequences, only pair-end sequence files were involved in the database. Each raw sequence file consists of one or two FASTQ files depending on its library construction method. The study metadata contains basic information about the biological samples that authors provided. The downloaded study metadata was further curated and reformatted in txt files based on the information provided in ‘Sample’ as shown in Fig. 1(b). Table S1 illustrated the distribution of samples per disease and country.

### Data processing

The primary data processing protocol can be separated into two sections. Firstly, procedures including quality control, screening and removing of human contaminations, assembly, assembly revision, and gene prediction, are carried out for each sequencing file. For read quality trimming and filtering, FastX program (http://hannonlab.cshl.edu/fastx_toolkit/) was used to remove raw reads below quality cutoff 20 and length cutoff 45 bp. High quality reads were then mapped to the human reference genome (assembly GRCh38.p11) to remove reads of human origin using SOAPaligner v2.21 with parameters ‘-M 4, -l 30, -r 1, -v 10’ [46]. Reads that passed human contamination screening were assembled into scaftigs using SOAPdenovo v1.06 with default parameters [47]. Kmer size was estimated at run-time for each individual metagenome as the closest odd number greater than half the average read length. In the following assembly revision procedure, base errors, short indels and chimeric contigs were further corrected, and scaftigs with length less than 500 bp were removed. Protein coding genes on the metagenomes were predicted from the sequences of scaftigs using MetaGeneMark v3.38 [48, 49]. It took around 4 h to finish the standardizing process including quality control, filtering, assemble, assemble revision, and gene prediction for a single dataset (about 4 gigabytes) on a high-performance server (Intel Xeon CPU E5-4620, memory 754G, CentOS 6). In the second section, genes predicted from each sequencing file were pooled together and merged with the first integrated gene catalog (IGC, including type 2 diabetes [T2D], IBD, human microbiome project [HMP] and MetaHIT individuals) containing 9879, 896 genes from 1267 gut metagenomes [50]. The final non-redundant gene list containing 14,823,828 genes was obtained using CD-HIT v4.6 [51] with parameters of sequence identity threshold 0.95 and 90% alignment coverage for the shorter sequence. High quality reads that passed human contamination screening were then mapped to the final non-redundant genes using SOAPaligner v2.21 with parameters ‘-M 4 -l 30 -r 1 -v 5’. After filtering with length cutoff 30 bp and 95% identity, the mapped reads were utilized to calculate gene length-normalized base counts using soap coverage script (http://soap.genomics.org.cn/down/soap.coverage.tar.gz). Eleven M high quality reads were randomly drawn without replacement for each sequencing file that passed quality control and human contamination removal procedures to avoid the bias caused by variations in sequencing depth. Then, the retrieved reads were mapped to the final non-redundant genes to form a downsized depth or abundance [40, 45]. The abundance table was then normalized to ensure that the total relative abundances for all genes in each sample was 100.

### Taxonomical and functional annotation

Genes in the final non-redundant gene list were assigned taxonomical annotations using MyTaxa [52], a homology-based bioinformatics framework to classify metagenomic sequences with unprecedented accuracy, based on the sequence similarity of each gene to a database of predicted protein coding genes from 8942 publicly available genomes in the National Center for Biotechnology Information (NCBI, release 196). The distinguishing aspect of MyTaxa is that it provides a likelihood score that measures the possibility that the query sequence originated from that taxon. If the top-scoring taxon at a given rank passes the likelihood score cutoff, MyTaxa predicts the query sequence to belong to this specific taxon. A likelihood cutoff 0.5 was utilized to determine the taxonomical annotation for the query sequence. Functional annotation for target genes was achieved by aligning the sequences of the genes in the non-redundant gene list to KEGG Orthology database (KO) using DIAMOND, which is a BLAST-compatible local aligner but about 20,000 times faster on short reads. A sensitive alignment mode with a 16 × 9 seed shape configuration and an maximum expected value of 0.00001 was used to keep an alignment. The relative abundances of annotated microbiome and KO functions were calculated as the total relative abundance of genes annotated to it.

### Secondary data analysis

PCA was conducted on the abundance tables of annotated microbiota using the prcomp function in R version 3.5.1 (https://www.r-project.org/).

### System architecture of MetaGeneBank

The MetaGeneBank platform is implemented using nodejs and python language. Some scientific function is powered by python using scikit-learn/pandas/scipy/numpy. The nodejs is used as middleware api. The web server of MetaGeneBank is nginx.

## Utility and discussion

### Web-interface to the MetaGeneBank database

The MetaGeneBank interface (http://tcm.zju.edu.cn/mgb) is designed to enable users to navigate through and perform basic operations, such as browsing, searching and downloading data. No login is required for any users, and all the multi-level processed data are ready for download. The user-interface is divided into the following sub-pages.

#### The ‘Home’ page

This page gives a general description about the data sources, brief usage and key features of the database.

#### The ‘Datasets’ page

This page deposits all the data sources provided in the database, and can be divided into five sub-views including ‘Metagenomic Studies’, ‘Metagenomic Assays’, ‘Metagenomic Samples’, ‘Metagenomic Data’ and ‘Supplemental Materials’.

**Fig. 2:**
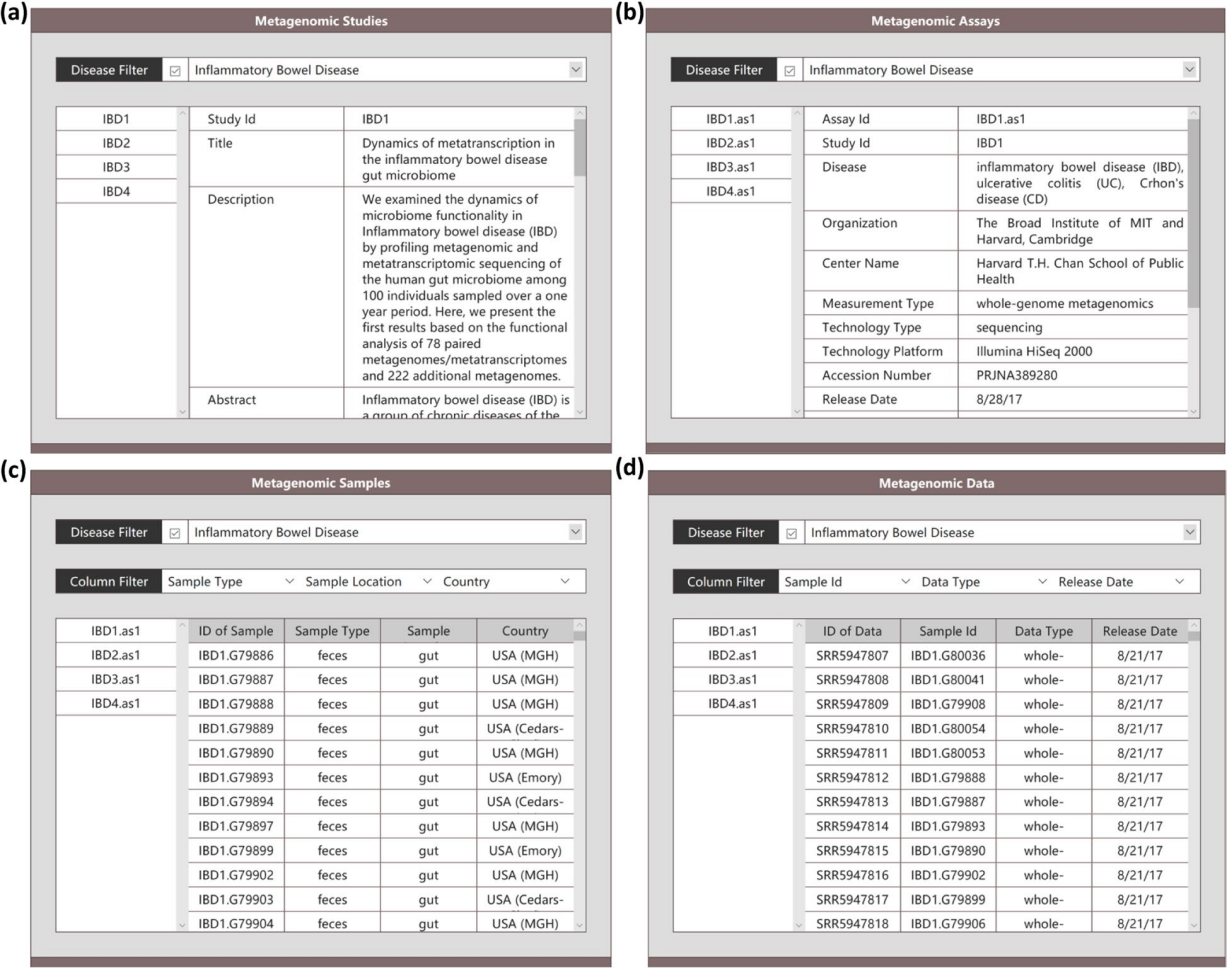
The ‘Dataset’ page in MetaGeneBank database. An illustration of **a** ‘*Metagenomic Studies*’, **b** ‘*Metagenomic Assays*’, **c** ‘*Metagenomic Samples’*, **d** ‘*Metagenomic Data*’ views, respectively

1. *‘Metagenomic Studies’ view*: this view gives a general description of each metagenomic study in the database (Fig. 2a). It provides information such as the title of the main article publication, the dataset summary which illustrates the aim of the dataset and how it has been generated, the abstract of the main article publication, the link to the PubMed record and DOI (digital object identifier) of the published article, the type of disease surveyed in the study, the link to the public data repository, the metadata provided by authors before curation for downloading, and the PCA plots for all samples in each study in terms of annotated microbiota. Users are able to use ‘Disease Filter’ to access detailed information of target studies from this view directly. For a disease type with multiple studies, all related studies are displayed in the left most column after filtering. Users can get detailed information about each study after clicking them.
2. ‘*Metagenomic assays’ view*: This view shows the detailed information of data-generation process for each assay carried out in each study, such as measurement type, organization, center name, technology type, technology platform, release date, accession number and so on (Fig. 2b). Users can search for target assays by filtering diseases using ‘Disease Filter’. Some studies can have more than one assays. In this case, all related assays are displayed in the left most column after filtering. Users can get detailed information about each assay aftering clicking them.
3. ‘*Metagenomic samples’ view*: As shown in Fig. 2c, this view illustrates the metadata of biological samples utilized in each assay, including sample id, assay id, sample location, country, group, gender, age, BMI (body mass index), ALT (alanine transaminase), AST (aspartate transaminase), ALB (albumin), creatinine, triglyceride, etc. Users can browse samples in this view by restricting diseases using ‘Disease Filter’ and selecting interested columns using ‘Column Filter’ since the complete information is too large to be displayed in a page.
4. ‘*Metagenomic data’ view*: This view (Fig. 2d) demonstrates the links of raw sequencing files, and statistics of data processing pipeline for each sequencing file such as read length, the number of raw reads (‘nReads Raw’), the number of clean reads (‘nReads Clean’), the number of contigs (‘nContigs’), N50 length (‘N50 Length’), the number of open reading frames (‘nORFs’) and so on. Users can filter for data by restricting diseases using ‘Disease Filter’ in this view. After selecting a disease type, all related assays will be illustrated in the left most column. Users can obtain detailed information about each assay after clicking it in the left most column. Since the table is too large to be displayed in a page, ‘Column Filter’ is also provided to allow users to select and display interested information.
5. *‘Supplemental materials’*: This view provides the sequences, the taxonomical and functional annotation results of the unified non-redundant genes. The references for microbiota (phylum, class, order, family, genus, species) and KO functions (hierarchies A to D) used for searching are also provided. Users can obtain details about the possible search terms from here.

#### The ‘Search’ page

**Fig. 3:**
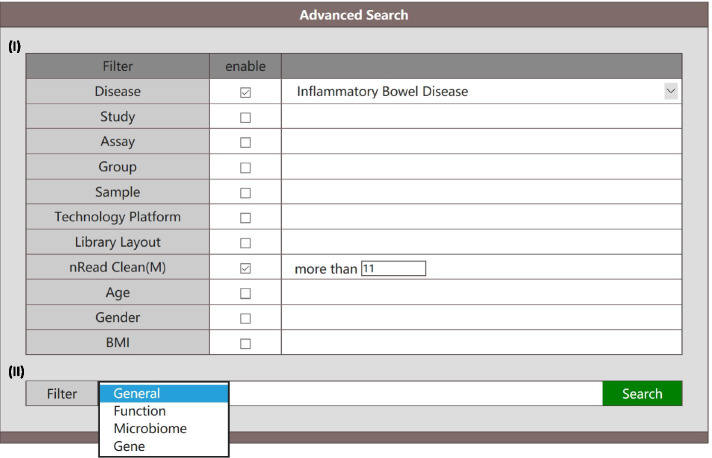
The ‘Search’ page in MetaGeneBank database. An illustration of descriptive attributes (I) and search modes (II)

**Fig. 4:**
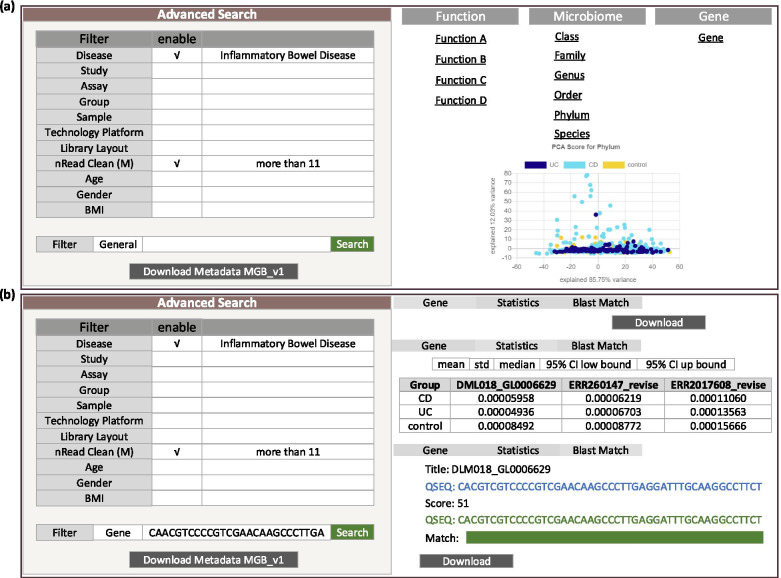
An illustration of ‘General’ (**a**) and ‘Gene’ (**b**) search modes and corresponding outputs

Two steps of filtering shown in sections I and II of Fig. 3 are provided in this page to allow users to search for target data. As the first step, parameters provided in section I allow users to search by general descriptive attributes, including ‘Disease’, ‘Study’, ‘Assay’, ‘Technology Platform’, ‘Library Layout’, ‘nReads Clean (M)’, ‘Age’, ‘Gender’, and ‘BMI’. All above parameters can be restricted at the same time if needed. If left blank, the parameters will not be restricted in this search. The minimal information required for search is ‘Disease’ and ‘nReads Clean (M)’ (default 11 M). The candidate values for each parameter that can be used in searching is shown in Table S2. In section II, four search modes (General, Gene, Microbiome, Function) are provided to allow users to search by general descriptive information, genes, microbiota and functions of interest, respectively. When ‘General’ is selected (Fig. 4a), no further information is required. The output includes metadata meeting the searching requirements provided in section I, and a series of abundance tables for genes, microbiota (6 levels) and functions (4 hierarchies). All tables can be downloaded directly. Moreover, a PCA plot for resuting phylum is also illustrated. When ‘Gene’ is selected (Fig. 4b), users should also provide the sequences of target genes except those provided in section I. Other than the associated metadata (marked by ‘Download Metadata MGB_V1’), the searching output includes the alignment information of top 5 matches (marked by ‘Blast Match’) and the abundance table of them in target populations (marked by ‘Gene’), which can be downloaded directly. A summary table containing statistics of target genes (marked by ‘Statistics’) including the mean, standard deviation, median and 95% confidence intervals (CI) is also illustrated. When ‘Microbiome’ is selected (Figure S1a), users must select a target level (such as Class) and fill in the names of target microbiota, other than the requirements provided in section I. After searching, the associated metadata (marked by ‘Download Metadata MGB_V1’) and abundance table of target microbiota in target populations (marked by ‘Phylum’, ‘Class’, ‘Order’, ‘Family’, ‘Genus’ or ‘Species’ according to the level of input microbiota) are generated for downloading. The summary table (marked by ‘Statistics’) containing mean, standard deviation, median and 95% CIs of target microbiota is also demonstrated. When ‘Function’ is selected (Figure S1b), users must also choose a target hierarchy (A, B, C, D) and fill in the names of target functions besides those requirements provided in section I. Here, the functions of genes in MetaGeneBank were annotated using KEGG orthology (KO), a database of molecular functions represented in terms of functional orthologs. Functional orthologs are organized in several hierarchies (such as A, B, C, D), which represent different levels of functional annotations for each KO in KEGG database (Figure S2). Moreover, we have provided the document associating KO annotations with GO Terms (Table S3) in the revised manuscript for users who need the functional information based on GO Terms. Based on the association file, users can convert the target GO Terms to KOs, and retrieve corresponding abundance table and statistic information for target GO terms from the website using KO annotations. The searching results will be the associated metadata (marked by ‘Download Metadata MGB_V1’) and abundance table of target functions in target populations (marked by ‘FunctionA’, ‘FunctionB’, ‘FunctionC’ or ‘FunctionD’ according to the hierarchy of input functions) for downloading, and the summary table (marked by ‘Statistics’) including mean, standard deviation, median and 90% CI for target functions demonstrated in the page. Multiple inputs are supported in ‘Microbiome’ and ‘Function’ modes in section II. By using the abundance table for queried features (such as ‘abundance.Microbiome.Class.MGB_V1.csv’), users can also evaluate the difference among groups using Wilcoxon rank-sum test or Kruskal-wallis test.

#### The ‘User guide’ and ‘About’ pages

The ‘User guide’ page illustrates the details on how to browse metagenomic studies, metagenomic assays, metagenomic samples and metagenomic data provided in the database. Taking inflammatory bowel disease (IBD) as an example, the ‘User guide’ page also gives a detailed description on how to search for target data based on general descriptive information alone using ‘General’ mode, and for interested genes, microbiota and functions based on sequences of targe gene, names of target microbiota and functions together with general descriptive information using ‘Gene’, ‘Microbiome’ and ‘Function’ modes, respectively. The ‘About’ page provides details about the data sources provided in the database and the contact information.

### Application scenario

**Fig. 5:**
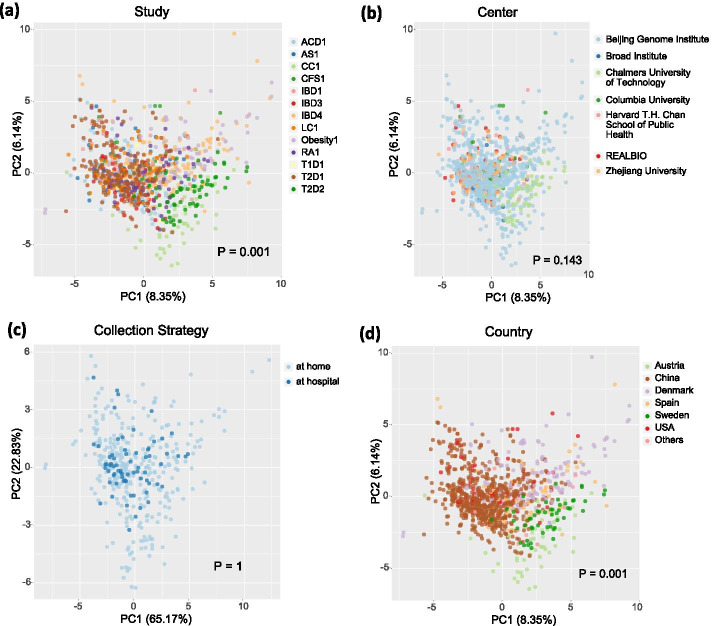
The PCA score plots for all healthy controls based on the microbial abundance in phylum level. The solid circles in (**a**-**d**) are colored according to studies, sequencing centers, sample collection strategies, and sample collection countries, respectively

Based on the huge amount of data that have been scaled with the unified bioinformatic processing pipeline and the unified nonredundant genes covering all sequencing files in the database, we retrieved the abundance table of microbiota in phylum level from all healthy controls (1056), and evaluated the variations among independent studies. It is revealed by the score plot of PCA that apparent differences exist among independent studies (Fig. 5a), which might be explained by the variations introduced during sample collection, data-generation and data-processing procedures. Since the variations due to data processing protocols have been precluded by using a unified bioinformatic processing pipeline, the above results indicate that variations due to sample collection and/or data-generation procedures cannot be neglected in respect of meta-analysis. Considering that protocols for data generation such as DNA extraction, library preparation and sequencing might be different in each center, we then assigned the data to corresponding centers. As shown in Fig. 5b, variations among centers is far less apparent than those among studies, implying that variations introduced by data-generation process may not be the most important reason to the differences among studies. It is therefore not surprising to see that no evident differences exist among data generated using different sequencing platforms (Figure S3a). Finally, we evaluated the impact of sample collection process. As shown in Fig. 5c, the impact of sample collection strategy (at home or at hospital) is not apparent, while obvious difference is observed for data generated from samples collected from different countries (Fig. 5d). Moreover, the pattern of variation in Fig. 5d is similar to that in Fig. 5a, implying that the source of samples is the most important reason to the variations among different studies. As a confirmation, we further selected a subset of data (461) sequenced by HiSeq 2000 in Beijing Genome Institute (BGI) to preclude the effect of sequencing centers and platforms. Figure S3b confirms the apparent variations among independent assays and among specimen collected from different countries (Figure S3d), while the difference related to sampling strategies is not so apparent (Figure S3c). Such results indicate that variations caused by source of specimen are more significant than those by data generation procedures such as sequencing centers and platforms. Special attention should be paid when re-analyzing sequencing files generated from specimen collected in different countries.

Microbiome analysis is now revolutionizing clinical investigations by providing greater patient stratification and novel biomarkers of disease [53]. However, it is a brave new world, one where there is still a great gap between microbial analysis and microbiome-informed or microbiome-based medicine. Integrated efforts from ecologists, data scientists and clinical researchers are therefore urgently needed to realize the full potential of microbiome science. MetaGeneBank, the first standardized database that provides details on data-generation process and scaled multi-level processed metagenomic data from independent studies associating gut microbiome with human diseases, is destined to be a valuable platform for collaborations from scientists who are dedicated to move closer to the realization of more effective diagnosis, treatment and prevention strategies against human diseases.

Till now, a huge amount of whole-genome metagenomic and 16S sequencing data have been generated. Multiple databases such as EBI [26–28], SRA [29] and gcMeta [19] have been constructed to deposit the raw 16S and whole-genome metagenomic sequencing files. Considering the challenges including analyzing Gigabyte (GB) to Terabyte (TB) scale data on a single computer, gcMeta also provided online tools for processing 16S sequencing data, which made it possible for the meta-analyses of 16S sequencing data. However, no such databases are available for reuse of whole-genome metagenomic sequencing data, partially due to the fact that whole-genome metagenomic data are storage space intensive and the data analysis pipeline such as the assembly procedure is very computationally expensive. Moreover, whole-genome metagenomic sequencing data can provide more detailed information about gut microbiota such as functions compared to 16S sequencing data. Therefore, we selected whole-genome metagenomic sequencing data as the first batch of sequencing files to be collected and standardized in current version of MetaGeneBank.

Compared to databases such as EBI which deposit only raw sequencing reads, a key feature of the database is that all sequencing files in the database are processed with a unified bioinformatic processing pipeline, and the resulting multi-level processed data, such as abundance tables for genes, annotated microbiota and KO functions, are readily reusable and comparable. Another key feature of the database is that details on metadata of biological samples and data-generation procedures are also hosted. The tight connections with tools that allows user-friendly data brose, search and export makes it a powerful resource for metagenomic data integration and reuse. Base on the database, we found that the variations of microbiota caused by source of specimen are more significant than those by sequencing centers or platforms. Such results indicated that special attention must be paid to sequencing files from fecal specimen collected from different countries in case of data reuse.

## Conclusions

Although there is still a long way to go before the value of microbiome can be fully realized [32], with MetaGeneBank keep providing standardized data that are needed to test the hypotheses, more and more gaps will be possible to be closesd in future studies when the database is huge enough. Over the next few years, we will keep updating the database as soon as seminal studies are published and following requests from users. We plan to incorporate metagenomic data from other body parts of human, such as mouth, stomach, blood, urine and so on, and other types of data from 16S sequencing and metatranscriptomics assays in future update of the database. Moreover, the database design of MetaGeneBank will be scale-up to meet the increasing demand of microbiome research. With the continuous updating of the database in terms of data volume, data types and sample types, we believe that MetaGeneBank will be an valuable resource for the metagenomics research community in respect of meta-analysis.

## Supplementary Information

**Additional file 1 **: **Figure S1**. An illustration of ‘Microbiome’ (a) and ‘Function’ (b) search modes and corresponding outputs.

**Additional file 2 **: **Figure S2**. An example of KO hierarchies A, B, C, and D.

**Additional file 3 **: **Figure S3**. The PCA score plots for microbial abundance in phylum level. (a) The score plot for microbial abundance of all healthy controls colored according to sequencing platforms. (b-d) The score plot for microbial abundance of healthy controls sequenced in BGI center and with HiSeq 2000 platform. The solid circles in (b-d) are colored according to assays, sample collection strategies, and sample collection countries, respectively.

**Additional file 4 **: **Table S1**. The distribution of samples per disease and country.

**Additional file 5 **: **Table S2**. The candidate values for each parameter that can be used in searching.

**Additional file 6 **: **Table S3**. A document associating KO annotations with GO Terms.

## Data Availability

The datasets supporting the conclusions of this article are available in the web interface (http://tcm.zju.edu.cn/mgb).

## Abbreviations

TB: Terabyte
GB: Gigabyte
HMP: Human Microbiome Project
EBI: European Bioinformatics Institute
SRA: Sequence Read Archive
NCBI: National Center for Biotechnology Information
gcMeta: A global catalogue of metagenomics platform
FAIR: ‘Findable, accessible, interoperable and reusable’
KO: KEGG orthology
KEGG: Kyoto Encyclopedia of Genes and Genomes
PCA: Principal component analysis
BGI: Beijing Genome Institute
DOI: Digital object identifier
BMI: Body mass index
ALT: Alanine transaminase
AST: Aspartate transaminase
ALB: Albumin
CI: Confidence intervals
IBD: Inflammatory bowel disease
IGC: Integrated gene catalog
T2D: Type 2 diabetes
MetaHIT: Metagenomics of the Human Intestinal Tract
