# Supplementary material for: MetaGeneBank: a standardized database to study deep sequenced metagenomic data from human fecal specimen
Source: BMC Microbiol. 2021 Sep 30;21:263. doi: 10.1186/s12866-021-02321-z (PMC8485520; doi:10.1186/s12866-021-02321-z)
Supplement: Supplementary file 1 — Additional file 1 : Figure S1. An illustration of ‘Microbiome’ (a) and ‘Function’ (b) search modes and corresponding outputs. [file 12866_2021_2321_MOESM1_ESM.pdf]

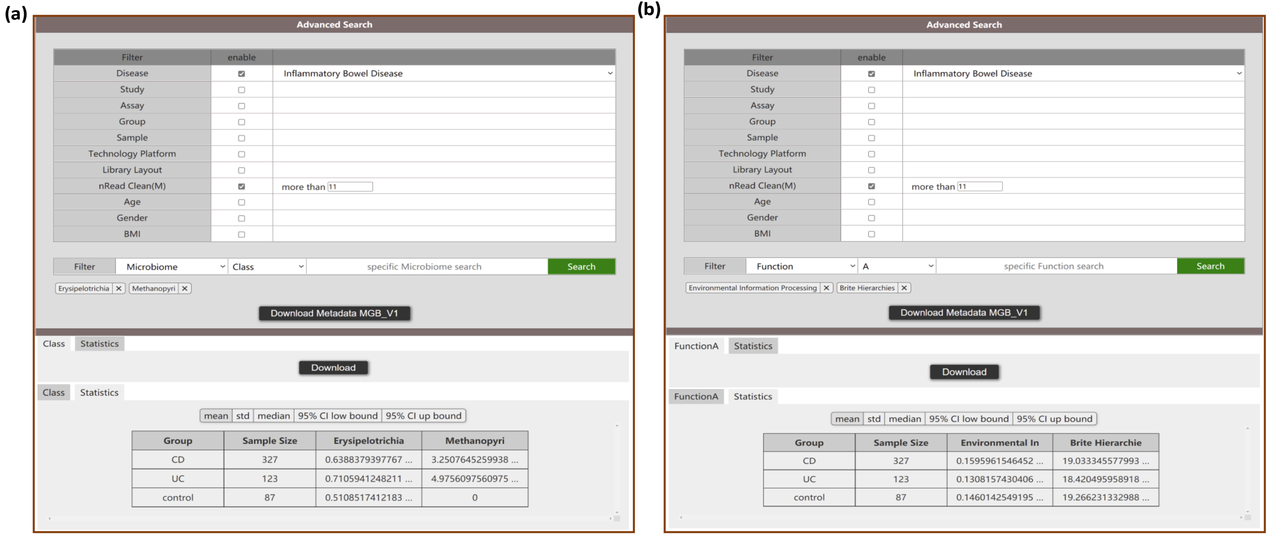
(a)
Advanced Search
Filter
enable
Disease
Inflammatory Bowel Disease
Study
Assay
Group
Sample
Technology Platform
Library Layout
nRead Clean(M)
more than 11
Age
Gender
BMI
Filter
Microbiome
Class
specific Microbiome search
Search
Erysipelotrichia
Methanopyri
Download Metadata MGB_V1
Class
Statistics
Download
Class
Statistics
mean
std
median
95% CI low bound
95% CI up bound
Group
Sample Size
Erysipelotrichia
Methanopyri
CD
327
0.6388379397767 ...
3.2507645259938 ...
UC
123
0.7105941248211 ...
4.9756097560975 ...
control
87
0.5108517412183 ...
0
(b)
Advanced Search
Filter
enable
Disease
Inflammatory Bowel Disease
Study
Assay
Group
Sample
Technology Platform
Library Layout
nRead Clean(M)
more than 11
Age
Gender
BMI
Filter
Function
A
specific Function search
Search
Environmental Information Processing
Brite Hierarchies
Download Metadata MGB_V1
FunctionA
Statistics
Download
FunctionA
Statistics
mean
std
median
95% CI low bound
95% CI up bound
Group
Sample Size
Environmental In
Brite Hierarchie
CD
327
0.1595961546452 ...
19.033345577993 ...
UC
123
0.1308157430406 ...
18.420495958918 ...
control
87
0.1460142549195 ...
19.266231332988 ...
