# Supplementary material for: MetaGeneBank: a standardized database to study deep sequenced metagenomic data from human fecal specimen
Source: BMC Microbiol. 2021 Sep 30;21:263. doi: 10.1186/s12866-021-02321-z (PMC8485520; doi:10.1186/s12866-021-02321-z)
Supplement: Supplementary file 2 — Additional file 2 : Figure S2. An example of KO hierarchies A, B, C, and D. [file 12866_2021_2321_MOESM2_ESM.pdf]

A ▼ 09100 Metabolism

B ▼ 09101 Carbohydrate metabolism

C ▼ 00010 Glycolysis / Gluconeogenesis [PATH:ko00010]

D K00844 HK; hexokinase [EC:2.7.1.1]
K12407 GCK; glucokinase [EC:2.7.1.2]
K00845 glk; glucokinase [EC:2.7.1.2]
K25026 glk; glucokinase [EC:2.7.1.2]
K01810 GPI, pgi; glucose-6-phosphate isomerase [EC:5.3.1.9]
K06859 pgi1; glucose-6-phosphate isomerase, archaeal [EC:5.3.1.9]
K13810 tal-pgi; transaldolase / glucose-6-phosphate isomerase [EC:2.2.1.2 5.3.1.9]
K15916 pgi-pmi; glucose/mannose-6-phosphate isomerase [EC:5.3.1.9 5.3.1.8]
K24182 PFK9; 6-phosphofructokinase [EC:2.7.1.11]
K00850 pfkA, PFK; 6-phosphofructokinase 1 [EC:2.7.1.11]
K16370 pfkB; 6-phosphofructokinase 2 [EC:2.7.1.11]
K21071 pfk, pfp; ATP-dependent phosphofructokinase / diphosphate-dependent phosphofructokinase [EC:2.7.1.11 2.7.1.90]
K00918 pfkC; ADP-dependent phosphofructokinase/glucokinase [EC:2.7.1.146 2.7.1.147]
K00895 pfp, PFP; diphosphate-dependent phosphofructokinase [EC:2.7.1.90]
K03841 FBP, fbp; fructose-1,6-bisphosphatase I [EC:3.1.3.11]
K02446 glpX; fructose-1,6-bisphosphatase II [EC:3.1.3.11]
K11532 glpX-SEBP; fructose-1,6-bisphosphatase II / sedoheptulose-1,7-bisphosphatase [EC:3.1.3.11 3.1.3.37]
K01086 fbp-SEBP; fructose-1,6-bisphosphatase I / sedoheptulose-1,7-bisphosphatase [EC:3.1.3.11 3.1.3.37]
K04041 fbp3; fructose-1,6-bisphosphatase III [EC:3.1.3.11]
K01623 ALDO; fructose-bisphosphate aldolase, class I [EC:4.1.2.13]
K11645 fbaB; fructose-bisphosphate aldolase, class I [EC:4.1.2.13]
K01624 FBA, fbaA; fructose-bisphosphate aldolase, class II [EC:4.1.2.13]
K01622 K01622; fructose 1,6-bisphosphate aldolase/phosphatase [EC:4.1.2.13 3.1.3.11]
K16305 K16305; fructose-bisphosphate aldolase / 6-deoxy-5-ketofructose 1-phosphate synthase [EC:4.1.2.13 2.2.1.11]
K16306 K16306; fructose-bisphosphate aldolase / 2-amino-3,7-dideoxy-D-threo-hept-6-ulosonate synthase [EC:4.1.2.13 2.2.1.10]
K01803 TPI, tpiA; triosephosphate isomerase (TIM) [EC:5.3.1.1]
K00134 GAPDH, gapA; glyceraldehyde 3-phosphate dehydrogenase [EC:1.2.1.12]
K10705 GAPDHS; glyceraldehyde-3-phosphate dehydrogenase, spermatogenic [EC:1.2.1.12]
K00150 gap2; glyceraldehyde-3-phosphate dehydrogenase (NAD(P)) [EC:1.2.1.59]
K00927 PGK, pgk; phosphoglycerate kinase [EC:2.7.2.3]
