# Supplementary figures and images for: MetaGeneBank: a standardized database to study deep sequenced metagenomic data from human fecal specimen
Source: BMC Microbiol. 2021 Sep 30;21:263. doi: 10.1186/s12866-021-02321-z (PMC8485520; doi:10.1186/s12866-021-02321-z)

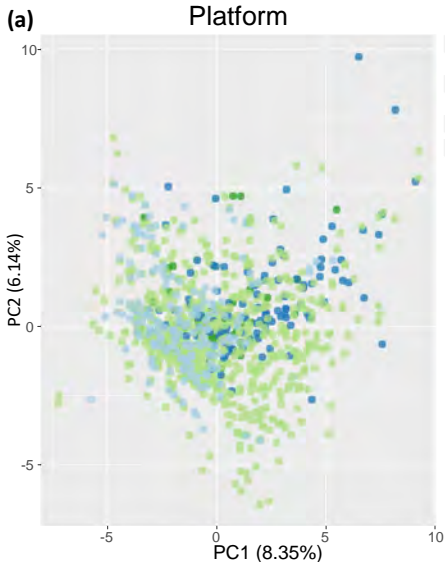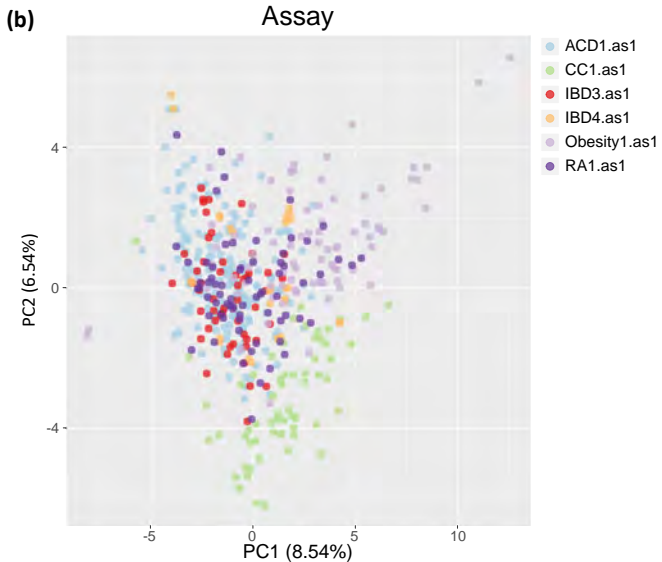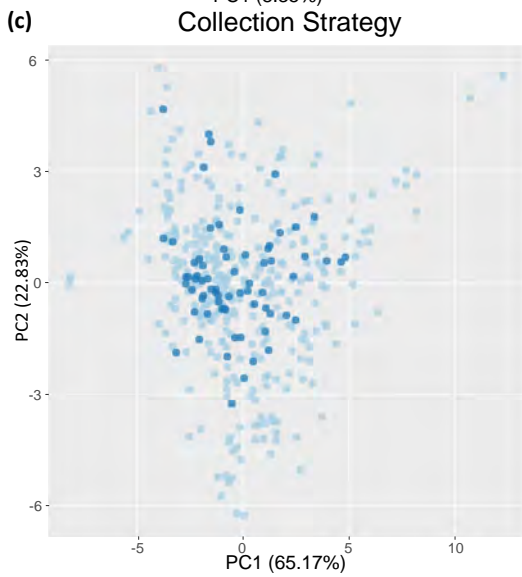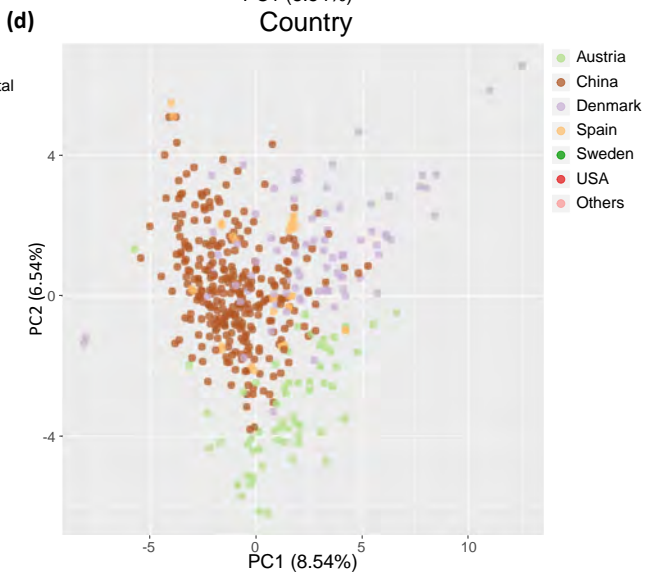

Supplement: Supplementary file 3 — Additional file 3 : Figure S3. The PCA score plots for microbial abundance in phylum level. (a) The score plot for microbial abundance of all healthy controls colored according to sequencing platforms. (b-d) The score plot for microbial abundance of healthy controls sequenced in BGI center and with HiSeq 2000 platform. The solid circles in (b-d) are colored according to assays, sample collection strategies, and sample collection countries, respectively. [file 12866_2021_2321_MOESM3_ESM.pdf]
