# Supplementary material for: MetaGeneBank: a standardized database to study deep sequenced metagenomic data from human fecal specimen
Source: BMC Microbiol. 2021 Sep 30;21:263. doi: 10.1186/s12866-021-02321-z (PMC8485520; doi:10.1186/s12866-021-02321-z)
Supplement: Supplementary file 4 — Additional file 4 : Table S1. The distribution of samples per disease and country. [file 12866_2021_2321_MOESM4_ESM.docx]

**Table S1**. The distribution of samples per disease and country.

| Country | Disease | | | | | | | | | | |
| --- | --- | --- | --- | --- | --- | --- | --- | --- | --- | --- | --- |
|  | AS | ACD | CFS | CC | IBD | LC | Obesity | RA | T1D | T2D | NAFLD |
| China | 211 | 385 | 100 |  | 122 | 237 |  | 137 |  | 504 |  |
| Austria |  |  |  | 310 |  |  |  |  |  |  |  |
| USA |  |  |  |  | 356 |  |  |  |  |  |  |
| Spain |  |  |  |  | 258 |  |  |  |  | 142 |  |
| Denmark |  |  |  |  | 262 |  | 292 |  |  | 111 |  |
| Sweden |  |  |  |  |  |  |  |  |  | 130 |  |
| Estonia |  |  |  |  |  |  |  |  | 21 | 1 |  |
| Finland |  |  |  |  |  |  |  |  | 107 | 1 |  |
| France |  |  |  |  |  |  |  |  |  | 1 |  |
| Germany |  |  |  |  |  |  |  |  |  | 4 |  |
| Hungary |  |  |  |  |  |  |  |  |  | 1 |  |
| Iceland |  |  |  |  |  |  |  |  |  | 1 |  |
| Norway |  |  |  |  |  |  |  |  |  | 1 |  |
| Slovakia |  |  |  |  |  |  |  |  |  | 1 |  |
| Yugoslavia |  |  |  |  |  |  |  |  |  | 2 |  |
| Not available |  |  |  |  |  |  |  |  |  | 102 | 86 |
