# Supplementary material for: MetaGeneBank: a standardized database to study deep sequenced metagenomic data from human fecal specimen
Source: BMC Microbiol. 2021 Sep 30;21:263. doi: 10.1186/s12866-021-02321-z (PMC8485520; doi:10.1186/s12866-021-02321-z)
Supplement: Supplementary file 5 — Additional file 5 : Table S2. The candidate values for each parameter that can be used in searching. [file 12866_2021_2321_MOESM5_ESM.docx]

| Disease | alkylosing spondylitis (AS), atherosclerotic cardiovascular disease (ACD), chronic fatigue syndrome (CFS), colorectal carcinoma (CC), colorectal adenoma, inflammatory bowel disease (IBD), liver cirrhosis (LC), Obesity, rheumatoid arthritis (RA), type 1 diabetes (T1D), type 2 diabetes (T2D), ulcerative colitis (UC), Crhon's disease (CD), non-alcoholic fatty liver disease (NAFLD) |
| --- | --- |
| Study | AS1, ACD1, CFS1, CC1, IBD1, IBD2, IBD3, IBD4, LC1, Obesity1, RA1, T1D1, T2D1, T2D2, T2D3, NAFLD1 |
| Assay | AS1.as1, ACD1.as1, CFS1.as1, CC1.as1, IBD1.as1, IBD2.as1, IBD3.as1, IBD4.as1, LC1.as1, Obesity1.as1, RA1.as1, T1D1.as1, T2D1.as1, T2D1.as2, T2D2.as1, T2D3.as1, NAFLD1.as1 |
| Technology Platform | Illumina Genome Analyzer II, Illumina Hiseq 2000, Illumina Hiseq 4000, Illumina Genome Analyzer IIx, Illumina Hiseq 2500 |
| Library Layout | PAIRED, SINGLE |
| Sex | male, female |

**Table S2**. The candidate values for each parameter that can be used in searching.
