# Supplementary material for: MetaGeneBank: a standardized database to study deep sequenced metagenomic data from human fecal specimen
Source: BMC Microbiol. 2021 Sep 30;21:263. doi: 10.1186/s12866-021-02321-z (PMC8485520; doi:10.1186/s12866-021-02321-z)
Supplement: Supplementary file 6 — Additional file 6 : Table S3. A document associating KO annotations with GO Terms. [file 12866_2021_2321_MOESM6_ESM.docx]

**Table S3**. A document associating KO annotations with GO Terms.

| **KEGG ID** | **GO Terms** | **GO ID** |
| --- | --- | --- |
| KEGG:R00004 | GO:inorganic diphosphatase activity | GO:0004427 |
| KEGG:R00005 | GO:allophanate hydrolase activity | GO:0004039 |
| KEGG:R00012 | GO:guanosine-triphosphate guanylyltransferase activity | GO:0047351 |
| KEGG:R00013 | GO:tartronate-semialdehyde synthase activity | GO:0009028 |
| KEGG:R00023 | GO:hyponitrite reductase activity | GO:0047999 |
| KEGG:R00025 | GO:nitronate monooxygenase (FMN-linked) activity | GO:0036434 |
| KEGG:R00027 | GO:benzoin aldolase activity | GO:0047695 |
| KEGG:R00032 | GO:beta-carotene 15,15'-monooxygenase activity | GO:0003834 |
| KEGG:R00036 | GO:porphobilinogen synthase activity | GO:0004655 |
| KEGG:R00038 | GO:propioin synthase activity | GO:0050217 |
| KEGG:R00042 | GO:4-hydroxyphenylpyruvate oxidase activity | GO:0018490 |
| KEGG:R00043 | GO:lignostilbene alpha beta-dioxygenase activity | GO:0050054 |
| KEGG:R00044 | GO:columbamine oxidase activity | GO:0050455 |
| KEGG:R00048 | GO:hydroxybutyrate-dimer hydrolase activity | GO:0047989 |
| KEGG:R00049 | GO:beta-glucogallin O-galloyltransferase activity | GO:0047201 |
| KEGG:R00053 | GO:tannase activity | GO:0050318 |
| KEGG:R00054 | GO:orsellinate-depside hydrolase activity | GO:0050160 |
| KEGG:R00060 | GO:sulochrin oxidase [(+)-bisdechlorogeodin-forming] activity | GO:0047064 |
| KEGG:R00061 | GO:sulochrin oxidase [(-)-bisdechlorogeodin-forming] activity | GO:0047065 |
| KEGG:R00062 | GO:bilirubin-glucuronoside glucuronosyltransferase activity | GO:0047278 |
| KEGG:R00063 | GO:sinapoylglucose-sinapoylglucose O-sinapoyltransferase activity | GO:0047158 |
| KEGG:R00066 | GO:riboflavin synthase activity | GO:0004746 |
| KEGG:R00075 | GO:nicotianamine synthase activity | GO:0030410 |
| KEGG:R00083 | GO:hydroquinone:oxygen oxidoreductase activity | GO:0052716 |
| KEGG:R00084 | GO:hydroxymethylbilane synthase activity | GO:0004418 |
| KEGG:R00090 | GO:NADH peroxidase activity | GO:0016692 |
| KEGG:R00093 | GO:glutamate synthase (NADH) activity | GO:0016040 |
| KEGG:R00099 | GO:cob(II)alamin reductase activity | GO:0050453 |
| KEGG:R00103 | GO:NADH pyrophosphatase activity | GO:0035529 |
| KEGG:R00104 | GO:NAD+ kinase activity | GO:0003951 |
| KEGG:R00105 | GO:NADH kinase activity | GO:0042736 |
| KEGG:R00112 | GO:NAD(P)+ transhydrogenase activity | GO:0008746 |
| KEGG:R00113 | GO:NADPH peroxidase activity | GO:0050137 |
| KEGG:R00114 | GO:glutamate synthase (NADPH) activity | GO:0004355 |
| KEGG:R00120 | GO:glutathione oxidase activity | GO:0047950 |
| KEGG:R00125 | GO:bis(5'-nucleosyl)-tetraphosphatase (symmetrical) activity | GO:0008803 |
| KEGG:R00129 | GO:ATP-dependent NAD(P)H-hydrate dehydratase activity | GO:0047453 |
| KEGG:R00130 | GO:dephospho-CoA kinase activity | GO:0004140 |
| KEGG:R00134 | GO:formate dehydrogenase (NADP+) activity | GO:0047899 |
| KEGG:R00138 | GO:triphosphatase activity | GO:0050355 |
| KEGG:R00141 | GO:ammonia kinase activity | GO:0047666 |
| KEGG:R00149 | GO:carbamoyl-phosphate synthase (ammonia) activity | GO:0004087 |
| KEGG:R00155 | GO:uridine-diphosphatase activity | GO:0045134 |
| KEGG:R00161 | GO:FMN adenylyltransferase activity | GO:0003919 |
| KEGG:R00162 | GO:MAP kinase activity | GO:0004707 |
| KEGG:R00175 | GO:adenosylmethionine hydrolase activity | GO:0047626 |
| KEGG:R00178 | GO:adenosylmethionine decarboxylase activity | GO:0004014 |
| KEGG:R00179 | GO:1-aminocyclopropane-1-carboxylate synthase activity | GO:0016847 |
| KEGG:R00180 | GO:adenosylmethionine cyclotransferase activity | GO:0047625 |
| KEGG:R00182 | GO:AMP nucleosidase activity | GO:0008714 |
| KEGG:R00187 | GO:bis(5'-adenosyl)-triphosphatase activity | GO:0047710 |
| KEGG:R00193 | GO:S-adenosylhomocysteine deaminase activity | GO:0050270 |
| KEGG:R00199 | GO:pyruvate, water dikinase activity | GO:0008986 |
| KEGG:R00206 | GO:pyruvate, phosphate dikinase activity | GO:0050242 |
| KEGG:R00207 | GO:pyruvate oxidase activity | GO:0047112 |
| KEGG:R00208 | GO:phosphoenolpyruvate phosphatase activity | GO:0050189 |
| KEGG:R00209 | GO:pyruvate dehydrogenase (NAD+) activity | GO:0034604 |
| KEGG:R00210 | GO:pyruvate dehydrogenase (NADP+) activity | GO:0050243 |
| KEGG:R00211 | GO:pyruvate oxidase (CoA-acetylating) activity | GO:0050244 |
| KEGG:R00213 | GO:carbamoyl-serine ammonia-lyase activity | GO:0047766 |
| KEGG:R00214 | GO:malate dehydrogenase (decarboxylating) (NAD+) activity | GO:0004471 |
| KEGG:R00215 | GO:D-malate dehydrogenase (decarboxylating) activity | GO:0046553 |
| KEGG:R00216 | GO:malate dehydrogenase (decarboxylating) (NADP+) activity | GO:0004473 |
| KEGG:R00217 | GO:oxaloacetate decarboxylase activity | GO:0008948 |
| KEGG:R00227 | GO:acetyl-CoA hydrolase activity | GO:0003986 |
| KEGG:R00237 | GO:(3S)-citramalyl-CoA lyase activity | GO:0047777 |
| KEGG:R00239 | GO:glutamate 5-kinase activity | GO:0004349 |
| KEGG:R00241 | GO:glutamate 1-kinase activity | GO:0047944 |
| KEGG:R00251 | GO:5-oxoprolinase (ATP-hydrolyzing) activity | GO:0017168 |
| KEGG:R00253 | GO:glutamate-ammonia ligase activity | GO:0004356 |
| KEGG:R00258 | GO:L-alanine:2-oxoglutarate aminotransferase activity | GO:0004021 |
| KEGG:R00259 | GO:acetyl-CoA:L-glutamate N-acetyltransferase activity | GO:0004042 |
| KEGG:R00260 | GO:glutamate racemase activity | GO:0008881 |
| KEGG:R00262 | GO:methylaspartate mutase activity | GO:0050097 |
| KEGG:R00265 | GO:oxoglutarate dehydrogenase (NADP+) activity | GO:0050164 |
| KEGG:R00271 | GO:homocitrate synthase activity | GO:0004410 |
| KEGG:R00272 | GO:2-oxoglutarate decarboxylase activity | GO:0008683 |
| KEGG:R00273 | GO:oxalate oxidase activity | GO:0050162 |
| KEGG:R00279 | GO:D-glutamate oxidase activity | GO:0047821 |
| KEGG:R00286 | GO:UDP-glucose 6-dehydrogenase activity | GO:0003979 |
| KEGG:R00289 | GO:UTP:glucose-1-phosphate uridylyltransferase activity | GO:0003983 |
| KEGG:R00293 | GO:UDP-glucose 4,6-dehydratase activity | GO:0050377 |
| KEGG:R00294 | GO:nitric oxide reductase activity | GO:0016966 |
| KEGG:R00297 | GO:D-2-hydroxy-acid dehydrogenase activity | GO:0047809 |
| KEGG:R00302 | GO:pyranose oxidase activity | GO:0050233 |
| KEGG:R00303 | GO:glucose-6-phosphatase activity | GO:0004346 |
| KEGG:R00304 | GO:glucose-1-phosphatase activity | GO:0008877 |
| KEGG:R00305 | GO:glucose dehydrogenase activity | GO:0004344 |
| KEGG:R00310 | GO:ferrochelatase activity | GO:0004325 |
| KEGG:R00318 | GO:phosphonoacetate hydrolase activity | GO:0047400 |
| KEGG:R00319 | GO:lactate 2-monooxygenase activity | GO:0050040 |
| KEGG:R00320 | GO:acetate kinase (diphosphate) activity | GO:0047601 |
| KEGG:R00324 | GO:acetylpyruvate hydrolase activity | GO:0018773 |
| KEGG:R00325 | GO:citramalate lyase activity | GO:0047776 |
| KEGG:R00327 | GO:6-acetylglucose deacetylase activity | GO:0047593 |
| KEGG:R00337 | GO:GDP-glucosidase activity | GO:0047917 |
| KEGG:R00338 | GO:oxaloacetase activity | GO:0030603 |
| KEGG:R00339 | GO:L(+)-tartrate dehydratase activity | GO:0008730 |
| KEGG:R00340 | GO:D(-)-tartrate dehydratase activity | GO:0047808 |
| KEGG:R00341 | GO:phosphoenolpyruvate carboxykinase (ATP) activity | GO:0004612 |
| KEGG:R00343 | GO:malate dehydrogenase (NADP+) activity | GO:0046554 |
| KEGG:R00344 | GO:pyruvate carboxylase activity | GO:0004736 |
| KEGG:R00345 | GO:phosphoenolpyruvate carboxylase activity | GO:0008964 |
| KEGG:R00346 | GO:phosphoenolpyruvate carboxykinase (diphosphate) activity | GO:0030585 |
| KEGG:R00351 | GO:citrate synthase activity | GO:0036440 |
| KEGG:R00352 | GO:ATP citrate synthase activity | GO:0003878 |
| KEGG:R00355 | GO:L-aspartate:2-oxoglutarate aminotransferase activity | GO:0004069 |
| KEGG:R00360 | GO:malate oxidase activity | GO:0019157 |
| KEGG:R00362 | GO:citrate (pro-3S)-lyase activity | GO:0008815 |
| KEGG:R00363 | GO:oxaloacetate tautomerase activity | GO:0050163 |
| KEGG:R00367 | GO:glycine N-methyltransferase activity | GO:0017174 |
| KEGG:R00368 | GO:strombine dehydrogenase activity | GO:0050305 |
| KEGG:R00371 | GO:glycine C-acetyltransferase activity | GO:0008890 |
| KEGG:R00373 | GO:glycine-oxaloacetate transaminase activity | GO:0047303 |
| KEGG:R00394 | GO:acid-CoA ligase (GDP-forming) activity | GO:0047612 |
| KEGG:R00398 | GO:alanopine dehydrogenase activity | GO:0047636 |
| KEGG:R00401 | GO:alanine racemase activity | GO:0008784 |
| KEGG:R00402 | GO:fumarate reductase (NADH) activity | GO:0016156 |
| KEGG:R00405 | GO:succinate-CoA ligase (ADP-forming) activity | GO:0004775 |
| KEGG:R00407 | GO:succinyl-CoA hydrolase activity | GO:0004778 |
| KEGG:R00409 | GO:methylisocitrate lyase activity | GO:0046421 |
| KEGG:R00411 | GO:succinylglutamate desuccinylase activity | GO:0009017 |
| KEGG:R00416 | GO:UDP-N-acetylglucosamine diphosphorylase activity | GO:0003977 |
| KEGG:R00421 | GO:UDP-N-acetylglucosamine 6-dehydrogenase activity | GO:0047004 |
| KEGG:R00424 | GO:GTP cyclohydrolase I activity | GO:0003934 |
| KEGG:R00425 | GO:GTP cyclohydrolase II activity | GO:0003935 |
| KEGG:R00426 | GO:GTP diphosphatase activity | GO:0036219 |
| KEGG:R00432 | GO:succinate-CoA ligase (GDP-forming) activity | GO:0004776 |
| KEGG:R00447 | GO:L-lysine oxidase activity | GO:0050029 |
| KEGG:R00448 | GO:L-lysine 6-monooxygenase (NADPH) activity | GO:0047091 |
| KEGG:R00449 | GO:lysine 2-monooxygenase activity | GO:0050067 |
| KEGG:R00451 | GO:diaminopimelate decarboxylase activity | GO:0008836 |
| KEGG:R00452 | GO:D-lysopine dehydrogenase activity | GO:0047827 |
| KEGG:R00453 | GO:lysine-pyruvate 6-transaminase activity | GO:0050065 |
| KEGG:R00457 | GO:L-lysine 6-transaminase activity | GO:0045484 |
| KEGG:R00461 | GO:lysine 2,3-aminomutase activity | GO:0050066 |
| KEGG:R00462 | GO:lysine decarboxylase activity | GO:0008923 |
| KEGG:R00463 | GO:L-lysine-lactamase activity | GO:0050028 |
| KEGG:R00466 | GO:glyoxylate oxidase activity | GO:0047969 |
| KEGG:R00468 | GO:glyoxylate dehydrogenase (acylating) activity | GO:0047968 |
| KEGG:R00469 | GO:ureidoglycolate hydrolase activity | GO:0004848 |
| KEGG:R00472 | GO:malate synthase activity | GO:0004474 |
| KEGG:R00474 | GO:2-hydroxy-3-oxoadipate synthase activity | GO:0050439 |
| KEGG:R00476 | GO:glycolate dehydrogenase activity | GO:0019154 |
| KEGG:R00477 | GO:oxalomalate lyase activity | GO:0050204 |
| KEGG:R00478 | GO:3-hydroxyaspartate aldolase activity | GO:0047562 |
| KEGG:R00479 | GO:isocitrate lyase activity | GO:0004451 |
| KEGG:R00480 | GO:aspartate kinase activity | GO:0004072 |
| KEGG:R00482 | GO:aspartate-ammonia ligase (ADP-forming) activity | GO:0047478 |
| KEGG:R00484 | GO:ureidosuccinase activity | GO:0050386 |
| KEGG:R00486 | GO:cyanoalanine nitrilase activity | GO:0047427 |
| KEGG:R00487 | GO:aspartate N-acetyltransferase activity | GO:0017188 |
| KEGG:R00491 | GO:aspartate racemase activity | GO:0047689 |
| KEGG:R00494 | GO:glutathione hydrolase activity | GO:0036374 |
| KEGG:R00497 | GO:glutathione synthase activity | GO:0004363 |
| KEGG:R00499 | GO:S-succinylglutathione hydrolase activity | GO:0050273 |
| KEGG:R00502 | GO:UTP:galactose-1-phosphate uridylyltransferase activity | GO:0017103 |
| KEGG:R00504 | GO:UDP-galactose-UDP-N-acetylglucosamine galactose phosphotransferase activity | GO:0047357 |
| KEGG:R00512 | GO:CMP kinase activity | GO:0036430 |
| KEGG:R00518 | GO:formate kinase activity | GO:0047900 |
| KEGG:R00519 | GO:formate dehydrogenase (NAD+) activity | GO:0008863 |
| KEGG:R00521 | GO:formyl-CoA hydrolase activity | GO:0047901 |
| KEGG:R00522 | GO:oxalate decarboxylase activity | GO:0046564 |
| KEGG:R00524 | GO:formamidase activity | GO:0004328 |
| KEGG:R00525 | GO:N-formylglutamate deformylase activity | GO:0050129 |
| KEGG:R00527 | GO:S-formylglutathione hydrolase activity | GO:0018738 |
| KEGG:R00530 | GO:sulfate adenylyltransferase (ADP) activity | GO:0004780 |
| KEGG:R00531 | GO:adenylylsulfatase activity | GO:0047627 |
| KEGG:R00533 | GO:sulfite oxidase activity | GO:0008482 |
| KEGG:R00534 | GO:glycosulfatase activity | GO:0047966 |
| KEGG:R00540 | GO:nitrilase activity | GO:0000257 |
| KEGG:R00547 | GO:glutathione thiolesterase activity | GO:0047951 |
| KEGG:R00549 | GO:riboflavin kinase activity | GO:0008531 |
| KEGG:R00550 | GO:riboflavin phosphotransferase activity | GO:0050257 |
| KEGG:R00554 | GO:arginine kinase activity | GO:0004054 |
| KEGG:R00559 | GO:arginine 2-monooxygenase activity | GO:0047678 |
| KEGG:R00566 | GO:arginine decarboxylase activity | GO:0008792 |
| KEGG:R00574 | GO:cytidylate cyclase activity | GO:0047805 |
| KEGG:R00576 | GO:L-glutamine:pyruvate aminotransferase activity | GO:0047945 |
| KEGG:R00582 | GO:L-phosphoserine phosphatase activity | GO:0036424 |
| KEGG:R00584 | GO:diphosphate-serine phosphotransferase activity | GO:0047332 |
| KEGG:R00585 | GO:serine-pyruvate transaminase activity | GO:0004760 |
| KEGG:R00586 | GO:serine O-acetyltransferase activity | GO:0009001 |
| KEGG:R00601 | GO:tRNA (5-methylaminomethyl-2-thiouridylate)-methyltransferase activity | GO:0004808 |
| KEGG:R00603 | GO:dichloromethane dehalogenase activity | GO:0018834 |
| KEGG:R00604 | GO:formaldehyde dehydrogenase activity | GO:0018467 |
| KEGG:R00605 | GO:methanol dehydrogenase activity | GO:0050093 |
| KEGG:R00606 | GO:methylamine dehydrogenase (amicyanin) activity | GO:0052876 |
| KEGG:R00609 | GO:methylglutamate dehydrogenase activity | GO:0050099 |
| KEGG:R00610 | GO:sarcosine oxidase activity | GO:0008115 |
| KEGG:R00611 | GO:sarcosine dehydrogenase activity | GO:0008480 |
| KEGG:R00612 | GO:N6-methyl-lysine oxidase activity | GO:0050134 |
| KEGG:R00617 | GO:thiamine-phosphate kinase activity | GO:0009030 |
| KEGG:R00618 | GO:thiamin-triphosphatase activity | GO:0050333 |
| KEGG:R00630 | GO:carboxylic ester hydrolase activity | GO:0052689 |
| KEGG:R00643 | GO:L-galactonolactone oxidase activity | GO:0050024 |
| KEGG:R00652 | GO:methionine-glyoxylate transaminase activity | GO:0050094 |
| KEGG:R00653 | GO:formylmethionine deformylase activity | GO:0008463 |
| KEGG:R00655 | GO:methionine racemase activity | GO:0018111 |
| KEGG:R00656 | GO:methionine decarboxylase activity | GO:0050095 |
| KEGG:R00660 | GO:UDP-N-acetylglucosamine 1-carboxyvinyltransferase activity | GO:0008760 |
| KEGG:R00661 | GO:phosphoenolpyruvate mutase activity | GO:0050188 |
| KEGG:R00662 | GO:UTP diphosphatase activity | GO:0036221 |
| KEGG:R00664 | GO:ornithine N-benzoyltransferase activity | GO:0050156 |
| KEGG:R00666 | GO:N5-(carboxyethyl)ornithine synthase activity | GO:0047126 |
| KEGG:R00670 | GO:ornithine decarboxylase activity | GO:0004586 |
| KEGG:R00671 | GO:ornithine cyclodeaminase activity | GO:0008473 |
| KEGG:R00673 | GO:tryptophanase activity | GO:0009034 |
| KEGG:R00674 | GO:L-serine hydro-lyase (adding indole, L-tryptophan-forming) activity | GO:0052684 |
| KEGG:R00679 | GO:tryptophan 2-monooxygenase activity | GO:0050361 |
| KEGG:R00682 | GO:tryptophanamidase activity | GO:0050365 |
| KEGG:R00686 | GO:phenylalanine racemase (ATP-hydrolyzing) activity | GO:0047462 |
| KEGG:R00687 | GO:phenylalanine adenylyltransferase activity | GO:0050173 |
| KEGG:R00690 | GO:phenylalanine 2-monooxygenase activity | GO:0050172 |
| KEGG:R00692 | GO:L-phenylalanine:pyruvate aminotransferase activity | GO:0047312 |
| KEGG:R00693 | GO:phenylalanine N-acetyltransferase activity | GO:0050176 |
| KEGG:R00694 | GO:L-phenylalanine:2-oxoglutarate aminotransferase activity | GO:0080130 |
| KEGG:R00695 | GO:aspartate-phenylpyruvate transaminase activity | GO:0047319 |
| KEGG:R00704 | GO:D-lactate dehydrogenase activity | GO:0008720 |
| KEGG:R00715 | GO:saccharopine dehydrogenase (NAD+, L-lysine-forming) activity | GO:0004754 |
| KEGG:R00716 | GO:saccharopine dehydrogenase (NADP+, L-lysine-forming) activity | GO:0047130 |
| KEGG:R00720 | GO:ITP diphosphatase activity | GO:0036220 |
| KEGG:R00728 | GO:tyrosine phenol-lyase activity | GO:0050371 |
| KEGG:R00735 | GO:tyrosine-arginine ligase activity | GO:0050367 |
| KEGG:R00739 | GO:tyrosine 2,3-aminomutase activity | GO:0050368 |
| KEGG:R00743 | GO:malonate CoA-transferase activity | GO:0050078 |
| KEGG:R00747 | GO:phosphonoacetaldehyde hydrolase activity | GO:0050194 |
| KEGG:R00748 | GO:ethanolamine-phosphate phospho-lyase activity | GO:0050459 |
| KEGG:R00750 | GO:4-hydroxy-2-oxovalerate aldolase activity | GO:0008701 |
| KEGG:R00753 | GO:lactate aldolase activity | GO:0050041 |
| KEGG:R00765 | GO:glucosamine-6-phosphate deaminase activity | GO:0004342 |
| KEGG:R00768 | GO:glutamine-fructose-6-phosphate transaminase (isomerizing) activity | GO:0004360 |
| KEGG:R00774 | GO:urea carboxylase activity | GO:0004847 |
| KEGG:R00775 | GO:guanidinoacetase activity | GO:0047970 |
| KEGG:R00776 | GO:ureidoglycolate lyase activity | GO:0050385 |
| KEGG:R00777 | GO:amidinoaspartase activity | GO:0047660 |
| KEGG:R00778 | GO:cyanamide hydratase activity | GO:0018820 |
| KEGG:R00787 | GO:nitrite reductase [NAD(P)H] activity | GO:0008942 |
| KEGG:R00789 | GO:nitrite reductase [NAD(P)H] activity | GO:0008942 |
| KEGG:R00793 | GO:hydroxylamine oxidase activity | GO:0047991 |
| KEGG:R00799 | GO:nitroalkane oxidase activity | GO:0052664 |
| KEGG:R00802 | GO:sucrose alpha-glucosidase activity | GO:0004575 |
| KEGG:R00804 | GO:sugar-phosphatase activity | GO:0050308 |
| KEGG:R00805 | GO:sucrose-phosphate phosphatase activity | GO:0050307 |
| KEGG:R00821 | GO:o-pyrocatechuate decarboxylase activity | GO:0050150 |
| KEGG:R00822 | GO:protocatechuate decarboxylase activity | GO:0050223 |
| KEGG:R00828 | GO:2-nitrophenol 2-monooxygenase activity | GO:0047549 |
| KEGG:R00830 | GO:5-aminolevulinate synthase activity | GO:0003870 |
| KEGG:R00833 | GO:methylmalonyl-CoA mutase activity | GO:0004494 |
| KEGG:R00834 | GO:aldose-6-phosphate reductase (NADPH) activity | GO:0047641 |
| KEGG:R00842 | GO:glycerol-3-phosphate dehydrogenase [NAD+] activity | GO:0004367 |
| KEGG:R00844 | GO:glycerol-3-phosphate dehydrogenase [NADP+] activity | GO:0036439 |
| KEGG:R00845 | GO:glycerol-3-phosphate 1-dehydrogenase [NADP+] activity | GO:0047014 |
| KEGG:R00846 | GO:glycerol-3-phosphate oxidase activity | GO:0004369 |
| KEGG:R00847 | GO:glycerol kinase activity | GO:0004370 |
| KEGG:R00850 | GO:glycerol-3-phosphate-glucose phosphotransferase activity | GO:0047327 |
| KEGG:R00853 | GO:sn-glycerol-3-phosphate 2-alpha-galactosyltransferase activity | GO:0047221 |
| KEGG:R00854 | GO:sn-glycerol-3-phosphate 1-galactosyltransferase activity | GO:0047279 |
| KEGG:R00855 | GO:CDP-glycerol diphosphatase activity | GO:0047734 |
| KEGG:R00856 | GO:glycerol-3-phosphate cytidylyltransferase activity | GO:0047348 |
| KEGG:R00870 | GO:mannitol 2-dehydrogenase (NADP+) activity | GO:0050085 |
| KEGG:R00873 | GO:fructose 5-dehydrogenase activity | GO:0047904 |
| KEGG:R00874 | GO:glucose-fructose oxidoreductase activity | GO:0047061 |
| KEGG:R00877 | GO:mannose isomerase activity | GO:0050089 |
| KEGG:R00880 | GO:GDP-mannose 6-dehydrogenase activity | GO:0047919 |
| KEGG:R00883 | GO:mannose-1-phosphate guanylyltransferase (GDP) activity | GO:0008928 |
| KEGG:R00885 | GO:mannose-1-phosphate guanylyltransferase activity | GO:0004475 |
| KEGG:R00888 | GO:GDP-mannose 4,6-dehydratase activity | GO:0008446 |
| KEGG:R00892 | GO:cystine reductase activity | GO:0050456 |
| KEGG:R00893 | GO:cysteine dioxygenase activity | GO:0017172 |
| KEGG:R00894 | GO:glutamate-cysteine ligase activity | GO:0004357 |
| KEGG:R00906 | GO:beta-alanopine dehydrogenase activity | GO:0047697 |
| KEGG:R00909 | GO:N-acetyl-beta-alanine deacetylase activity | GO:0050117 |
| KEGG:R00913 | GO:guanidinopropionase activity | GO:0047972 |
| KEGG:R00919 | GO:acryloyl-CoA reductase (NADP+) activity | GO:0043957 |
| KEGG:R00931 | GO:2-methylcitrate synthase activity | GO:0050440 |
| KEGG:R00932 | GO:2-hydroxyglutarate synthase activity | GO:0019142 |
| KEGG:R00941 | GO:formyltetrahydrofolate dehydrogenase activity | GO:0016155 |
| KEGG:R00944 | GO:formyltetrahydrofolate deformylase activity | GO:0008864 |
| KEGG:R00946 | GO:methionine synthase activity | GO:0008705 |
| KEGG:R00948 | GO:glucose-1-phosphate adenylyltransferase activity | GO:0008878 |
| KEGG:R00949 | GO:phosphoglucokinase activity | GO:0050190 |
| KEGG:R00954 | GO:glucose-1-phosphate guanylyltransferase activity | GO:0047344 |
| KEGG:R00955 | GO:UDP-glucose:hexose-1-phosphate uridylyltransferase activity | GO:0008108 |
| KEGG:R00956 | GO:glucose-1-phosphate cytidylyltransferase activity | GO:0047343 |
| KEGG:R00965 | GO:orotidine-5'-phosphate decarboxylase activity | GO:0004590 |
| KEGG:R00966 | GO:uracil phosphoribosyltransferase activity | GO:0004845 |
| KEGG:R00973 | GO:uracil-5-carboxylate decarboxylase activity | GO:0050382 |
| KEGG:R00975 | GO:dihydrouracil oxidase activity | GO:0047857 |
| KEGG:R00979 | GO:anthranilate adenylyltransferase activity | GO:0047671 |
| KEGG:R00980 | GO:anthranilate 3-monooxygenase (deaminating) activity | GO:0018672 |
| KEGG:R00982 | GO:anthranilate-CoA ligase activity | GO:0018860 |
| KEGG:R00983 | GO:2,3-dihydroxyindole 2,3-dioxygenase activity | GO:0047528 |
| KEGG:R00984 | GO:anthranilate N-methyltransferase activity | GO:0030774 |
| KEGG:R00989 | GO:anthranilate N-malonyltransferase activity | GO:0047673 |
| KEGG:R00997 | GO:1-aminocyclopropane-1-carboxylate deaminase activity | GO:0008660 |
| KEGG:R00998 | GO:2-ethylmalate synthase activity | GO:0050438 |
| KEGG:R01001 | GO:cystathionine gamma-lyase activity | GO:0004123 |
| KEGG:R01011 | GO:glycerone kinase activity | GO:0004371 |
| KEGG:R01012 | GO:phosphoenolpyruvate-glycerone phosphotransferase activity | GO:0047324 |
| KEGG:R01013 | GO:glycerone-phosphate O-acyltransferase activity | GO:0016287 |
| KEGG:R01014 | GO:ketotetrose-phosphate aldolase activity | GO:0050014 |
| KEGG:R01015 | GO:triose-phosphate isomerase activity | GO:0004807 |
| KEGG:R01016 | GO:methylglyoxal synthase activity | GO:0008929 |
| KEGG:R01021 | GO:choline kinase activity | GO:0004103 |
| KEGG:R01023 | GO:choline O-acetyltransferase activity | GO:0004102 |
| KEGG:R01025 | GO:choline dehydrogenase activity | GO:0008812 |
| KEGG:R01027 | GO:choline sulfotransferase activity | GO:0047754 |
| KEGG:R01028 | GO:choline-sulfatase activity | GO:0047753 |
| KEGG:R01034 | GO:glycerol dehydrogenase [NAD+] activity | GO:0008888 |
| KEGG:R01039 | GO:glycerol 2-dehydrogenase (NADP+) activity | GO:0047953 |
| KEGG:R01043 | GO:glycerol-2-phosphatase activity | GO:0047954 |
| KEGG:R01044 | GO:diphosphate-glycerol phosphotransferase activity | GO:0047331 |
| KEGG:R01045 | GO:glycerol dehydrogenase (acceptor) activity | GO:0047955 |
| KEGG:R01049 | GO:ribose phosphate diphosphokinase activity | GO:0004749 |
| KEGG:R01050 | GO:phosphoribokinase activity | GO:0050195 |
| KEGG:R01052 | GO:ribose-5-phosphate adenylyltransferase activity | GO:0047345 |
| KEGG:R01053 | GO:ribose-5-phosphate-ammonia ligase activity | GO:0050260 |
| KEGG:R01055 | GO:pseudouridylate synthase activity | GO:0004730 |
| KEGG:R01056 | GO:ribose-5-phosphate isomerase activity | GO:0004751 |
| KEGG:R01058 | GO:glyceraldehyde-3-phosphate dehydrogenase (NADP+) (non-phosphorylating) activity | GO:0008886 |
| KEGG:R01059 | GO:triokinase activity | GO:0050354 |
| KEGG:R01064 | GO:2-dehydro-3-deoxy-6-phosphogalactonate aldolase activity | GO:0008674 |
| KEGG:R01066 | GO:deoxyribose-phosphate aldolase activity | GO:0004139 |
| KEGG:R01069 | GO:tagatose-bisphosphate aldolase activity | GO:0009025 |
| KEGG:R01072 | GO:amidophosphoribosyltransferase activity | GO:0004044 |
| KEGG:R01073 | GO:anthranilate phosphoribosyltransferase activity | GO:0004048 |
| KEGG:R01078 | GO:biotin synthase activity | GO:0004076 |
| KEGG:R01079 | GO:ribose 1-dehydrogenase (NADP+) activity | GO:0050259 |
| KEGG:R01080 | GO:uridine nucleosidase activity | GO:0045437 |
| KEGG:R01081 | GO:ribose isomerase activity | GO:0050261 |
| KEGG:R01082 | GO:fumarate hydratase activity | GO:0004333 |
| KEGG:R01083 | GO:N6-(1,2-dicarboxyethyl)AMP AMP-lyase (fumarate-forming) activity | GO:0004018 |
| KEGG:R01087 | GO:maleate isomerase activity | GO:0050076 |
| KEGG:R01089 | GO:leucine N-acetyltransferase activity | GO:0050050 |
| KEGG:R01090 | GO:L-leucine transaminase activity | GO:0052654 |
| KEGG:R01091 | GO:leucine 2,3-aminomutase activity | GO:0050047 |
| KEGG:R01092 | GO:galactokinase activity | GO:0004335 |
| KEGG:R01103 | GO:raffinose alpha-galactosidase activity | GO:0052692 |
| KEGG:R01108 | GO:glutathione dehydrogenase (ascorbate) activity | GO:0045174 |
| KEGG:R01121 | GO:diphosphomevalonate decarboxylase activity | GO:0004163 |
| KEGG:R01123 | GO:isopentenyl-diphosphate delta-isomerase activity | GO:0004452 |
| KEGG:R01128 | GO:inosinate nucleosidase activity | GO:0047723 |
| KEGG:R01132 | GO:hypoxanthine phosphoribosyltransferase activity | GO:0004422 |
| KEGG:R01134 | GO:GMP reductase activity | GO:0003920 |
| KEGG:R01135 | GO:adenylosuccinate synthase activity | GO:0004019 |
| KEGG:R01142 | GO:methane monooxygenase activity | GO:0015049 |
| KEGG:R01143 | GO:methane monooxygenase activity | GO:0015049 |
| KEGG:R01144 | GO:4-methyloxaloacetate esterase activity | GO:0047583 |
| KEGG:R01145 | GO:monomethyl-sulfatase activity | GO:0050106 |
| KEGG:R01146 | GO:methanol ferricytochrome-c oxidoreductase activity | GO:0052930 |
| KEGG:R01149 | GO:D-alanine gamma-glutamyltransferase activity | GO:0047811 |
| KEGG:R01150 | GO:D-alanine-D-alanine ligase activity | GO:0008716 |
| KEGG:R01152 | GO:N-carbamoylputrescine amidase activity | GO:0050126 |
| KEGG:R01153 | GO:putrescine N-methyltransferase activity | GO:0030750 |
| KEGG:R01156 | GO:acetylputrescine deacetylase activity | GO:0047609 |
| KEGG:R01157 | GO:agmatinase activity | GO:0008783 |
| KEGG:R01160 | GO:histidine N-acetyltransferase activity | GO:0047981 |
| KEGG:R01161 | GO:L-histidine:2-oxoglutarate aminotransferase activity | GO:0008110 |
| KEGG:R01169 | GO:N-alpha,N-alpha,N-alpha-trimethyl-L-histidine biosynthesis from histidine | GO:0052707 |
| KEGG:R01174 | GO:phosphate butyryltransferase activity | GO:0050182 |
| KEGG:R01178 | GO:butyryl-CoA dehydrogenase activity | GO:0004085 |
| KEGG:R01180 | GO:3-ethylmalate synthase activity | GO:0050441 |
| KEGG:R01181 | GO:isobutyryl-CoA mutase activity | GO:0047727 |
| KEGG:R01183 | GO:inositol 2-dehydrogenase activity | GO:0050112 |
| KEGG:R01184 | GO:inositol oxygenase activity | GO:0050113 |
| KEGG:R01188 | GO:inositol 1-methyltransferase activity | GO:0030741 |
| KEGG:R01189 | GO:inositol 3-methyltransferase activity | GO:0030740 |
| KEGG:R01190 | GO:inositol 4-methyltransferase activity | GO:0030787 |
| KEGG:R01193 | GO:glycerophosphoinositol glycerophosphodiesterase activity | GO:0047395 |
| KEGG:R01196 | GO:pyruvate synthase activity | GO:0019164 |
| KEGG:R01200 | GO:N-acetylglucosamine deacetylase activity | GO:0050119 |
| KEGG:R01201 | GO:N-acetylglucosamine kinase activity | GO:0045127 |
| KEGG:R01202 | GO:N-acetylhexosamine 1-dehydrogenase activity | GO:0050120 |
| KEGG:R01203 | GO:N-acylhexosamine oxidase activity | GO:0050122 |
| KEGG:R01204 | GO:glucosamine N-acetyltransferase activity | GO:0047932 |
| KEGG:R01211 | GO:dimethylmalate dehydrogenase activity | GO:0047867 |
| KEGG:R01213 | GO:2-isopropylmalate synthase activity | GO:0003852 |
| KEGG:R01214 | GO:L-valine transaminase activity | GO:0052655 |
| KEGG:R01215 | GO:valine-pyruvate transaminase activity | GO:0009042 |
| KEGG:R01218 | GO:methylenetetrahydrofolate dehydrogenase (NAD+) activity | GO:0004487 |
| KEGG:R01220 | GO:methylenetetrahydrofolate dehydrogenase (NADP+) activity | GO:0004488 |
| KEGG:R01224 | GO:methylenetetrahydrofolate reductase (NAD(P)H) activity | GO:0004489 |
| KEGG:R01225 | GO:D-alanine 2-hydroxymethyltransferase activity | GO:0050413 |
| KEGG:R01229 | GO:guanine phosphoribosyltransferase activity | GO:0052657 |
| KEGG:R01238 | GO:4-hydroxybenzoate decarboxylase activity | GO:0018799 |
| KEGG:R01239 | GO:phenol O-methyltransferase activity | GO:0030736 |
| KEGG:R01255 | GO:proline racemase activity | GO:0018112 |
| KEGG:R01266 | GO:beta-aspartyl-N-acetylglucosaminidase activity | GO:0047406 |
| KEGG:R01267 | GO:3-cyanoalanine hydratase activity | GO:0047558 |
| KEGG:R01269 | GO:nicotinamide N-methyltransferase activity | GO:0008112 |
| KEGG:R01270 | GO:NMN nucleosidase activity | GO:0019160 |
| KEGG:R01271 | GO:nicotinamide phosphoribosyltransferase activity | GO:0047280 |
| KEGG:R01277 | GO:hexadecanal dehydrogenase (acylating) activity | GO:0047104 |
| KEGG:R01281 | GO:serine C-palmitoyltransferase activity | GO:0004758 |
| KEGG:R01291 | GO:S-ribosylhomocysteine lyase activity | GO:0043768 |
| KEGG:R01295 | GO:benzoate 4-monooxygenase activity | GO:0018664 |
| KEGG:R01300 | GO:4-hydroxybenzoate-CoA ligase activity | GO:0018859 |
| KEGG:R01301 | GO:4-hydroxybenzoyl-CoA thioesterase activity | GO:0018739 |
| KEGG:R01302 | GO:chorismate lyase activity | GO:0008813 |
| KEGG:R01304 | GO:4-hydroxybenzoate 4-O-beta-D-glucosyltransferase activity | GO:0047250 |
| KEGG:R01305 | GO:2,4'-dihydroxyacetophenone dioxygenase activity | GO:0047073 |
| KEGG:R01306 | GO:4-methoxybenzoate monooxygenase (O-demethylating) activity | GO:0018690 |
| KEGG:R01307 | GO:4-chlorobenzoate dehalogenase activity | GO:0047576 |
| KEGG:R01319 | GO:2-acylglycerophosphocholine O-acyltransferase activity | GO:0047190 |
| KEGG:R01320 | GO:phosphatidyl-N-dimethylethanolamine N-methyltransferase activity | GO:0080101 |
| KEGG:R01322 | GO:citrate-CoA ligase activity | GO:0047779 |
| KEGG:R01325 | GO:citrate dehydratase activity | GO:0047780 |
| KEGG:R01333 | GO:glycolaldehyde dehydrogenase activity | GO:0050569 |
| KEGG:R01334 | GO:phosphoglycolate phosphatase activity | GO:0008967 |
| KEGG:R01336 | GO:carboxymethyloxysuccinate lyase activity | GO:0047772 |
| KEGG:R01355 | GO:2,3-dimethylmalate lyase activity | GO:0047529 |
| KEGG:R01357 | GO:acetoacetate-CoA ligase activity | GO:0030729 |
| KEGG:R01358 | GO:acetoacetyl-CoA hydrolase activity | GO:0047603 |
| KEGG:R01360 | GO:hydroxymethylglutaryl-CoA lyase activity | GO:0004419 |
| KEGG:R01361 | GO:3-hydroxybutyrate dehydrogenase activity | GO:0003858 |
| KEGG:R01364 | GO:fumarylacetoacetase activity | GO:0004334 |
| KEGG:R01365 | GO:butyrate-acetoacetate CoA-transferase activity | GO:0047371 |
| KEGG:R01366 | GO:acetoacetate decarboxylase activity | GO:0047602 |
| KEGG:R01375 | GO:glutamine-phenylpyruvate transaminase activity | GO:0047316 |
| KEGG:R01376 | GO:tryptophan-phenylpyruvate transaminase activity | GO:0047299 |
| KEGG:R01381 | GO:glucuronate-1-phosphate uridylyltransferase activity | GO:0047350 |
| KEGG:R01384 | GO:UDP-glucuronate decarboxylase activity | GO:0048040 |
| KEGG:R01385 | GO:UDP-glucuronate 4-epimerase activity | GO:0050378 |
| KEGG:R01387 | GO:UDP-glucuronate 5'-epimerase activity | GO:0050379 |
| KEGG:R01393 | GO:hydroxypyruvate decarboxylase activity | GO:0047997 |
| KEGG:R01394 | GO:hydroxypyruvate isomerase activity | GO:0008903 |
| KEGG:R01396 | GO:lysine carbamoyltransferase activity | GO:0050068 |
| KEGG:R01397 | GO:aspartate carbamoyltransferase activity | GO:0004070 |
| KEGG:R01399 | GO:putrescine carbamoyltransferase activity | GO:0050231 |
| KEGG:R01408 | GO:cyanide hydratase activity | GO:0030196 |
| KEGG:R01417 | GO:agmatine kinase activity | GO:0047633 |
| KEGG:R01418 | GO:diguanidinobutanase activity | GO:0047854 |
| KEGG:R01422 | GO:benzoate-CoA ligase activity | GO:0018858 |
| KEGG:R01424 | GO:hippurate hydrolase activity | GO:0047980 |
| KEGG:R01425 | GO:acylagmatine amidase activity | GO:0047618 |
| KEGG:R01430 | GO:D-xylose 1-dehydrogenase (NADP+) activity | GO:0047837 |
| KEGG:R01437 | GO:valine decarboxylase activity | GO:0050390 |
| KEGG:R01447 | GO:lactate-malate transhydrogenase activity | GO:0050042 |
| KEGG:R01448 | GO:D-lactate-2-sulfatase activity | GO:0047825 |
| KEGG:R01450 | GO:lactate racemase activity | GO:0050043 |
| KEGG:R01459 | GO:cholesterol oxidase activity | GO:0016995 |
| KEGG:R01460 | GO:steryl-beta-glucosidase activity | GO:0050295 |
| KEGG:R01461 | GO:cholesterol O-acyltransferase activity | GO:0034736 |
| KEGG:R01465 | GO:L-threonine 3-dehydrogenase activity | GO:0008743 |
| KEGG:R01467 | GO:threonine racemase activity | GO:0018114 |
| KEGG:R01468 | GO:ethanolamine kinase activity | GO:0004305 |
| KEGG:R01473 | GO:UDP-arabinose 4-epimerase activity | GO:0050373 |
| KEGG:R01476 | GO:glucuronokinase activity | GO:0047940 |
| KEGG:R01481 | GO:L-glucuronate reductase activity | GO:0047939 |
| KEGG:R01485 | GO:aspartyltransferase activity | GO:0047690 |
| KEGG:R01504 | GO:(2,3-dihydroxybenzoyl)adenylate synthase activity | GO:0008668 |
| KEGG:R01505 | GO:2,3-dihydro-2,3-dihydroxybenzoate dehydrogenase activity | GO:0008667 |
| KEGG:R01506 | GO:2,3-dihydroxybenzoate 2,3-dioxygenase activity | GO:0047072 |
| KEGG:R01507 | GO:2,3-dihydroxybenzoate 3,4-dioxygenase activity | GO:0045133 |
| KEGG:R01508 | GO:3-hydroxybenzoate 2-monooxygenase activity | GO:0047563 |
| KEGG:R01511 | GO:3-phosphoglycerate phosphatase activity | GO:0047572 |
| KEGG:R01512 | GO:phosphoglycerate kinase activity | GO:0004618 |
| KEGG:R01514 | GO:glycerate kinase activity | GO:0008887 |
| KEGG:R01516 | GO:bisphosphoglycerate 2-phosphatase activity | GO:0004083 |
| KEGG:R01517 | GO:phosphoglycerate kinase (GTP) activity | GO:0050191 |
| KEGG:R01522 | GO:glucose oxidase activity | GO:0046562 |
| KEGG:R01523 | GO:phosphoribulokinase activity | GO:0008974 |
| KEGG:R01529 | GO:ribulose-phosphate 3-epimerase activity | GO:0004750 |
| KEGG:R01530 | GO:arabinose-5-phosphate isomerase activity | GO:0019146 |
| KEGG:R01533 | GO:diisopropyl-fluorophosphatase activity | GO:0047862 |
| KEGG:R01535 | GO:undecaprenyl-phosphate galactose phosphotransferase activity | GO:0047360 |
| KEGG:R01538 | GO:gluconate dehydratase activity | GO:0047929 |
| KEGG:R01540 | GO:altronate dehydratase activity | GO:0008789 |
| KEGG:R01541 | GO:2-dehydro-3-deoxygluconokinase activity | GO:0008673 |
| KEGG:R01543 | GO:2-dehydro-3-deoxy-D-gluconate 6-dehydrogenase activity | GO:0047000 |
| KEGG:R01550 | GO:isopropanol dehydrogenase (NADP+) activity | GO:0050009 |
| KEGG:R01553 | GO:acetone-cyanohydrin acetone-lyase (cyanide-forming) activity | GO:0052921 |
| KEGG:R01558 | GO:oxalate-CoA ligase activity | GO:0050203 |
| KEGG:R01559 | GO:succinyl-CoA:oxalate CoA-transferase | GO:0050161 |
| KEGG:R01563 | GO:N-carbamoylsarcosine amidase activity | GO:0050127 |
| KEGG:R01564 | GO:dimethylglycine oxidase activity | GO:0047866 |
| KEGG:R01565 | GO:dimethylglycine dehydrogenase activity | GO:0047865 |
| KEGG:R01566 | GO:creatinase activity | GO:0016980 |
| KEGG:R01573 | GO:D-arabinokinase activity | GO:0047814 |
| KEGG:R01576 | GO:3-deoxy-D-manno-octulosonate aldolase activity | GO:0047439 |
| KEGG:R01581 | GO:N-acyl-D-glutamate deacylase activity | GO:0047421 |
| KEGG:R01583 | GO:D-glutamate cyclase activity | GO:0047820 |
| KEGG:R01584 | GO:N-methylalanine dehydrogenase activity | GO:0050132 |
| KEGG:R01585 | GO:glutamate-methylamine ligase activity | GO:0047943 |
| KEGG:R01586 | GO:methylamine-glutamate N-methyltransferase activity | GO:0047148 |
| KEGG:R01587 | GO:N-methyl-2-oxoglutaramate hydrolase activity | GO:0050130 |
| KEGG:R01589 | GO:methylguanidinase activity | GO:0050098 |
| KEGG:R01594 | GO:arachidonate 8(R)-lipoxygenase activity | GO:0047677 |
| KEGG:R01596 | GO:arachidonate 12-lipoxygenase activity | GO:0004052 |
| KEGG:R01598 | GO:arachidonate-CoA ligase activity | GO:0047676 |
| KEGG:R01608 | GO:3-hydroxypropionate dehydrogenase (NAD+) activity | GO:0047565 |
| KEGG:R01609 | GO:3-aci-nitropropanoate oxidase activity | GO:0047557 |
| KEGG:R01616 | GO:4-coumarate-CoA ligase activity | GO:0016207 |
| KEGG:R01617 | GO:agmatine N4-coumaroyltransferase activity | GO:0047634 |
| KEGG:R01619 | GO:adenylylsulfate-ammonia adenylyltransferase activity | GO:0047352 |
| KEGG:R01628 | GO:3-hydroxybenzoate 4-monooxygenase activity | GO:0018668 |
| KEGG:R01633 | GO:(3S,4R)-3,4-dihydroxycyclohexa-1,5-diene-1,4-dicarboxylate dehydrogenase activity | GO:0047120 |
| KEGG:R01634 | GO:3,4-dihydroxyphthalate decarboxylase activity | GO:0047556 |
| KEGG:R01636 | GO:4-sulfobenzoate 3,4-dioxygenase activity | GO:0018621 |
| KEGG:R01639 | GO:xylulokinase activity | GO:0004856 |
| KEGG:R01644 | GO:4-hydroxybutyrate dehydrogenase activity | GO:0047577 |
| KEGG:R01659 | GO:dimethylallylcistransferase activity | GO:0047863 |
| KEGG:R01660 | GO:glucose-1,6-bisphosphate synthase activity | GO:0047933 |
| KEGG:R01665 | GO:dCMP kinase activity | GO:0036431 |
| KEGG:R01669 | GO:deoxycytidylate 5-hydroxymethyltransferase activity | GO:0047153 |
| KEGG:R01670 | GO:deoxycytidylate C-methyltransferase activity | GO:0050003 |
| KEGG:R01681 | GO:hypotaurine dehydrogenase activity | GO:0047822 |
| KEGG:R01683 | GO:tauropine dehydrogenase activity | GO:0050325 |
| KEGG:R01688 | GO:butyrate kinase activity | GO:0047761 |
| KEGG:R01694 | GO:sorbose 5-dehydrogenase (NADP+) activity | GO:0050287 |
| KEGG:R01695 | GO:L-sorbose oxidase activity | GO:0050035 |
| KEGG:R01696 | GO:sorbose dehydrogenase activity | GO:0050288 |
| KEGG:R01703 | GO:fatty acid peroxidase activity | GO:0047888 |
| KEGG:R01707 | GO:pyridoxal 4-dehydrogenase activity | GO:0050235 |
| KEGG:R01708 | GO:pyridoxine:NADP 4-dehydrogenase activity | GO:0050236 |
| KEGG:R01712 | GO:pyridoxamine-pyruvate transaminase activity | GO:0047300 |
| KEGG:R01713 | GO:pyridoxamine-oxaloacetate transaminase activity | GO:0019162 |
| KEGG:R01714 | GO:chorismate synthase activity | GO:0004107 |
| KEGG:R01715 | GO:chorismate mutase activity | GO:0004106 |
| KEGG:R01716 | GO:4-amino-4-deoxychorismate synthase activity | GO:0046820 |
| KEGG:R01717 | GO:isochorismate synthase activity | GO:0008909 |
| KEGG:R01720 | GO:nicotinate dehydrogenase activity | GO:0050138 |
| KEGG:R01721 | GO:nicotinate N-methyltransferase activity | GO:0008938 |
| KEGG:R01722 | GO:nicotinate-N-glucosyltransferase activity | GO:0050139 |
| KEGG:R01724 | GO:nicotinate phosphoribosyltransferase activity | GO:0004516 |
| KEGG:R01728 | GO:prephenate dehydrogenase (NAD+) activity | GO:0008977 |
| KEGG:R01730 | GO:prephenate dehydrogenase (NADP+) activity | GO:0004665 |
| KEGG:R01732 | GO:riboflavinase activity | GO:0050258 |
| KEGG:R01737 | GO:gluconokinase activity | GO:0046316 |
| KEGG:R01748 | GO:phosphoglycerate phosphatase activity | GO:0050192 |
| KEGG:R01751 | GO:tartrate decarboxylase activity | GO:0050319 |
| KEGG:R01754 | GO:L-arabinokinase activity | GO:0009702 |
| KEGG:R01759 | GO:L-arabinose:NADP reductase activity | GO:0032867 |
| KEGG:R01761 | GO:L-arabinose isomerase activity | GO:0008733 |
| KEGG:R01766 | GO:phenylserine aldolase activity | GO:0050179 |
| KEGG:R01771 | GO:homoserine kinase activity | GO:0004413 |
| KEGG:R01776 | GO:homoserine O-acetyltransferase activity | GO:0004414 |
| KEGG:R01777 | GO:homoserine O-succinyltransferase activity | GO:0008899 |
| KEGG:R01782 | GO:2-dehydro-3-deoxy-D-pentonate aldolase activity | GO:0047440 |
| KEGG:R01797 | GO:CDP-diacylglycerol diphosphatase activity | GO:0008715 |
| KEGG:R01801 | GO:CDP-diacylglycerol-glycerol-3-phosphate 3-phosphatidyltransferase activity | GO:0008444 |
| KEGG:R01802 | GO:CDP-diacylglycerol-inositol 3-phosphatidyltransferase activity | GO:0003881 |
| KEGG:R01806 | GO:N-acetylneuraminate 4-O-acetyltransferase activity | GO:0047185 |
| KEGG:R01811 | GO:N-acetylneuraminate lyase activity | GO:0008747 |
| KEGG:R01812 | GO:pteridine reductase activity | GO:0047040 |
| KEGG:R01816 | GO:anthranilate 3-monooxygenase activity | GO:0050478 |
| KEGG:R01817 | GO:mannose-6-phosphate 6-reductase activity | GO:0050088 |
| KEGG:R01818 | GO:phosphomannomutase activity | GO:0004615 |
| KEGG:R01825 | GO:erythrose-4-phosphate dehydrogenase activity | GO:0048001 |
| KEGG:R01826 | GO:3-deoxy-7-phosphoheptulonate synthase activity | GO:0003849 |
| KEGG:R01833 | GO:androst-4-ene-3,17-dione monooxygenase activity | GO:0047096 |
| KEGG:R01836 | GO:testosterone dehydrogenase (NAD+) activity | GO:0047035 |
| KEGG:R01838 | GO:testosterone 17-beta-dehydrogenase (NADP+) activity | GO:0047045 |
| KEGG:R01844 | GO:sedoheptulokinase activity | GO:0050277 |
| KEGG:R01850 | GO:hydrogen-sulfide S-acetyltransferase activity | GO:0047986 |
| KEGG:R01853 | GO:primary amine oxidase activity | GO:0008131 |
| KEGG:R01855 | GO:dGTP diphosphatase activity | GO:0036217 |
| KEGG:R01856 | GO:dGTPase activity | GO:0008832 |
| KEGG:R01862 | GO:aryl-acylamidase activity | GO:0047680 |
| KEGG:R01866 | GO:orotate reductase (NADPH) activity | GO:0050158 |
| KEGG:R01868 | GO:dihydroorotate dehydrogenase activity | GO:0004152 |
| KEGG:R01869 | GO:orotate reductase (NADH) activity | GO:0004589 |
| KEGG:R01872 | GO:quinate 3-dehydrogenase (NAD+) activity | GO:0030266 |
| KEGG:R01873 | GO:quinate dehydrogenase (quinone) activity | GO:0047519 |
| KEGG:R01879 | GO:deoxyuridine 2'-dioxygenase activity | GO:0047080 |
| KEGG:R01881 | GO:creatine kinase activity | GO:0004111 |
| KEGG:R01882 | GO:phosphoamidase activity | GO:0050187 |
| KEGG:R01883 | GO:guanidinoacetate N-methyltransferase activity | GO:0030731 |
| KEGG:R01884 | GO:creatininase activity | GO:0047789 |
| KEGG:R01888 | GO:kanamycin kinase activity | GO:0008910 |
| KEGG:R01889 | GO:aminoglycoside 6'-N-acetyltransferase activity | GO:0047663 |
| KEGG:R01891 | GO:ceramide cholinephosphotransferase activity | GO:0047493 |
| KEGG:R01895 | GO:ribitol 2-dehydrogenase activity | GO:0050255 |
| KEGG:R01896 | GO:D-xylulose reductase activity | GO:0046526 |
| KEGG:R01898 | GO:D-lyxose ketol-isomerase activity | GO:0047828 |
| KEGG:R01900 | GO:isocitrate hydro-lyase (cis-aconitate-forming) activity | GO:0052633 |
| KEGG:R01903 | GO:L-arabinitol 4-dehydrogenase activity | GO:0050019 |
| KEGG:R01904 | GO:L-xylulose reductase (NADP+) activity | GO:0050038 |
| KEGG:R01905 | GO:dehydro-L-gulonate decarboxylase activity | GO:0047842 |
| KEGG:R01908 | GO:oxalyl-CoA decarboxylase activity | GO:0008949 |
| KEGG:R01912 | GO:pyridoxine 5'-O-beta-D-glucosyltransferase activity | GO:0047231 |
| KEGG:R01914 | GO:spermidine oxidase (propane-1,3-diamine-forming) activity | GO:0052896 |
| KEGG:R01921 | GO:(S)-carnitine 3-dehydrogenase activity | GO:0047041 |
| KEGG:R01922 | GO:carnitinamidase activity | GO:0047773 |
| KEGG:R01925 | GO:carnitine dehydratase activity | GO:0008735 |
| KEGG:R01928 | GO:sphingosine beta-galactosyltransferase activity | GO:0047258 |
| KEGG:R01929 | GO:sphingosine cholinephosphotransferase activity | GO:0047354 |
| KEGG:R01930 | GO:trithionate hydrolase activity | GO:0047401 |
| KEGG:R01931 | GO:thiosulfate sulfurtransferase activity | GO:0004792 |
| KEGG:R01932 | GO:2-oxoadipate reductase activity | GO:0047550 |
| KEGG:R01939 | GO:2-aminoadipate transaminase activity | GO:0047536 |
| KEGG:R01944 | GO:putrescine N-hydroxycinnamoyltransferase activity | GO:0047174 |
| KEGG:R01946 | GO:isocitrate O-dihydroxycinnamoyltransferase activity | GO:0047168 |
| KEGG:R01951 | GO:fucose-1-phosphate guanylyltransferase activity | GO:0047341 |
| KEGG:R01957 | GO:kynurenine-glyoxylate transaminase activity | GO:0047315 |
| KEGG:R01960 | GO:kynurenine 3-monooxygenase activity | GO:0004502 |
| KEGG:R01967 | GO:deoxyguanosine kinase activity | GO:0004138 |
| KEGG:R01971 | GO:indolelactate dehydrogenase activity | GO:0047722 |
| KEGG:R01978 | GO:hydroxymethylglutaryl-CoA synthase activity | GO:0004421 |
| KEGG:R01980 | GO:galacturonokinase activity | GO:0047912 |
| KEGG:R01981 | GO:uronate dehydrogenase activity | GO:0050388 |
| KEGG:R01990 | GO:guanidinobutyrase activity | GO:0047971 |
| KEGG:R01993 | GO:dihydroorotase activity | GO:0004151 |
| KEGG:R02003 | GO:geranyltranstransferase activity | GO:0004337 |
| KEGG:R02004 | GO:(-)-endo-fenchol synthase activity | GO:0050437 |
| KEGG:R02006 | GO:sabinene-hydrate synthase activity | GO:0050469 |
| KEGG:R02007 | GO:geranyl-diphosphate cyclase activity | GO:0047926 |
| KEGG:R02009 | GO:myrcene synthase activity | GO:0050551 |
| KEGG:R02016 | GO:thioredoxin-disulfide reductase activity | GO:0004791 |
| KEGG:R02021 | GO:phosphoadenylyl-sulfate reductase (thioredoxin) activity | GO:0004604 |
| KEGG:R02035 | GO:6-phosphogluconolactonase activity | GO:0017057 |
| KEGG:R02036 | GO:phosphogluconate dehydratase activity | GO:0004456 |
| KEGG:R02037 | GO:phosphoethanolamine N-methyltransferase activity | GO:0000234 |
| KEGG:R02050 | GO:(R)-3-amino-2-methylpropionate-pyruvate transaminase activity | GO:0047305 |
| KEGG:R02055 | GO:phosphatidylserine decarboxylase activity | GO:0004609 |
| KEGG:R02056 | GO:phosphatidylethanolamine N-methyltransferase activity | GO:0004608 |
| KEGG:R02058 | GO:glucosamine 6-phosphate N-acetyltransferase activity | GO:0004343 |
| KEGG:R02060 | GO:phosphoglucosamine mutase activity | GO:0008966 |
| KEGG:R02061 | GO:farnesyltranstransferase activity | GO:0004311 |
| KEGG:R02064 | GO:casbene synthase activity | GO:0050449 |
| KEGG:R02068 | GO:ent-copalyl diphosphate synthase activity | GO:0009905 |
| KEGG:R02077 | GO:dihydroxyphenylalanine transaminase activity | GO:0047309 |
| KEGG:R02081 | GO:hydroxymethylglutaryl-CoA reductase activity | GO:0042282 |
| KEGG:R02082 | GO:hydroxymethylglutaryl-CoA reductase (NADPH) activity | GO:0004420 |
| KEGG:R02083 | GO:hydroxymethylglutaryl-CoA hydrolase activity | GO:0047994 |
| KEGG:R02085 | GO:methylglutaconyl-CoA hydratase activity | GO:0004490 |
| KEGG:R02089 | GO:deoxyadenosine kinase activity | GO:0004136 |
| KEGG:R02100 | GO:dUTP diphosphatase activity | GO:0004170 |
| KEGG:R02101 | GO:thymidylate synthase activity | GO:0004799 |
| KEGG:R02118 | GO:trans-zeatin O-beta-D-glucosyltransferase activity | GO:0050403 |
| KEGG:R02119 | GO:zeatin O-beta-D-xylosyltransferase activity | GO:0050404 |
| KEGG:R02126 | GO:retinal isomerase activity | GO:0004744 |
| KEGG:R02133 | GO:thiaminase activity | GO:0050334 |
| KEGG:R02134 | GO:thiamine kinase activity | GO:0019165 |
| KEGG:R02136 | GO:xylitol kinase activity | GO:0050400 |
| KEGG:R02144 | GO:carnosine N-methyltransferase activity | GO:0030735 |
| KEGG:R02150 | GO:histamine oxidase activity | GO:0052598 |
| KEGG:R02155 | GO:histamine N-methyltransferase activity | GO:0046539 |
| KEGG:R02156 | GO:quercetin 2,3-dioxygenase activity | GO:0008127 |
| KEGG:R02159 | GO:flavonol 3-sulfotransferase activity | GO:0047894 |
| KEGG:R02164 | GO:succinate dehydrogenase (ubiquinone) activity | GO:0008177 |
| KEGG:R02167 | GO:mannitol-1-phosphatase activity | GO:0050084 |
| KEGG:R02173 | GO:tryptamine:oxygen oxidoreductase (deaminating) activity | GO:0052593 |
| KEGG:R02177 | GO:(-)-menthol dehydrogenase activity | GO:0047504 |
| KEGG:R02178 | GO:(-)-menthol monooxygenase activity | GO:0047505 |
| KEGG:R02179 | GO:monoterpenol beta-glucosyltransferase activity | GO:0047219 |
| KEGG:R02181 | GO:N-acetylgalactosamine 4-sulfate 6-O-sulfotransferase activity | GO:0050659 |
| KEGG:R02182 | GO:N-acyl-D-aspartate deacylase activity | GO:0047422 |
| KEGG:R02199 | GO:L-isoleucine transaminase activity | GO:0052656 |
| KEGG:R02200 | GO:valine-3-methyl-2-oxovalerate transaminase activity | GO:0047301 |
| KEGG:R02204 | GO:L-pipecolate oxidase activity | GO:0050031 |
| KEGG:R02212 | GO:progesterone monooxygenase activity | GO:0050214 |
| KEGG:R02214 | GO:progesterone 11-alpha-monooxygenase activity | GO:0050212 |
| KEGG:R02225 | GO:aminoglycoside 6-kinase activity | GO:0050300 |
| KEGG:R02226 | GO:aminoglycoside 3''-adenylyltransferase activity | GO:0009012 |
| KEGG:R02227 | GO:streptomycin 3''-kinase activity | GO:0050299 |
| KEGG:R02228 | GO:streptomycin-6-phosphatase activity | GO:0050301 |
| KEGG:R02232 | GO:(1-hydroxycyclohexan-1-yl)acetyl-CoA lyase activity | GO:0047446 |
| KEGG:R02234 | GO:cyclohexanone dehydrogenase activity | GO:0047797 |
| KEGG:R02238 | GO:formate-dihydrofolate ligase activity | GO:0047897 |
| KEGG:R02243 | GO:aconitate decarboxylase activity | GO:0047613 |
| KEGG:R02244 | GO:aconitate delta-isomerase activity | GO:0047614 |
| KEGG:R02245 | GO:mevalonate kinase activity | GO:0004496 |
| KEGG:R02246 | GO:mevaldate reductase (NAD+) activity | GO:0019727 |
| KEGG:R02247 | GO:mevaldate reductase (NADPH) activity | GO:0019726 |
| KEGG:R02254 | GO:trans-cinnamate 2-monooxygenase activity | GO:0050344 |
| KEGG:R02256 | GO:cinnamate beta-D-glucosyltransferase activity | GO:0050412 |
| KEGG:R02258 | GO:S-lactaldehyde reductase activity | GO:0052661 |
| KEGG:R02259 | GO:lactaldehyde reductase (NADPH) activity | GO:0050039 |
| KEGG:R02262 | GO:L-fuculose-phosphate aldolase activity | GO:0008738 |
| KEGG:R02264 | GO:prostaglandin H2 endoperoxidase reductase activity | GO:0036130 |
| KEGG:R02265 | GO:prostaglandin-E synthase activity | GO:0050220 |
| KEGG:R02266 | GO:prostaglandin-D synthase activity | GO:0004667 |
| KEGG:R02267 | GO:prostaglandin-I synthase activity | GO:0008116 |
| KEGG:R02268 | GO:thromboxane-A synthase activity | GO:0004796 |
| KEGG:R02271 | GO:aminolevulinate transaminase activity | GO:0047665 |
| KEGG:R02272 | GO:glutamate-1-semialdehyde 2,1-aminomutase activity | GO:0042286 |
| KEGG:R02274 | GO:5-aminovalerate transaminase activity | GO:0047589 |
| KEGG:R02277 | GO:secondary-alcohol oxidase activity | GO:0033714 |
| KEGG:R02278 | GO:2-dehydro-3-deoxy-L-arabinonate dehydratase activity | GO:0047449 |
| KEGG:R02279 | GO:5-dehydro-4-deoxyglucarate dehydratase activity | GO:0047448 |
| KEGG:R02280 | GO:1-pyrroline-4-hydroxy-2-carboxylate deaminase activity | GO:0047425 |
| KEGG:R02282 | GO:glutamate N-acetyltransferase activity | GO:0004358 |
| KEGG:R02283 | GO:N2-acetyl-L-ornithine:2-oxoglutarate 5-aminotransferase activity | GO:0003992 |
| KEGG:R02284 | GO:carboxymethylhydantoinase activity | GO:0047771 |
| KEGG:R02285 | GO:formimidoylglutamase activity | GO:0050415 |
| KEGG:R02288 | GO:imidazolonepropionase activity | GO:0050480 |
| KEGG:R02291 | GO:aspartate-semialdehyde dehydrogenase activity | GO:0004073 |
| KEGG:R02292 | GO:4-hydroxy-tetrahydrodipicolinate synthase | GO:0008840 |
| KEGG:R02293 | GO:diaminobutyrate-pyruvate transaminase activity | GO:0047307 |
| KEGG:R02301 | GO:5-formyltetrahydrofolate cyclo-ligase activity | GO:0030272 |
| KEGG:R02302 | GO:formimidoyltetrahydrofolate cyclodeaminase activity | GO:0030412 |
| KEGG:R02303 | GO:farnesyl diphosphate kinase activity | GO:0047887 |
| KEGG:R02305 | GO:pentalenene synthase activity | GO:0050467 |
| KEGG:R02306 | GO:trichodiene synthase activity | GO:0045482 |
| KEGG:R02313 | GO:saccharopine dehydrogenase (NAD+, L-glutamate-forming) activity | GO:0047131 |
| KEGG:R02315 | GO:saccharopine dehydrogenase (NADP+, L-glutamate-forming) activity | GO:0004755 |
| KEGG:R02318 | GO:isocitrate epimerase activity | GO:0047755 |
| KEGG:R02324 | GO:ribosylnicotinamide kinase activity | GO:0050262 |
| KEGG:R02328 | GO:glucose-1-phosphate thymidylyltransferase activity | GO:0008879 |
| KEGG:R02329 | GO:galactose-1-phosphate thymidylyltransferase activity | GO:0047342 |
| KEGG:R02338 | GO:indole 2,3-dioxygenase activity | GO:0047719 |
| KEGG:R02340 | GO:indole-3-glycerol-phosphate lyase activity | GO:0033984 |
| KEGG:R02343 | GO:acetoin dehydrogenase activity | GO:0019152 |
| KEGG:R02345 | GO:acetoin-ribose-5-phosphate transaldolase activity | GO:0047156 |
| KEGG:R02359 | GO:urethanase activity | GO:0050387 |
| KEGG:R02368 | GO:all-trans-retinyl-palmitate hydrolase, all-trans-retinol forming activity | GO:0047376 |
| KEGG:R02368 | GO:retinyl-palmitate esterase activity | GO:0050253 |
| KEGG:R02369 | GO:retinol isomerase activity | GO:0050251 |
| KEGG:R02373 | GO:ecdysone oxidase activity | GO:0047875 |
| KEGG:R02374 | GO:ecdysone 20-monooxygenase activity | GO:0004501 |
| KEGG:R02375 | GO:ecdysone O-acyltransferase activity | GO:0004173 |
| KEGG:R02376 | GO:propanediol dehydratase activity | GO:0050215 |
| KEGG:R02378 | GO:pyrazolylalanine synthase activity | GO:0050234 |
| KEGG:R02381 | GO:sinapine esterase activity | GO:0050285 |
| KEGG:R02384 | GO:tyramine N-methyltransferase activity | GO:0030738 |
| KEGG:R02394 | GO:bilirubin oxidase activity | GO:0047705 |
| KEGG:R02395 | GO:carnitine 3-dehydrogenase activity | GO:0047728 |
| KEGG:R02396 | GO:carnitine O-acetyltransferase activity | GO:0004092 |
| KEGG:R02397 | GO:gamma-butyrobetaine dioxygenase activity | GO:0008336 |
| KEGG:R02398 | GO:carnitine decarboxylase activity | GO:0047729 |
| KEGG:R02402 | GO:glutarate-CoA ligase activity | GO:0047948 |
| KEGG:R02408 | GO:L-cystine L-cysteine-lyase (deaminating) | GO:0044540 |
| KEGG:R02412 | GO:shikimate kinase activity | GO:0004765 |
| KEGG:R02417 | GO:questin monooxygenase activity | GO:0050246 |
| KEGG:R02419 | GO:maleate hydratase activity | GO:0050075 |
| KEGG:R02422 | GO:allantoicase activity | GO:0004037 |
| KEGG:R02426 | GO:CDP-glucose 4,6-dehydratase activity | GO:0047733 |
| KEGG:R02429 | GO:xylonate dehydratase activity | GO:0050401 |
| KEGG:R02430 | GO:erythritol kinase activity | GO:0047878 |
| KEGG:R02436 | GO:quercitrinase activity | GO:0050245 |
| KEGG:R02437 | GO:L-rhamnose isomerase activity | GO:0008740 |
| KEGG:R02441 | GO:L-arabinitol 2-dehydrogenase activity | GO:0047002 |
| KEGG:R02449 | GO:benzoyl-CoA 3-monooxygenase activity | GO:0047090 |
| KEGG:R02450 | GO:phenylglyoxylate dehydrogenase (acylating) activity | GO:0047110 |
| KEGG:R02451 | GO:benzoyl-CoA reductase activity | GO:0018522 |
| KEGG:R02452 | GO:glycine N-benzoyltransferase activity | GO:0047962 |
| KEGG:R02453 | GO:anthranilate N-benzoyltransferase activity | GO:0047672 |
| KEGG:R02454 | GO:fructuronate reductase activity | GO:0008866 |
| KEGG:R02458 | GO:D-arginase activity | GO:0047817 |
| KEGG:R02461 | GO:D-ornithine 4,5-aminomutase activity | GO:0047831 |
| KEGG:R02463 | GO:2-hexadecenal reductase activity | GO:0047543 |
| KEGG:R02467 | GO:cysteamine dioxygenase activity | GO:0047800 |
| KEGG:R02469 | GO:(S)-limonene 3-monooxygenase activity | GO:0018674 |
| KEGG:R02470 | GO:(S)-limonene 7-monooxygenase activity | GO:0018676 |
| KEGG:R02471 | GO:pantoate 4-dehydrogenase activity | GO:0050166 |
| KEGG:R02472 | GO:2-dehydropantoate 2-reductase activity | GO:0008677 |
| KEGG:R02474 | GO:pantothenase activity | GO:0004593 |
| KEGG:R02481 | GO:D-tryptophan N-acetyltransferase activity | GO:0047835 |
| KEGG:R02482 | GO:D-tryptophan N-malonyltransferase activity | GO:0047836 |
| KEGG:R02486 | GO:deoxyuridine 1'-dioxygenase activity | GO:0047079 |
| KEGG:R02489 | GO:gentisate decarboxylase activity | GO:0047923 |
| KEGG:R02504 | GO:trimetaphosphatase activity | GO:0050351 |
| KEGG:R02505 | GO:pinosylvin synthase activity | GO:0050198 |
| KEGG:R02509 | GO:N,N-dimethylformamidase activity | GO:0050116 |
| KEGG:R02512 | GO:trimethylamine-oxide aldolase activity | GO:0050352 |
| KEGG:R02519 | GO:homogentisate 1,2-dioxygenase activity | GO:0004411 |
| KEGG:R02522 | GO:L-arabinonate dehydratase activity | GO:0050020 |
| KEGG:R02526 | GO:L-arabinonolactonase activity | GO:0050021 |
| KEGG:R02529 | GO:aminoacetone:oxygen oxidoreductase(deaminating) activity | GO:0052594 |
| KEGG:R02530 | GO:lactoylglutathione lyase activity | GO:0004462 |
| KEGG:R02535 | GO:dopamine beta-monooxygenase activity | GO:0004500 |
| KEGG:R02539 | GO:phenylacetate-CoA ligase activity | GO:0047475 |
| KEGG:R02541 | GO:sphingomyelin phosphodiesterase activity | GO:0004767 |
| KEGG:R02542 | GO:sphingomyelin phosphodiesterase D activity | GO:0050290 |
| KEGG:R02544 | GO:meso-tartrate dehydrogenase activity | GO:0050092 |
| KEGG:R02546 | GO:tartrate epimerase activity | GO:0050320 |
| KEGG:R02547 | GO:trans-epoxysuccinate hydrolase activity | GO:0050345 |
| KEGG:R02548 | GO:(+)-neomenthol dehydrogenase activity | GO:0047501 |
| KEGG:R02551 | GO:N-benzyloxycarbonylglycine hydrolase activity | GO:0050125 |
| KEGG:R02552 | GO:N(alpha)-benzyloxycarbonylleucine hydrolase activity | GO:0047413 |
| KEGG:R02553 | GO:cyclopentanol dehydrogenase activity | GO:0055041 |
| KEGG:R02554 | GO:cyclopentanone monooxygenase activity | GO:0047799 |
| KEGG:R02555 | GO:tagaturonate reductase activity | GO:0009026 |
| KEGG:R02558 | GO:prunasin beta-glucosidase activity | GO:0050224 |
| KEGG:R02563 | GO:serine-phosphoethanolamine synthase activity | GO:0047494 |
| KEGG:R02564 | GO:cyclamate sulfohydrolase activity | GO:0018789 |
| KEGG:R02572 | GO:thioether S-methyltransferase activity | GO:0004790 |
| KEGG:R02573 | GO:trimethylsulfonium-tetrahydrofolate N-methyltransferase activity | GO:0047147 |
| KEGG:R02574 | GO:dimethylpropiothetin dethiomethylase activity | GO:0047869 |
| KEGG:R02575 | GO:guanidinoacetate kinase activity | GO:0047973 |
| KEGG:R02587 | GO:2-deoxyglucose-6-phosphatase activity | GO:0003850 |
| KEGG:R02589 | GO:3-hydroxybenzoate 6-monooxygenase activity | GO:0018669 |
| KEGG:R02591 | GO:glycerophosphocholine cholinephosphodiesterase activity | GO:0047390 |
| KEGG:R02605 | GO:L-2-amino-4-chloropent-4-enoate dehydrochlorinase activity | GO:0047460 |
| KEGG:R02610 | GO:cholestenone 5-alpha-reductase activity | GO:0047751 |
| KEGG:R02613 | GO:phenethylamine:oxygen oxidoreductase (deaminating) activity | GO:0052596 |
| KEGG:R02615 | GO:styrene-oxide isomerase activity | GO:0018846 |
| KEGG:R02632 | GO:quercetin-3-sulfate 3'-sulfotransferase activity | GO:0047365 |
| KEGG:R02633 | GO:quercetin-3-sulfate 4'-sulfotransferase activity | GO:0047366 |
| KEGG:R02636 | GO:UDP-galacturonate decarboxylase activity | GO:0050374 |
| KEGG:R02640 | GO:L-gulonate 3-dehydrogenase activity | GO:0050104 |
| KEGG:R02646 | GO:urate-ribonucleotide phosphorylase activity | GO:0050384 |
| KEGG:R02648 | GO:glycerol-1,2-cyclic-phosphate 2-phosphodiesterase activity | GO:0047393 |
| KEGG:R02651 | GO:N-acylmannosamine 1-dehydrogenase activity | GO:0050123 |
| KEGG:R02656 | GO:gentisate 1,2-dioxygenase activity | GO:0047922 |
| KEGG:R02658 | GO:dehydrogluconokinase activity | GO:0047841 |
| KEGG:R02665 | GO:3-hydroxyanthranilate 3,4-dioxygenase activity | GO:0000334 |
| KEGG:R02666 | GO:3-hydroxyanthranilate oxidase activity | GO:0047561 |
| KEGG:R02667 | GO:3-hydroxyanthranilate 4-C-methyltransferase activity | GO:0030767 |
| KEGG:R02673 | GO:4-hydroxymandelate oxidase activity | GO:0047579 |
| KEGG:R02679 | GO:indole-3-acetaldehyde reductase (NADH) activity | GO:0047018 |
| KEGG:R02680 | GO:indole-3-acetaldehyde reductase (NADPH) activity | GO:0047019 |
| KEGG:R02681 | GO:indole-3-acetaldehyde oxidase activity | GO:0050302 |
| KEGG:R02691 | GO:1,2-diacylglycerol 3-beta-galactosyltransferase activity | GO:0046509 |
| KEGG:R02698 | GO:4-hydroxyphenylacetate,NADH:oxygen oxidoreductase (3-hydroxylating) activity | GO:0018660 |
| KEGG:R02708 | GO:hydroxyphenylacetonitrile 2-monooxygenase activity | GO:0047085 |
| KEGG:R02709 | GO:hydroxymandelonitrile glucosyltransferase activity | GO:0047239 |
| KEGG:R02711 | GO:4-methyleneglutamate-ammonia ligase activity | GO:0047581 |
| KEGG:R02712 | GO:4-methyleneglutaminase activity | GO:0047582 |
| KEGG:R02714 | GO:D-arabinonolactonase activity | GO:0047815 |
| KEGG:R02715 | GO:D-arabinono-1,4-lactone oxidase activity | GO:0003885 |
| KEGG:R02720 | GO:XTP diphosphatase activity | GO:0036222 |
| KEGG:R02722 | GO:tryptophan synthase activity | GO:0004834 |
| KEGG:R02728 | GO:beta-phosphoglucomutase activity | GO:0008801 |
| KEGG:R02729 | GO:glycine formimidoyltransferase activity | GO:0030408 |
| KEGG:R02730 | GO:fructose-2,6-bisphosphate 6-phosphatase activity | GO:0047386 |
| KEGG:R02732 | GO:6-phosphofructo-2-kinase activity | GO:0003873 |
| KEGG:R02733 | GO:N-acetyldiaminopimelate deacetylase activity | GO:0050118 |
| KEGG:R02734 | GO:succinyl-diaminopimelate desuccinylase activity | GO:0009014 |
| KEGG:R02735 | GO:diaminopimelate epimerase activity | GO:0008837 |
| KEGG:R02741 | GO:homoglutathione synthase activity | GO:0047983 |
| KEGG:R02742 | GO:bis-gamma-glutamylcystine reductase activity | GO:0047135 |
| KEGG:R02755 | GO:diaminopimelate dehydrogenase activity | GO:0047850 |
| KEGG:R02764 | GO:(S)-methylmalonyl-CoA hydrolase activity | GO:0047511 |
| KEGG:R02765 | GO:methylmalonyl-CoA epimerase activity | GO:0004493 |
| KEGG:R02776 | GO:dTDP-6-deoxy-L-talose 4-dehydrogenase activity | GO:0047003 |
| KEGG:R02777 | GO:dTDP-4-dehydrorhamnose reductase activity | GO:0008831 |
| KEGG:R02781 | GO:glutamine-scyllo-inositol transaminase activity | GO:0047310 |
| KEGG:R02782 | GO:myo-inosose-2 dehydratase activity | GO:0050114 |
| KEGG:R02783 | GO:UDP-N-acetylmuramoylalanine-D-glutamate ligase activity | GO:0008764 |
| KEGG:R02786 | GO:UDP-N-acetylmuramoyl-L-alanyl-D-glutamate-L-lysine ligase activity | GO:0047482 |
| KEGG:R02788 | GO:UDP-N-acetylmuramoylalanyl-D-glutamate-2,6-diaminopimelate ligase activity | GO:0008765 |
| KEGG:R02792 | GO:cholate 7-alpha-dehydrogenase activity | GO:0008709 |
| KEGG:R02793 | GO:cholate 12-alpha dehydrogenase activity | GO:0047013 |
| KEGG:R02799 | GO:prostaglandin D2 11-ketoreductase activity | GO:0036131 |
| KEGG:R02804 | GO:nitrous-oxide reductase activity | GO:0050304 |
| KEGG:R02807 | GO:mercury (II) reductase activity | GO:0016152 |
| KEGG:R02813 | GO:oleate hydratase activity | GO:0050151 |
| KEGG:R02817 | GO:serine-ethanolaminephosphate phosphodiesterase activity | GO:0047387 |
| KEGG:R02820 | GO:alditol:NADP+ 1-oxidoreductase activity | GO:0004032 |
| KEGG:R02821 | GO:betaine-homocysteine S-methyltransferase activity | GO:0047150 |
| KEGG:R02830 | GO:orcinol 2-monooxygenase activity | GO:0018661 |
| KEGG:R02831 | GO:orsellinate decarboxylase activity | GO:0050159 |
| KEGG:R02832 | GO:tropine dehydrogenase activity | GO:0050356 |
| KEGG:R02837 | GO:cortisol O-acetyltransferase activity | GO:0047784 |
| KEGG:R02839 | GO:cortisol sulfotransferase activity | GO:0047785 |
| KEGG:R02852 | GO:D-lysine 5,6-aminomutase activity | GO:0047826 |
| KEGG:R02853 | GO:D-phosphoserine phosphatase activity | GO:0036425 |
| KEGG:R02855 | GO:diacetyl reductase ((R)-acetoin forming) activity | GO:0052587 |
| KEGG:R02858 | GO:macrocin O-methyltransferase activity | GO:0030769 |
| KEGG:R02859 | GO:demethylmacrocin O-methyltransferase activity | GO:0030770 |
| KEGG:R02862 | GO:pyridine N-methyltransferase activity | GO:0030760 |
| KEGG:R02863 | GO:thiamine pyridinylase activity | GO:0050332 |
| KEGG:R02864 | GO:sirohydrochlorin ferrochelatase activity | GO:0051266 |
| KEGG:R02866 | GO:sorbitol-6-phosphatase activity | GO:0050286 |
| KEGG:R02874 | GO:squalene monooxygenase activity | GO:0004506 |
| KEGG:R02877 | GO:vanillyl-alcohol oxidase activity | GO:0018465 |
| KEGG:R02878 | GO:octanol dehydrogenase activity | GO:0004552 |
| KEGG:R02892 | GO:cortisone alpha-reductase activity | GO:0047786 |
| KEGG:R02899 | GO:glucarate O-hydroxycinnamoyltransferase activity | GO:0047170 |
| KEGG:R02901 | GO:phloretin hydrolase activity | GO:0050180 |
| KEGG:R02907 | GO:scytalone dehydratase activity | GO:0030411 |
| KEGG:R02914 | GO:urocanate hydratase activity | GO:0016153 |
| KEGG:R02918 | GO:tyrosine-tRNA ligase activity | GO:0004831 |
| KEGG:R02921 | GO:D-ribitol-5-phosphate cytidylyltransferase activity | GO:0047349 |
| KEGG:R02927 | GO:tagatose kinase activity | GO:0050317 |
| KEGG:R02928 | GO:galactitol 2-dehydrogenase activity | GO:0047713 |
| KEGG:R02929 | GO:glutamate-ethylamine ligase activity | GO:0047942 |
| KEGG:R02930 | GO:theanine hydrolase activity | GO:0050330 |
| KEGG:R02937 | GO:oxamate carbamoyltransferase activity | GO:0050205 |
| KEGG:R02938 | GO:pentanamidase activity | GO:0050168 |
| KEGG:R02942 | GO:acetylsalicylate deacetylase activity | GO:0047610 |
| KEGG:R02944 | GO:(+)-borneol dehydrogenase activity | GO:0047500 |
| KEGG:R02945 | GO:(-)-borneol dehydrogenase activity | GO:0047503 |
| KEGG:R02949 | GO:acetoin racemase activity | GO:0047604 |
| KEGG:R02954 | GO:biochanin-A reductase activity | GO:0047706 |
| KEGG:R02965 | GO:hydrogen:quinone oxidoreductase activity | GO:0047067 |
| KEGG:R02969 | GO:hydroxymalonate dehydrogenase activity | GO:0047993 |
| KEGG:R02970 | GO:alanine-oxomalonate transaminase activity | GO:0047308 |
| KEGG:R02972 | GO:pantothenoylcysteine decarboxylase activity | GO:0050167 |
| KEGG:R02973 | GO:pantetheine hydrolase activity | GO:0017159 |
| KEGG:R02978 | GO:3-dehydrosphinganine reductase activity | GO:0047560 |
| KEGG:R02982 | GO:8-hydroxyfuranocoumarin 8-O-methyltransferase activity | GO:0030753 |
| KEGG:R02985 | GO:amygdalin beta-glucosidase activity | GO:0047668 |
| KEGG:R02986 | GO:2-furoate-CoA ligase activity | GO:0047541 |
| KEGG:R02987 | GO:2-furoyl-CoA dehydrogenase activity | GO:0047542 |
| KEGG:R02992 | GO:4-pyridoxolactonase activity | GO:0047585 |
| KEGG:R02994 | GO:6-endo-hydroxycineole dehydrogenase activity | GO:0045481 |
| KEGG:R02995 | GO:6-oxocineole dehydrogenase activity | GO:0047597 |
| KEGG:R02997 | GO:chlorogenate hydrolase activity | GO:0047745 |
| KEGG:R02998 | GO:chlorogenate-glucarate O-hydroxycinnamoyltransferase activity | GO:0047204 |
| KEGG:R02999 | GO:cyanocobalamin reductase (cyanide-eliminating) activity | GO:0033787 |
| KEGG:R03001 | GO:D-methionine-pyruvate transaminase activity | GO:0047306 |
| KEGG:R03013 | GO:histidinol-phosphatase activity | GO:0004401 |
| KEGG:R03015 | GO:formylmethanofuran dehydrogenase activity | GO:0018493 |
| KEGG:R03021 | GO:(R)-2-hydroxy-fatty acid dehydrogenase activity | GO:0047049 |
| KEGG:R03022 | GO:(S)-2-hydroxy-fatty acid dehydrogenase activity | GO:0047050 |
| KEGG:R03023 | GO:4-nitrophenol 2-monooxygenase activity | GO:0018601 |
| KEGG:R03025 | GO:coenzyme F420 hydrogenase activity | GO:0050454 |
| KEGG:R03027 | GO:3-hydroxybutyryl-CoA dehydratase activity | GO:0003859 |
| KEGG:R03028 | GO:glutaconyl-CoA decarboxylase activity | GO:0018801 |
| KEGG:R03030 | GO:3-aminobutyryl-CoA ammonia-lyase activity | GO:0047459 |
| KEGG:R03031 | GO:vinylacetyl-CoA delta-isomerase activity | GO:0050393 |
| KEGG:R03032 | GO:arabinonate dehydratase activity | GO:0047675 |
| KEGG:R03033 | GO:galactonate dehydratase activity | GO:0008869 |
| KEGG:R03035 | GO:pantetheine-phosphate adenylyltransferase activity | GO:0004595 |
| KEGG:R03037 | GO:isochorismatase activity | GO:0008908 |
| KEGG:R03040 | GO:3-propylmalate synthase activity | GO:0050442 |
| KEGG:R03043 | GO:pregnan-21-ol dehydrogenase (NAD+) activity | GO:0047007 |
| KEGG:R03044 | GO:pregnan-21-ol dehydrogenase (NADP+) activity | GO:0047008 |
| KEGG:R03049 | GO:11-cis-retinyl-palmitate hydrolase activity | GO:0047520 |
| KEGG:R03056 | GO:aminoglycoside 2'-N-acetyltransferase activity | GO:0047921 |
| KEGG:R03057 | GO:leukotriene-A4 hydrolase activity | GO:0004463 |
| KEGG:R03059 | GO:leukotriene-C4 synthase activity | GO:0004464 |
| KEGG:R03062 | GO:cephalosporin-C deacetylase activity | GO:0047739 |
| KEGG:R03063 | GO:cephalosporin-C transaminase activity | GO:0047740 |
| KEGG:R03064 | GO:deacetylcephalosporin-C acetyltransferase activity | GO:0033813 |
| KEGG:R03065 | GO:chloramphenicol O-acetyltransferase activity | GO:0008811 |
| KEGG:R03069 | GO:dimethylmaleate hydratase activity | GO:0047868 |
| KEGG:R03070 | GO:methylitaconate delta-isomerase activity | GO:0050100 |
| KEGG:R03072 | GO:isonocardicin synthase activity | GO:0050007 |
| KEGG:R03073 | GO:nocardicin-A epimerase activity | GO:0050143 |
| KEGG:R03075 | GO:sinapoylglucose-choline O-sinapoyltransferase activity | GO:0047202 |
| KEGG:R03080 | GO:R-lactaldehyde reductase activity | GO:0052660 |
| KEGG:R03083 | GO:3-dehydroquinate synthase activity | GO:0003856 |
| KEGG:R03084 | GO:3-dehydroquinate dehydratase activity | GO:0003855 |
| KEGG:R03086 | GO:estradiol 6-beta-monooxygenase activity | GO:0047882 |
| KEGG:R03094 | GO:indole-3-acetate beta-glucosyltransferase activity | GO:0047215 |
| KEGG:R03095 | GO:indoleacetate-lysine synthetase activity | GO:0047721 |
| KEGG:R03114 | GO:carveol dehydrogenase activity | GO:0018459 |
| KEGG:R03127 | GO:dihydroxyfumarate decarboxylase activity | GO:0047858 |
| KEGG:R03133 | GO:zeatin 9-aminocarboxyethyltransferase activity | GO:0050447 |
| KEGG:R03134 | GO:beta-pyrazolylalanine synthase activity | GO:0047458 |
| KEGG:R03136 | GO:polyvinyl alcohol dehydrogenase (cytochrome) activity | GO:0047059 |
| KEGG:R03138 | GO:Renilla-luciferin sulfotransferase activity | GO:0050249 |
| KEGG:R03139 | GO:propane-1,3-diamine oxidase activity | GO:0052600 |
| KEGG:R03145 | GO:pyruvate dehydrogenase (quinone) activity | GO:0052737 |
| KEGG:R03155 | GO:2-dehydropantolactone reductase activity | GO:0036441 |
| KEGG:R03156 | GO:(R)-pantolactone dehydrogenase (flavin) activity | GO:0047060 |
| KEGG:R03161 | GO:fucokinase activity | GO:0050201 |
| KEGG:R03165 | GO:uroporphyrinogen-III synthase activity | GO:0004852 |
| KEGG:R03168 | GO:N6-hydroxylysine O-acetyltransferase activity | GO:0050133 |
| KEGG:R03169 | GO:2-methyl-branched-chain-enoyl-CoA reductase activity | GO:0047119 |
| KEGG:R03177 | GO:gamma-guanidinobutyraldehyde dehydrogenase activity | GO:0047107 |
| KEGG:R03178 | GO:5-guanidino-2-oxopentanoate decarboxylase activity | GO:0047435 |
| KEGG:R03182 | GO:dethiobiotin synthase activity | GO:0004141 |
| KEGG:R03183 | GO:glucuronolactone reductase activity | GO:0047941 |
| KEGG:R03187 | GO:N-methylhydantoinase (ATP-hydrolyzing) activity | GO:0047423 |
| KEGG:R03193 | GO:UDP-N-acetylmuramate-L-alanine ligase activity | GO:0008763 |
| KEGG:R03198 | GO:(R)-dehydropantoate dehydrogenase activity | GO:0047509 |
| KEGG:R03199 | GO:lanosterol synthase activity | GO:0000250 |
| KEGG:R03200 | GO:cycloartenol synthase activity | GO:0016871 |
| KEGG:R03202 | GO:(S)-6-hydroxynicotine oxidase activity | GO:0018531 |
| KEGG:R03206 | GO:2-hydroxypyridine 5-monooxygenase activity | GO:0047546 |
| KEGG:R03207 | GO:diiodotyrosine transaminase activity | GO:0047861 |
| KEGG:R03209 | GO:6-carboxyhexanoate-CoA ligase activity | GO:0042410 |
| KEGG:R03210 | GO:8-amino-7-oxononanoate synthase activity | GO:0008710 |
| KEGG:R03211 | GO:cyclohexane-1,3-dione hydrolase activity | GO:0047796 |
| KEGG:R03212 | GO:3-hydroxycyclohexanone dehydrogenase activity | GO:0047564 |
| KEGG:R03214 | GO:desulfoglucosinolate sulfotransferase activity | GO:0047364 |
| KEGG:R03216 | GO:disulfoglucosamine-6-sulfatase activity | GO:0047871 |
| KEGG:R03218 | GO:procollagen-proline 3-dioxygenase activity | GO:0019797 |
| KEGG:R03219 | GO:procollagen-proline 4-dioxygenase activity | GO:0004656 |
| KEGG:R03220 | GO:coproporphyrinogen oxidase activity | GO:0004109 |
| KEGG:R03222 | GO:oxygen-dependent protoporphyrinogen oxidase activity | GO:0004729 |
| KEGG:R03223 | GO:thiamine-phosphate diphosphorylase activity | GO:0004789 |
| KEGG:R03230 | GO:17-O-deacetylvindoline O-acetyltransferase activity | GO:0047162 |
| KEGG:R03231 | GO:adenosylmethionine-8-amino-7-oxononanoate transaminase activity | GO:0004015 |
| KEGG:R03240 | GO:galactose-6-phosphate isomerase activity | GO:0050044 |
| KEGG:R03241 | GO:L-fuculokinase activity | GO:0008737 |
| KEGG:R03245 | GO:phosphomevalonate kinase activity | GO:0004631 |
| KEGG:R03246 | GO:pyrogallol 1,2-oxygenase activity | GO:0050240 |
| KEGG:R03247 | GO:gallate decarboxylase activity | GO:0018798 |
| KEGG:R03248 | GO:2,5-diaminovalerate transaminase activity | GO:0047531 |
| KEGG:R03251 | GO:kynurenate-7,8-dihydrodiol dehydrogenase activity | GO:0047114 |
| KEGG:R03253 | GO:7,8-dihydroxykynurenate 8,8a-dioxygenase activity | GO:0047069 |
| KEGG:R03254 | GO:3-deoxy-8-phosphooctulonate synthase activity | GO:0008676 |
| KEGG:R03261 | GO:isopiperitenol dehydrogenase activity | GO:0018458 |
| KEGG:R03264 | GO:farnesol dehydrogenase activity | GO:0047886 |
| KEGG:R03265 | GO:farnesol 2-isomerase activity | GO:0047885 |
| KEGG:R03269 | GO:phosphopantothenoylcysteine decarboxylase activity | GO:0004633 |
| KEGG:R03271 | GO:sym-norspermidine synthase activity | GO:0050314 |
| KEGG:R03275 | GO:beta-lysine 5,6-aminomutase activity | GO:0047702 |
| KEGG:R03281 | GO:2-hydroxycyclohexanone 2-monooxygenase activity | GO:0047095 |
| KEGG:R03296 | GO:4-hydroxyproline epimerase activity | GO:0047580 |
| KEGG:R03297 | GO:gallate 1-beta-glucosyltransferase activity | GO:0047913 |
| KEGG:R03303 | GO:3,4-dihydroxyphenylacetate 2,3-dioxygenase activity | GO:0008687 |
| KEGG:R03306 | GO:4-chlorophenylacetate 3,4-dioxygenase activity | GO:0018622 |
| KEGG:R03307 | GO:3-carboxy-cis,cis-muconate cycloisomerase activity | GO:0047472 |
| KEGG:R03308 | GO:carboxy-cis,cis-muconate cyclase activity | GO:0047768 |
| KEGG:R03313 | GO:glutamate-5-semialdehyde dehydrogenase activity | GO:0004350 |
| KEGG:R03315 | GO:pseudouridine kinase activity | GO:0050225 |
| KEGG:R03323 | GO:sinapoylglucose-malate O-sinapoyltransferase activity | GO:0016754 |
| KEGG:R03334 | GO:lysolecithin acylmutase activity | GO:0050070 |
| KEGG:R03341 | GO:4-hydroxyphenylpyruvate decarboxylase activity | GO:0050546 |
| KEGG:R03345 | GO:dimethylaniline-N-oxide aldolase activity | GO:0047864 |
| KEGG:R03348 | GO:nicotinate-nucleotide diphosphorylase (carboxylating) activity | GO:0004514 |
| KEGG:R03349 | GO:L-erythro-3,5-diaminohexanoate dehydrogenase activity | GO:0047124 |
| KEGG:R03350 | GO:3-deoxy-manno-octulosonate-8-phosphatase activity | GO:0019143 |
| KEGG:R03358 | GO:octopamine dehydratase activity | GO:0050202 |
| KEGG:R03359 | GO:synephrine dehydratase activity | GO:0050315 |
| KEGG:R03360 | GO:phosphatidylinositol deacylase activity | GO:0050185 |
| KEGG:R03361 | GO:1-phosphatidylinositol 4-kinase activity | GO:0004430 |
| KEGG:R03362 | GO:1-phosphatidylinositol-3-kinase activity | GO:0016303 |
| KEGG:R03365 | GO:caffeate 3,4-dioxygenase activity | GO:0047762 |
| KEGG:R03369 | GO:melilotate 3-monooxygenase activity | GO:0050091 |
| KEGG:R03375 | GO:N-methylphosphoethanolamine cytidylyltransferase activity | GO:0047353 |
| KEGG:R03378 | GO:hydroxylysine kinase activity | GO:0047992 |
| KEGG:R03384 | GO:scyllo-inosamine 4-kinase activity | GO:0050276 |
| KEGG:R03387 | GO:2-dehydro-3-deoxygalactonokinase activity | GO:0008671 |
| KEGG:R03390 | GO:formylmethanofuran-tetrahydromethanopterin N-formyltransferase activity | GO:0030270 |
| KEGG:R03394 | GO:inositol-1,4,5-trisphosphate 5-phosphatase activity | GO:0052658 |
| KEGG:R03395 | GO:dihydrostreptomycin-6-phosphate 3'-alpha-kinase activity | GO:0047333 |
| KEGG:R03401 | GO:12-oxophytodienoate reductase activity | GO:0016629 |
| KEGG:R03402 | GO:allene-oxide cyclase activity | GO:0046423 |
| KEGG:R03421 | GO:N4-(beta-N-acetylglucosaminyl)-L-asparaginase activity | GO:0003948 |
| KEGG:R03428 | GO:inositol-1,3,4-trisphosphate 5-kinase activity | GO:0052726 |
| KEGG:R03430 | GO:inositol-1,3,4,5-tetrakisphosphate 5-phosphatase activity | GO:0052659 |
| KEGG:R03431 | GO:diiodophenylpyruvate reductase activity | GO:0047860 |
| KEGG:R03433 | GO:inositol-1,4,5-trisphosphate 3-kinase activity | GO:0008440 |
| KEGG:R03435 | GO:phosphatidylinositol phospholipase C activity | GO:0004435 |
| KEGG:R03448 | GO:6-aminohexanoate-cyclic-dimer hydrolase activity | GO:0019874 |
| KEGG:R03451 | GO:trimethyllysine dioxygenase activity | GO:0050353 |
| KEGG:R03452 | GO:3,9-dihydroxypterocarpan 6a-monooxygenase activity | GO:0047082 |
| KEGG:R03454 | GO:alkylacetylglycerophosphatase activity | GO:0047647 |
| KEGG:R03455 | GO:alkylglycerophosphate 2-O-acetyltransferase activity | GO:0047160 |
| KEGG:R03456 | GO:3,7-dimethylquercitin 4'-O-methyltransferase activity | GO:0030758 |
| KEGG:R03457 | GO:imidazoleglycerol-phosphate dehydratase activity | GO:0004424 |
| KEGG:R03458 | GO:5-amino-6-(5-phosphoribosylamino)uracil reductase activity | GO:0008703 |
| KEGG:R03459 | GO:diaminohydroxyphosphoribosylaminopyrimidine deaminase activity | GO:0008835 |
| KEGG:R03460 | GO:3-phosphoshikimate 1-carboxyvinyltransferase activity | GO:0003866 |
| KEGG:R03461 | GO:3-hydroxy-2-methylpyridine-4,5-dicarboxylate 4-decarboxylase activity | GO:0047431 |
| KEGG:R03463 | GO:2-hydroxy-6-oxo-6-phenylhexa-2,4-dienoate reductase activity | GO:0047118 |
| KEGG:R03464 | GO:methenyltetrahydromethanopterin cyclohydrolase activity | GO:0018759 |
| KEGG:R03468 | GO:galactosyldiacylglycerol alpha-2,3-sialyltransferase activity | GO:0047289 |
| KEGG:R03469 | GO:1-phosphatidylinositol-4-phosphate 5-kinase activity | GO:0016308 |
| KEGG:R03470 | GO:4-carboxymuconolactone decarboxylase activity | GO:0047575 |
| KEGG:R03471 | GO:hydroxymethylpyrimidine kinase activity | GO:0008902 |
| KEGG:R03474 | GO:[glutamine synthetase]-adenylyl-L-tyrosine phosphorylase | GO:0047388 |
| KEGG:R03475 | GO:phosphatidylcholine desaturase activity | GO:0050184 |
| KEGG:R03476 | GO:phosphatidylcholine 12-monooxygenase activity | GO:0050183 |
| KEGG:R03477 | GO:scyllo-inosamine-4-phosphate amidinotransferase activity | GO:0015069 |
| KEGG:R03493 | GO:isohexenylglutaconyl-CoA hydratase activity | GO:0050005 |
| KEGG:R03494 | GO:geranoyl-CoA carboxylase activity | GO:0047925 |
| KEGG:R03495 | GO:3alpha,7alpha,12beta-trihydroxy-5beta-cholanate dehydrogenase activity | GO:0047521 |
| KEGG:R03496 | GO:guanidinodeoxy-scyllo-inositol-4-phosphatase activity | GO:0047383 |
| KEGG:R03497 | GO:sequoyitol dehydrogenase activity | GO:0050280 |
| KEGG:R03498 | GO:D-pinitol dehydrogenase activity | GO:0047832 |
| KEGG:R03502 | GO:1D-1-guanidino-3-amino-1,3-dideoxy-scyllo-inositol transaminase activity | GO:0047311 |
| KEGG:R03503 | GO:2-amino-4-hydroxy-6-hydroxymethyldihydropteridine diphosphokinase activity | GO:0003848 |
| KEGG:R03509 | GO:phosphoribosylanthranilate isomerase activity | GO:0004640 |
| KEGG:R03510 | GO:phylloquinone monooxygenase (2,3-epoxidizing) activity | GO:0047097 |
| KEGG:R03517 | GO:chondro-4-sulfatase activity | GO:0033887 |
| KEGG:R03518 | GO:chondro-6-sulfatase activity | GO:0033888 |
| KEGG:R03520 | GO:15-hydroxyprostaglandin-I dehydrogenase (NADP+) activity | GO:0047033 |
| KEGG:R03523 | GO:alkylhalidase activity | GO:0047651 |
| KEGG:R03531 | GO:dITP diphosphatase activity | GO:0035870 |
| KEGG:R03532 | GO:peroxidase activity | GO:0004601 |
| KEGG:R03543 | GO:benzene 1,2-dioxygenase activity | GO:0018619 |
| KEGG:R03548 | GO:o-dihydroxycoumarin 7-O-glucosyltransferase activity | GO:0047208 |
| KEGG:R03558 | GO:salicyl-alcohol beta-D-glucosyltransferase activity | GO:0050274 |
| KEGG:R03559 | GO:toluene dioxygenase activity | GO:0018624 |
| KEGG:R03563 | GO:tropinesterase activity | GO:0050357 |
| KEGG:R03565 | GO:vitexin beta-glucosyltransferase activity | GO:0050395 |
| KEGG:R03567 | GO:6-methylsalicylate decarboxylase activity | GO:0047596 |
| KEGG:R03572 | GO:allyl-alcohol dehydrogenase activity | GO:0047655 |
| KEGG:R03573 | GO:alizarin 2-beta-glucosyltransferase activity | GO:0047644 |
| KEGG:R03575 | GO:chlorate reductase activity | GO:0047143 |
| KEGG:R03577 | GO:apiose 1-reductase activity | GO:0047674 |
| KEGG:R03579 | GO:N-feruloylglycine deacylase activity | GO:0050128 |
| KEGG:R03586 | GO:L-xylose 1-dehydrogenase activity | GO:0050037 |
| KEGG:R03587 | GO:luteolin O-methyltransferase activity | GO:0030744 |
| KEGG:R03589 | GO:luteolin 7-O-glucuronosyltransferase activity | GO:0050064 |
| KEGG:R03594 | GO:scopoletin glucosyltransferase activity | GO:0050275 |
| KEGG:R03595 | GO:selenide, water dikinase activity | GO:0004756 |
| KEGG:R03610 | GO:calcidiol 1-monooxygenase activity | GO:0004498 |
| KEGG:R03612 | GO:cetraxate benzylesterase activity | GO:0047741 |
| KEGG:R03613 | GO:diphthine-ammonia ligase activity | GO:0017178 |
| KEGG:R03614 | GO:ephedrine dehydrogenase activity | GO:0047877 |
| KEGG:R03620 | GO:alkylamidase activity | GO:0047648 |
| KEGG:R03621 | GO:imidazole N-acetyltransferase activity | GO:0047716 |
| KEGG:R03622 | GO:kievitone hydratase activity | GO:0050015 |
| KEGG:R03623 | GO:licodione 2'-O-methyltransferase activity | GO:0030751 |
| KEGG:R03627 | GO:linoleate isomerase activity | GO:0050058 |
| KEGG:R03630 | GO:phthalate 4,5-dioxygenase activity | GO:0018620 |
| KEGG:R03631 | GO:phytanate-CoA ligase activity | GO:0050197 |
| KEGG:R03641 | GO:steroid-lactonase activity | GO:0050293 |
| KEGG:R03642 | GO:vicianin beta-glucosidase activity | GO:0050392 |
| KEGG:R03648 | GO:asparagine-tRNA ligase activity | GO:0004816 |
| KEGG:R03656 | GO:isoleucine-tRNA ligase activity | GO:0004822 |
| KEGG:R03657 | GO:leucine-tRNA ligase activity | GO:0004823 |
| KEGG:R03665 | GO:valine-tRNA ligase activity | GO:0004832 |
| KEGG:R03667 | GO:8-oxocoformycin reductase activity | GO:0047599 |
| KEGG:R03668 | GO:thioethanolamine S-acetyltransferase activity | GO:0050336 |
| KEGG:R03671 | GO:D-fuconate dehydratase activity | GO:0047818 |
| KEGG:R03673 | GO:dopachrome isomerase activity | GO:0004167 |
| KEGG:R03675 | GO:5-alpha-hydroxysteroid dehydratase activity | GO:0047587 |
| KEGG:R03686 | GO:isovitexin beta-glucosyltransferase activity | GO:0050010 |
| KEGG:R03687 | GO:quinaldate 4-oxidoreductase activity | GO:0047122 |
| KEGG:R03688 | GO:L-fuconate dehydratase activity | GO:0050023 |
| KEGG:R03692 | GO:dihydrocoumarin hydrolase activity | GO:0047856 |
| KEGG:R03693 | GO:(S)-2-methylmalate dehydratase activity | GO:0047510 |
| KEGG:R03696 | GO:methylaspartate ammonia-lyase activity | GO:0050096 |
| KEGG:R03702 | GO:triacetate-lactonase activity | GO:0050349 |
| KEGG:R03703 | GO:raucaffricine beta-glucosidase activity | GO:0050247 |
| KEGG:R03704 | GO:methylenetetrahydrofolate-tRNA-(uracil-5-)-methyltransferase (FADH2-oxidizing) activity | GO:0047151 |
| KEGG:R03707 | GO:(S,S)-butanediol dehydrogenase activity | GO:0047512 |
| KEGG:R03709 | GO:2-coumarate reductase activity | GO:0047788 |
| KEGG:R03710 | GO:2-coumarate O-beta-glucosyltransferase activity | GO:0047212 |
| KEGG:R03712 | GO:albendazole monooxygenase activity | GO:0047638 |
| KEGG:R03716 | GO:chlordecone reductase activity | GO:0047743 |
| KEGG:R03721 | GO:columbamine O-methyltransferase activity | GO:0030778 |
| KEGG:R03723 | GO:fucosterol-epoxide lyase activity | GO:0047906 |
| KEGG:R03726 | GO:discadenine synthase activity | GO:0047870 |
| KEGG:R03727 | GO:galactarate O-hydroxycinnamoyltransferase activity | GO:0047169 |
| KEGG:R03731 | GO:isoorientin 3'-O-methyltransferase activity | GO:0030756 |
| KEGG:R03732 | GO:opine dehydrogenase activity | GO:0047129 |
| KEGG:R03733 | GO:L-threonate 3-dehydrogenase activity | GO:0050036 |
| KEGG:R03735 | GO:decylcitrate synthase activity | GO:0050457 |
| KEGG:R03736 | GO:rifamycin-B oxidase activity | GO:0050264 |
| KEGG:R03737 | GO:6-beta-hydroxyhyoscyamine epoxidase activity | GO:0047594 |
| KEGG:R03738 | GO:strictosidine synthase activity | GO:0016844 |
| KEGG:R03739 | GO:stipitatonate decarboxylase activity | GO:0050296 |
| KEGG:R03742 | GO:protoaphin-aglucone dehydratase (cyclizing) activity | GO:0047452 |
| KEGG:R03745 | GO:(+)-sabinol dehydrogenase activity | GO:0047502 |
| KEGG:R03746 | GO:iodophenol O-methyltransferase activity | GO:0030737 |
| KEGG:R03747 | GO:3-oxolaurate decarboxylase activity | GO:0050410 |
| KEGG:R03759 | GO:(R)-aminopropanol dehydrogenase activity | GO:0019147 |
| KEGG:R03761 | GO:asparagusate reductase activity | GO:0050445 |
| KEGG:R03766 | GO:hamamelose kinase activity | GO:0047976 |
| KEGG:R03768 | GO:heteroglycan 2-alpha-mannosyltransferase activity | GO:0052819 |
| KEGG:R03769 | GO:heteroglycan 3-alpha-mannosyltransferase activity | GO:0052818 |
| KEGG:R03770 | GO:hygromycin-B 7''-O-phosphotransferase activity | GO:0008904 |
| KEGG:R03772 | GO:L-rhamnono-1,4-lactonase activity | GO:0050033 |
| KEGG:R03774 | GO:L-rhamnonate dehydratase activity | GO:0050032 |
| KEGG:R03775 | GO:cycloeucalenol cycloisomerase activity | GO:0047793 |
| KEGG:R03779 | GO:carnitine O-octanoyltransferase activity | GO:0008458 |
| KEGG:R03780 | GO:macrolide 2'-kinase activity | GO:0050073 |
| KEGG:R03781 | GO:beta-diketone hydrolase activity | GO:0047699 |
| KEGG:R03782 | GO:isopiperitenone delta-isomerase activity | GO:0050008 |
| KEGG:R03785 | GO:taurocyamine kinase activity | GO:0050324 |
| KEGG:R03787 | GO:xanthommatin reductase activity | GO:0050399 |
| KEGG:R03794 | GO:mandelate 4-monooxygenase activity | GO:0050481 |
| KEGG:R03796 | GO:oximinotransferase activity | GO:0050206 |
| KEGG:R03799 | GO:aspulvinone dimethylallyltransferase activity | GO:0047691 |
| KEGG:R03803 | GO:deoxylimonate A-ring-lactonase activity | GO:0047845 |
| KEGG:R03810 | GO:2,5-dioxopiperazine hydrolase activity | GO:0047532 |
| KEGG:R03812 | GO:hyoscyamine (6S)-dioxygenase activity | GO:0047998 |
| KEGG:R03817 | GO:plastoquinol--plastocyanin reductase activity | GO:0009496 |
| KEGG:R03820 | GO:strictosidine beta-glucosidase activity | GO:0050422 |
| KEGG:R03831 | GO:reticuline oxidase activity | GO:0050468 |
| KEGG:R03834 | GO:(S)-cheilanthifoline synthase activity | GO:0047053 |
| KEGG:R03835 | GO:(S)-scoulerine 9-O-methyltransferase activity | GO:0030777 |
| KEGG:R03840 | GO:ATP-dependent polydeoxyribonucleotide 5'-hydroxyl-kinase activity | GO:0046404 |
| KEGG:R03854 | GO:2,2-dialkylglycine decarboxylase (pyruvate) activity | GO:0047432 |
| KEGG:R03859 | GO:decylhomocitrate synthase activity | GO:0050458 |
| KEGG:R03860 | GO:geissoschizine dehydrogenase activity | GO:0047920 |
| KEGG:R03876 | GO:ubiquitin-protein transferase activity | GO:0004842 |
| KEGG:R03877 | GO:magnesium chelatase activity | GO:0016851 |
| KEGG:R03885 | GO:1-methyladenosine nucleosidase activity | GO:0047518 |
| KEGG:R03886 | GO:omega-hydroxydecanoate dehydrogenase activity | GO:0050153 |
| KEGG:R03887 | GO:2-aminomuconate deaminase activity | GO:0050540 |
| KEGG:R03894 | GO:branched-chain-2-oxoacid decarboxylase activity | GO:0047433 |
| KEGG:R03896 | GO:(R)-2-methylmalate dehydratase activity | GO:0047508 |
| KEGG:R03899 | GO:N1-acetylspermine:oxygen oxidoreductase (3-acetamidopropanal-forming) activity | GO:0052903 |
| KEGG:R03900 | GO:beta-nitroacrylate reductase activity | GO:0047703 |
| KEGG:R03906 | GO:glycyrrhizinate beta-glucuronidase activity | GO:0047967 |
| KEGG:R03908 | GO:2-methyleneglutarate mutase activity | GO:0047548 |
| KEGG:R03918 | GO:sinapyl alcohol dehydrogenase activity | GO:0052747 |
| KEGG:R03925 | GO:allantoin racemase activity | GO:0047653 |
| KEGG:R03930 | GO:3-methyleneoxindole reductase activity | GO:0047567 |
| KEGG:R03932 | GO:4-chlorobenzoate-CoA ligase activity | GO:0018861 |
| KEGG:R03934 | GO:6-hydroxymellein O-methyltransferase activity | GO:0030773 |
| KEGG:R03939 | GO:hypotaurocyamine kinase activity | GO:0047715 |
| KEGG:R03942 | GO:L-rhamnose 1-dehydrogenase activity | GO:0050034 |
| KEGG:R03944 | GO:alkylglycerone kinase activity | GO:0047650 |
| KEGG:R03945 | GO:perillyl-alcohol dehydrogenase activity | GO:0018457 |
| KEGG:R03947 | GO:precorrin-2 dehydrogenase activity | GO:0043115 |
| KEGG:R03948 | GO:precorrin-2 C20-methyltransferase activity | GO:0030788 |
| KEGG:R03952 | GO:thyroid-hormone transaminase activity | GO:0033852 |
| KEGG:R03963 | GO:2-alkyn-1-ol dehydrogenase activity | GO:0047535 |
| KEGG:R03964 | GO:2-hydroxybiphenyl 3-monooxygenase activity | GO:0047544 |
| KEGG:R03965 | GO:tartronate O-hydroxycinnamoyltransferase activity | GO:0047161 |
| KEGG:R03969 | GO:ADP-phosphoglycerate phosphatase activity | GO:0047630 |
| KEGG:R03994 | GO:piperidine N-piperoyltransferase activity | GO:0050199 |
| KEGG:R04002 | GO:ATP:2-methylpropanoate phosphotransferase activity | GO:0047758 |
| KEGG:R04003 | GO:S-carboxymethylcysteine synthase activity | GO:0050272 |
| KEGG:R04010 | GO:thiocyanate isomerase activity | GO:0050335 |
| KEGG:R04013 | GO:16-methoxy-2,3-dihydro-3-hydroxytabersonine N-methyltransferase activity | GO:0030768 |
| KEGG:R04020 | GO:indole-3-acetonitrile nitrile hydratase activity | GO:0080109 |
| KEGG:R04030 | GO:o-succinylbenzoate-CoA ligase activity | GO:0008756 |
| KEGG:R04034 | GO:phosphatidylserine 1-acylhydrolase activity | GO:0052739 |
| KEGG:R04035 | GO:phosphoribosyl-ATP diphosphatase activity | GO:0004636 |
| KEGG:R04037 | GO:phosphoribosyl-AMP cyclohydrolase activity | GO:0004635 |
| KEGG:R04038 | GO:AMP dimethylallyltransferase activity | GO:0009824 |
| KEGG:R04040 | GO:alpha-pinene-oxide decyclase activity | GO:0018851 |
| KEGG:R04043 | GO:acetylalkylglycerol acetylhydrolase activity | GO:0047378 |
| KEGG:R04050 | GO:3,4-dichloroaniline N-malonyltransferase activity | GO:0047163 |
| KEGG:R04053 | GO:phosphonopyruvate decarboxylase activity | GO:0033980 |
| KEGG:R04056 | GO:4-acetamidobutyryl-CoA deacetylase activity | GO:0047574 |
| KEGG:R04057 | GO:5-hydroxypentanoate CoA-transferase activity | GO:0047591 |
| KEGG:R04059 | GO:trans-acenaphthene-1,2-diol dehydrogenase activity | GO:0047062 |
| KEGG:R04060 | GO:anhydrotetracycline monooxygenase activity | GO:0047670 |
| KEGG:R04066 | GO:imidazoleacetate 4-monooxygenase activity | GO:0047717 |
| KEGG:R04068 | GO:imidazoleacetate-phosphoribosyldiphosphate ligase activity | GO:0047483 |
| KEGG:R04071 | GO:cytokinin 7-beta-glucosyltransferase activity | GO:0047807 |
| KEGG:R04077 | GO:tetrahydrocolumbamine 2-O-methyltransferase activity | GO:0030762 |
| KEGG:R04080 | GO:beta-cyclopiazonate dehydrogenase activity | GO:0050448 |
| KEGG:R04091 | GO:L-mimosine synthase activity | GO:0050461 |
| KEGG:R04093 | GO:indoleacetaldoxime dehydratase activity | GO:0047720 |
| KEGG:R04099 | GO:3-alpha-hydroxyglycyrrhetinate dehydrogenase activity | GO:0047032 |
| KEGG:R04105 | GO:(R)-3-hydroxyacid-ester dehydrogenase activity | GO:0047108 |
| KEGG:R04106 | GO:(S)-3-hydroxyacid-ester dehydrogenase activity | GO:0047109 |
| KEGG:R04109 | GO:glutamyl-tRNA reductase activity | GO:0008883 |
| KEGG:R04113 | GO:D-benzoylarginine-4-nitroanilide amidase activity | GO:0047415 |
| KEGG:R04119 | GO:phorbol-diester hydrolase activity | GO:0050181 |
| KEGG:R04126 | GO:alkylglycerol kinase activity | GO:0047649 |
| KEGG:R04130 | GO:2,6-dihydroxypyridine 3-monooxygenase activity | GO:0018663 |
| KEGG:R04135 | GO:hydroxyglutamate decarboxylase activity | GO:0047990 |
| KEGG:R04136 | GO:3-hydroxybenzyl-alcohol dehydrogenase activity | GO:0047048 |
| KEGG:R04138 | GO:methylcrotonoyl-CoA carboxylase activity | GO:0004485 |
| KEGG:R04139 | GO:3-alpha-hydroxycholanate dehydrogenase activity | GO:0047043 |
| KEGG:R04141 | GO:methylumbelliferyl-acetate deacetylase activity | GO:0047374 |
| KEGG:R04143 | GO:S-methyl-5-thioribose kinase activity | GO:0046522 |
| KEGG:R04144 | GO:phosphoribosylamine-glycine ligase activity | GO:0004637 |
| KEGG:R04147 | GO:isopenicillin-N epimerase activity | GO:0045439 |
| KEGG:R04148 | GO:nicotinate-nucleotide-dimethylbenzimidazole phosphoribosyltransferase activity | GO:0008939 |
| KEGG:R04150 | GO:1,4-dihydroxy-2-naphthoyl-CoA synthase activity | GO:0008935 |
| KEGG:R04151 | GO:cis-3,4-dihydrophenanthrene-3,4-diol dehydrogenase activity | GO:0018507 |
| KEGG:R04152 | GO:2-aminoethylphosphonate-pyruvate transaminase activity | GO:0047304 |
| KEGG:R04153 | GO:thetin-homocysteine S-methyltransferase activity | GO:0047149 |
| KEGG:R04154 | GO:tetrahydroxypteridine cycloisomerase activity | GO:0050329 |
| KEGG:R04158 | GO:phosphoglycerol geranylgeranyltransferase activity | GO:0047294 |
| KEGG:R04165 | GO:corticosteroid side-chain-isomerase activity | GO:0004110 |
| KEGG:R04167 | GO:2-carboxy-D-arabinitol-1-phosphatase activity | GO:0047538 |
| KEGG:R04169 | GO:isobutyraldoxime O-methyltransferase activity | GO:0030763 |
| KEGG:R04188 | GO:(S)-3-amino-2-methylpropionate transaminase activity | GO:0047298 |
| KEGG:R04189 | GO:N-succinylarginine dihydrolase activity | GO:0009015 |
| KEGG:R04202 | GO:bis(2-ethylhexyl)phthalate esterase activity | GO:0047709 |
| KEGG:R04205 | GO:13-hydroxylupinine O-tigloyltransferase activity | GO:0047203 |
| KEGG:R04208 | GO:phosphoribosylformylglycinamidine cyclo-ligase activity | GO:0004641 |
| KEGG:R04209 | GO:phosphoribosylaminoimidazole carboxylase activity | GO:0004638 |
| KEGG:R04210 | GO:4-phosphoerythronate dehydrogenase activity | GO:0033711 |
| KEGG:R04211 | GO:2,3-diaminopropionate N-oxalyltransferase activity | GO:0047189 |
| KEGG:R04213 | GO:tyrosine-ester sulfotransferase activity | GO:0017067 |
| KEGG:R04217 | GO:succinylornithine transaminase activity | GO:0043825 |
| KEGG:R04222 | GO:dTDP-dihydrostreptose-streptidine-6-phosphate dihydrostreptosyltransferase activity | GO:0047282 |
| KEGG:R04227 | GO:phosphatidylcholine-dolichol O-acyltransferase activity | GO:0047199 |
| KEGG:R04234 | GO:D-4-hydroxyphenylglycine transaminase activity | GO:0047320 |
| KEGG:R04235 | GO:5-methyldeoxycytidine-5'-phosphate kinase activity | GO:0047336 |
| KEGG:R04236 | GO:propanediol-phosphate dehydrogenase activity | GO:0050216 |
| KEGG:R04237 | GO:magnesium protoporphyrin IX methyltransferase activity | GO:0046406 |
| KEGG:R04256 | GO:leukotriene-E4 20-monooxygenase activity | GO:0050052 |
| KEGG:R04266 | GO:CDP-abequose epimerase activity | GO:0047732 |
| KEGG:R04277 | GO:2-pyrone-4,6-dicarboxylate lactonase activity | GO:0047554 |
| KEGG:R04279 | GO:4-carboxy-2-hydroxymuconate semialdehyde hemiacetal dehydrogenase activity | GO:0050606 |
| KEGG:R04286 | GO:6-pyruvoyltetrahydropterin synthase activity | GO:0003874 |
| KEGG:R04303 | GO:4-(dimethylamino)phenylazoxybenzene reductase activity | GO:0047136 |
| KEGG:R04307 | GO:4-hydroxycyclohexanecarboxylate dehydrogenase activity | GO:0047030 |
| KEGG:R04317 | GO:methylphosphothioglycerate phosphatase activity | GO:0047382 |
| KEGG:R04323 | GO:aminocarboxymuconate-semialdehyde decarboxylase activity | GO:0001760 |
| KEGG:R04327 | GO:juglone 3-monooxygenase activity | GO:0050012 |
| KEGG:R04333 | GO:indoleacetylglucose-inositol O-acyltransferase activity | GO:0047194 |
| KEGG:R04334 | GO:indolylacetyl-myo-inositol galactosyltransferase activity | GO:0047227 |
| KEGG:R04335 | GO:indolylacetylinositol arabinosyltransferase activity | GO:0050409 |
| KEGG:R04338 | GO:cysteine-conjugate transaminase activity | GO:0047802 |
| KEGG:R04344 | GO:5alpha-androstane-3beta,17beta-diol dehydrogenase activity | GO:0047024 |
| KEGG:R04347 | GO:tetrahydromethanopterin S-methyltransferase activity | GO:0030269 |
| KEGG:R04349 | GO:bromoxynil nitrilase activity | GO:0018761 |
| KEGG:R04350 | GO:mimosinase activity | GO:0050101 |
| KEGG:R04357 | GO:aerobactin synthase activity | GO:0050565 |
| KEGG:R04359 | GO:sarsapogenin 3-beta-glucosyltransferase activity | GO:0047249 |
| KEGG:R04364 | GO:tetrahydrodipicolinate N-acetyltransferase activity | GO:0047200 |
| KEGG:R04365 | GO:2,3,4,5-tetrahydropyridine-2,6-dicarboxylate N-succinyltransferase activity | GO:0008666 |
| KEGG:R04366 | GO:linalool 8-monooxygenase activity | GO:0050056 |
| KEGG:R04367 | GO:3-ketovalidoxylamine C-N-lyase activity | GO:0047566 |
| KEGG:R04370 | GO:benzyl-2-methyl-hydroxybutyrate dehydrogenase activity | GO:0047027 |
| KEGG:R04371 | GO:homoaconitate hydratase activity | GO:0004409 |
| KEGG:R04374 | GO:trans-L-3-hydroxyproline dehydratase activity | GO:0050346 |
| KEGG:R04383 | GO:4-deoxy-L-threo-5-hexosulose-uronate ketol-isomerase activity | GO:0008697 |
| KEGG:R04387 | GO:diethyl 2-methyl-3-oxosuccinate reductase activity | GO:0047031 |
| KEGG:R04388 | GO:opheline kinase activity | GO:0050154 |
| KEGG:R04392 | GO:steroid 9-alpha-monooxygenase activity | GO:0050292 |
| KEGG:R04397 | GO:2-(hydroxymethyl)-3-(acetamidomethylene)succinate hydrolase activity | GO:0047414 |
| KEGG:R04398 | GO:8-hydroxyquercitin 8-O-methyltransferase activity | GO:0030761 |
| KEGG:R04400 | GO:(S)-canadine synthase activity | GO:0047056 |
| KEGG:R04404 | GO:phosphatidylinositol-4,5-bisphosphate 5-phosphatase activity | GO:0004439 |
| KEGG:R04405 | GO:5-methyltetrahydropteroyltriglutamate-homocysteine S-methyltransferase activity | GO:0003871 |
| KEGG:R04412 | GO:vomifoliol 4'-dehydrogenase activity | GO:0050396 |
| KEGG:R04420 | GO:S-methyl-5-thioribose-1-phosphate isomerase activity | GO:0046523 |
| KEGG:R04421 | GO:N-benzoyl-4-hydroxyanthranilate 4-O-methyltransferase activity | GO:0030771 |
| KEGG:R04424 | GO:2-methylcitrate dehydratase activity | GO:0047547 |
| KEGG:R04425 | GO:2-methylisocitrate dehydratase activity | GO:0047456 |
| KEGG:R04428 | GO:crotonoyl-[acyl-carrier-protein] hydratase activity | GO:0047450 |
| KEGG:R04431 | GO:lactosylceramide beta-1,3-galactosyltransferase activity | GO:0047240 |
| KEGG:R04436 | GO:dimethylhistidine N-methyltransferase activity | GO:0030745 |
| KEGG:R04438 | GO:dTDP-4-amino-4,6-dideoxygalactose transaminase activity | GO:0019180 |
| KEGG:R04448 | GO:hydroxyethylthiazole kinase activity | GO:0004417 |
| KEGG:R04449 | GO:[citrate (pro-3S)-lyase] ligase activity | GO:0008771 |
| KEGG:R04451 | GO:steroid N-acetylglucosaminyltransferase activity | GO:0047261 |
| KEGG:R04455 | GO:N5,N10-methenyltetrahydromethanopterin hydrogenase activity | GO:0047068 |
| KEGG:R04456 | GO:methylenetetrahydromethanopterin dehydrogenase activity | GO:0030268 |
| KEGG:R04462 | GO:3-hydroxypalmitoyl-[acyl-carrier-protein] dehydratase activity | GO:0004317 |
| KEGG:R04463 | GO:phosphoribosylformylglycinamidine synthase activity | GO:0004642 |
| KEGG:R04464 | GO:coenzyme F420-dependent N5,N10-methenyltetrahydromethanopterin reductase activity | GO:0018537 |
| KEGG:R04469 | GO:digalactosyldiacylglycerol synthase activity | GO:0046481 |
| KEGG:R04475 | GO:succinyldiaminopimelate transaminase activity | GO:0009016 |
| KEGG:R04476 | GO:UDP-N-acetylgalactosamine-4-sulfate sulfotransferase activity | GO:0047368 |
| KEGG:R04478 | GO:4-oxalmesaconate hydratase activity | GO:0047584 |
| KEGG:R04490 | GO:acetoxybutynylbithiophene deacetylase activity | GO:0047373 |
| KEGG:R04496 | GO:protein C-terminal S-isoprenylcysteine carboxyl O-methyltransferase activity | GO:0004671 |
| KEGG:R04498 | GO:beta-glucogallin-tetrakisgalloylglucose O-galloyltransferase activity | GO:0047176 |
| KEGG:R04505 | GO:methylquercetagetin 6-O-methyltransferase activity | GO:0030759 |
| KEGG:R04510 | GO:4-carboxymethyl-4-methylbutenolide mutase activity | GO:0047469 |
| KEGG:R04520 | GO:geranylgeranylglycerol-phosphate geranylgeranyltransferase activity | GO:0047295 |
| KEGG:R04522 | GO:DDT-dehydrochlorinase activity | GO:0018833 |
| KEGG:R04525 | GO:5-(3,4-diacetoxybut-1-ynyl)-2,2'-bithiophene deacetylase activity | GO:0047377 |
| KEGG:R04527 | GO:mucinaminylserine mucinaminidase activity | GO:0050110 |
| KEGG:R04529 | GO:UDP-2-acetamido-4-amino-2,4,6-trideoxyglucose transaminase activity | GO:0047302 |
| KEGG:R04537 | GO:3-hydroxyoctanoyl-[acyl-carrier-protein] dehydratase activity | GO:0047451 |
| KEGG:R04538 | GO:furylfuramide isomerase activity | GO:0047907 |
| KEGG:R04541 | GO:coenzyme-B sulfoethylthiotransferase activity | GO:0050524 |
| KEGG:R04545 | GO:phosphatidylinositol-4,5-bisphosphate 3-kinase activity | GO:0046934 |
| KEGG:R04552 | GO:15-hydroxyprostaglandin dehydrogenase (NADP+) activity | GO:0047021 |
| KEGG:R04556 | GO:13-prostaglandin reductase activity | GO:0036132 |
| KEGG:R04556 | GO:15-oxoprostaglandin 13-oxidase activity | GO:0047522 |
| KEGG:R04557 | GO:13-prostaglandin reductase activity | GO:0036132 |
| KEGG:R04557 | GO:15-oxoprostaglandin 13-oxidase activity | GO:0047522 |
| KEGG:R04559 | GO:(S)-2-(5-amino-1-(5-phospho-D-ribosyl)imidazole-4-carboxamido)succinate AMP-lyase (fumarate-forming) activity | GO:0070626 |
| KEGG:R04565 | GO:prostaglandin-A1 delta-isomerase activity | GO:0050219 |
| KEGG:R04570 | GO:5-pyridoxate dioxygenase activity | GO:0047592 |
| KEGG:R04576 | GO:2-chloro-4-carboxymethylenebut-2-en-1,4-olide isomerase activity | GO:0047466 |
| KEGG:R04577 | GO:nuatigenin 3-beta-glucosyltransferase activity | GO:0047248 |
| KEGG:R04591 | GO:phosphoribosylaminoimidazolesuccinocarboxamide synthase activity | GO:0004639 |
| KEGG:R04594 | GO:alpha-ribazole phosphatase activity | GO:0043755 |
| KEGG:R04597 | GO:3,4-dihydroxy-9,10-secoandrosta-1,3,5(10)-triene-9,17-dione 4,5-dioxygenase activity | GO:0047071 |
| KEGG:R04602 | GO:chloridazon-catechol dioxygenase activity | GO:0047744 |
| KEGG:R04606 | GO:lipid-A-disaccharide synthase activity | GO:0008915 |
| KEGG:R04623 | GO:dihydrobunolol dehydrogenase activity | GO:0047855 |
| KEGG:R04635 | GO:(alpha-N-acetylneuraminyl-2,3-beta-galactosyl-1,3)-N-acetyl-galactosaminide 6-alpha-sialyltransferase activity | GO:0047290 |
| KEGG:R04640 | GO:1-(5-phosphoribosyl)-5-[(5-phosphoribosylamino)methylideneamino]imidazole-4-carboxamide isomerase activity | GO:0003949 |
| KEGG:R04657 | GO:tetraacyldisaccharide 4'-kinase activity | GO:0009029 |
| KEGG:R04690 | GO:(S)-stylopine synthase activity | GO:0047052 |
| KEGG:R04695 | GO:1,2-dehydroreticulinium reductase (NADPH) activity | GO:0047128 |
| KEGG:R04696 | GO:salutaridine synthase activity | GO:0047055 |
| KEGG:R04697 | GO:salutaridine reductase (NADPH) activity | GO:0047037 |
| KEGG:R04699 | GO:protopine 6-monooxygenase activity | GO:0047087 |
| KEGG:R04702 | GO:dihydrosanguinarine 10-monooxygenase activity | GO:0047088 |
| KEGG:R04705 | GO:12-hydroxydihydrochelirubine 12-O-methyltransferase activity | GO:0030780 |
| KEGG:R04707 | GO:10-hydroxydihydrosanguinarine 10-O-methyltransferase activity | GO:0030779 |
| KEGG:R04708 | GO:dihydrochelirubine 12-monooxygenase activity | GO:0047089 |
| KEGG:R04723 | GO:salutaridinol 7-O-acetyltransferase activity | GO:0047180 |
| KEGG:R04728 | GO:phaseollidin hydratase activity | GO:0047454 |
| KEGG:R04734 | GO:4-alpha-hydroxytetrahydrobiopterin dehydratase activity | GO:0008124 |
| KEGG:R04736 | GO:2-aminohexano-6-lactam racemase activity | GO:0047463 |
| KEGG:R04826 | GO:7alpha-hydroxycholest-4-en-3-one 12alpha-hydroxylase activity | GO:0033778 |
| KEGG:R04870 | GO:N-(5-amino-5-carboxypentanoyl)-L-cysteinyl-D-valine synthase activity | GO:0050564 |
| KEGG:R04872 | GO:isopenicillin-N synthase activity | GO:0016216 |
| KEGG:R04878 | GO:ergothioneine biosynthesis from N-alpha,N-alpha,N-alpha-trimethyl-L-histidine | GO:0052711 |
| KEGG:R04950 | GO:cysteine-S-conjugate N-acetyltransferase activity | GO:0047198 |
| KEGG:R05049 | GO:succinylglutamate-semialdehyde dehydrogenase activity | GO:0043824 |
| KEGG:R05060 | GO:11-hydroxythromboxane B2 dehydrogenase activity | GO:0036133 |
| KEGG:R05092 | GO:ent-kaurene synthase activity | GO:0009899 |
| KEGG:R05124 | GO:codeinone reductase (NADPH) activity | GO:0047036 |
| KEGG:R05137 | GO:2-hydroxychromene-2-carboxylate isomerase activity | GO:0018845 |
| KEGG:R05148 | GO:terephthalate 1,2-dioxygenase activity | GO:0018628 |
| KEGG:R05151 | GO:isoquinoline 1-oxidoreductase activity | GO:0047121 |
| KEGG:R05154 | GO:4-hydroxyquinoline 3-monooxygenase activity | GO:0047093 |
| KEGG:R05156 | GO:2-aminobenzenesulfonate 2,3-dioxygenase activity | GO:0018627 |
| KEGG:R05158 | GO:2-hydroxyquinoline 8-monooxygenase activity | GO:0033766 |
| KEGG:R05173 | GO:arylmalonate decarboxylase activity | GO:0047436 |
| KEGG:R05176 | GO:ADP-glyceromanno-heptose 6-epimerase activity | GO:0008712 |
| KEGG:R05183 | GO:quinoline-4-carboxylate 2-oxidoreductase activity | GO:0047123 |
| KEGG:R05189 | GO:mycocerosate synthase activity | GO:0050111 |
| KEGG:R05198 | GO:ethanol:cytochrome c oxidoreductase activity | GO:0052935 |
| KEGG:R05202 | GO:inositol pentakisphosphate 2-kinase activity | GO:0035299 |
| KEGG:R05217 | GO:precorrin-3B synthase activity | GO:0043818 |
| KEGG:R05218 | GO:cob(II)yrinic acid a,c-diamide reductase activity | GO:0043784 |
| KEGG:R05219 | GO:precorrin-6A synthase (deacetylating) activity | GO:0043819 |
| KEGG:R05221 | GO:adenosylcobinamide kinase (ATP-specific) activity | GO:0036429 |
| KEGG:R05222 | GO:cobinamide phosphate guanylyltransferase activity | GO:0008820 |
| KEGG:R05224 | GO:hydrogenobyrinic acid a,c-diamide synthase (glutamine-hydrolysing) activity | GO:0043802 |
| KEGG:R05225 | GO:adenosylcobyric acid synthase (glutamine-hydrolyzing) activity | GO:0051921 |
| KEGG:R05226 | GO:adenosylcobinamide hydrolase activity | GO:0043756 |
| KEGG:R05227 | GO:cobaltochelatase activity | GO:0051116 |
| KEGG:R05229 | GO:deacetoxycephalosporin-C hydroxylase activity | GO:0045442 |
| KEGG:R05236 | GO:4-hydroxymuconic-semialdehyde dehydrogenase activity | GO:0018481 |
| KEGG:R05240 | GO:2,3-dihydroxy-2,3-dihydro-p-cumate dehydrogenase activity | GO:0018511 |
| KEGG:R05244 | GO:p-benzoquinone reductase (NADPH) activity | GO:0018541 |
| KEGG:R05271 | GO:4-(hydroxymethyl)benzenesulfonate dehydrogenase activity | GO:0018462 |
| KEGG:R05272 | GO:4-formylbenzenesulfonate dehydrogenase activity | GO:0018482 |
| KEGG:R05274 | GO:vanillate monooxygenase activity | GO:0018489 |
| KEGG:R05275 | GO:phthalate 4,5-cis-dihydrodiol dehydrogenase activity | GO:0018517 |
| KEGG:R05276 | GO:2,4-dichlorobenzoyl-CoA reductase activity | GO:0018516 |
| KEGG:R05283 | GO:6-hydroxyhexanoate dehydrogenase activity | GO:0018463 |
| KEGG:R05285 | GO:2-chloroethanol:cytochrome c oxidoreductase activity | GO:0052936 |
| KEGG:R05301 | GO:deacetoxycephalosporin-C synthase activity | GO:0050599 |
| KEGG:R05305 | GO:3-hydroxypimeloyl-CoA dehydrogenase activity | GO:0018464 |
| KEGG:R05308 | GO:phloroglucinol reductase activity | GO:0018510 |
| KEGG:R05310 | GO:dibenzothiophene dihydrodiol dehydrogenase activity | GO:0018513 |
| KEGG:R05311 | GO:pimeloyl-CoA dehydrogenase activity | GO:0018515 |
| KEGG:R05312 | GO:5,6-dihydroxy-3-methyl-2-oxo-1,2,5,6-tetrahydroquinoline dehydrogenase activity | GO:0018518 |
| KEGG:R05313 | GO:cis-dihydroethylcatechol dehydrogenase activity | GO:0018519 |
| KEGG:R05314 | GO:1,2-dihydroxy-6-methylcyclohexa-3,5-dienecarboxylate dehydrogenase activity | GO:0018521 |
| KEGG:R05315 | GO:hydroxycyclohexanecarboxylate dehydrogenase activity | GO:0047010 |
| KEGG:R05316 | GO:4-hydroxybenzoyl-CoA reductase activity | GO:0018525 |
| KEGG:R05317 | GO:tryptophan alpha,beta-oxidase activity | GO:0050621 |
| KEGG:R05320 | GO:taurine dioxygenase activity | GO:0000908 |
| KEGG:R05323 | GO:3-methylquercitin 7-O-methyltransferase activity | GO:0030757 |
| KEGG:R05324 | GO:cis-p-coumarate glucosyltransferase activity | GO:0050644 |
| KEGG:R05328 | GO:glucosylglycerol-phosphate synthase activity | GO:0033828 |
| KEGG:R05330 | GO:propionyl-CoA C2-trimethyltridecanoyltransferase activity | GO:0050632 |
| KEGG:R05332 | GO:glucosamine-1-phosphate N-acetyltransferase activity | GO:0019134 |
| KEGG:R05334 | GO:methylputrescine oxidase activity | GO:0052599 |
| KEGG:R05337 | GO:4-hydroxybutanoyl-CoA dehydratase activity | GO:0043721 |
| KEGG:R05357 | GO:proclavaminate amidinohydrolase activity | GO:0033972 |
| KEGG:R05378 | GO:5-dehydro-2-deoxyphosphogluconate aldolase activity | GO:0047441 |
| KEGG:R05380 | GO:acetylene hydratase activity | GO:0018818 |
| KEGG:R05399 | GO:2-hydroxy-1,4-benzoquinone reductase activity | GO:0050625 |
| KEGG:R05452 | GO:benzoyl acetate-CoA ligase activity | GO:0018856 |
| KEGG:R05465 | GO:N2-(2-carboxyethyl)arginine synthase activity | GO:0033848 |
| KEGG:R05467 | GO:(carboxyethyl)arginine beta-lactam-synthase activity | GO:0034027 |
| KEGG:R05553 | GO:4-amino-4-deoxychorismate lyase activity | GO:0008696 |
| KEGG:R05558 | GO:atrazine chlorohydrolase activity | GO:0018788 |
| KEGG:R05567 | GO:deisopropylatrazine monooxygenase activity | GO:0018681 |
| KEGG:R05588 | GO:succinyl-CoA:(R)-benzylsuccinate CoA-transferase activity | GO:0033877 |
| KEGG:R05597 | GO:cyclohexa-1,5-dienecarbonyl-CoA hydratase activity | GO:0018823 |
| KEGG:R05598 | GO:benzylsuccinate synthase activity | GO:0018805 |
| KEGG:R05606 | GO:mannonate dehydratase activity | GO:0008927 |
| KEGG:R05608 | GO:galactarate dehydratase activity | GO:0008867 |
| KEGG:R05612 | GO:trans-hexaprenyltranstransferase activity | GO:0000010 |
| KEGG:R05613 | GO:trans-pentaprenyltranstransferase activity | GO:0048045 |
| KEGG:R05623 | GO:trimethylamine monooxygenase activity | GO:0034899 |
| KEGG:R05626 | GO:di-trans, poly-cis-undecaprenol kinase activity | GO:0036433 |
| KEGG:R05627 | GO:undecaprenyl-diphosphatase activity | GO:0050380 |
| KEGG:R05633 | GO:2-C-methyl-D-erythritol 4-phosphate cytidylyltransferase activity | GO:0050518 |
| KEGG:R05634 | GO:4-(cytidine 5'-diphospho)-2-C-methyl-D-erythritol kinase activity | GO:0050515 |
| KEGG:R05636 | GO:1-deoxy-D-xylulose-5-phosphate synthase activity | GO:0008661 |
| KEGG:R05637 | GO:2-C-methyl-D-erythritol 2,4-cyclodiphosphate synthase activity | GO:0008685 |
| KEGG:R05641 | GO:delta24(24-1) sterol reductase activity | GO:0000246 |
| KEGG:R05651 | GO:sulfoacetaldehyde acetyltransferase activity | GO:0050487 |
| KEGG:R05652 | GO:taurine-pyruvate aminotransferase activity | GO:0031299 |
| KEGG:R05678 | GO:sulcatone reductase activity | GO:0050491 |
| KEGG:R05682 | GO:1,5-anhydro-D-fructose reductase activity | GO:0050571 |
| KEGG:R05685 | GO:3-methylbutanol:NAD oxidoreductase activity | GO:0052676 |
| KEGG:R05686 | GO:3-methylbutanol:NADP oxidoreductase activity | GO:0052675 |
| KEGG:R05687 | GO:dTDP-4-dehydro-6-deoxyglucose reductase activity | GO:0050573 |
| KEGG:R05688 | GO:1-deoxy-D-xylulose-5-phosphate reductoisomerase activity | GO:0030604 |
| KEGG:R05689 | GO:2-(R)-hydroxypropyl-CoM dehydrogenase activity | GO:0050574 |
| KEGG:R05690 | GO:2-(S)-hydroxypropyl-CoM dehydrogenase activity | GO:0050575 |
| KEGG:R05699 | GO:vanillin dehydrogenase activity | GO:0050608 |
| KEGG:R05702 | GO:zeatin reductase activity | GO:0050472 |
| KEGG:R05705 | GO:FMN reductase (NADH) activity | GO:0052874 |
| KEGG:R05706 | GO:FMN reductase (NADPH) activity | GO:0052873 |
| KEGG:R05707 | GO:riboflavin reductase (NADPH) activity | GO:0042602 |
| KEGG:R05713 | GO:2-oxopropyl-CoM reductase (carboxylating) activity | GO:0050628 |
| KEGG:R05717 | GO:adenylyl-sulfate reductase (glutathione) activity | GO:0033741 |
| KEGG:R05718 | GO:linoleate 11-lipoxygenase activity | GO:0050584 |
| KEGG:R05720 | GO:3-hydroxy-2-methylquinolin-4-one 2,4-dioxygenase activity | GO:0050586 |
| KEGG:R05721 | GO:chlorite O2-lyase activity | GO:0050587 |
| KEGG:R05722 | GO:phytanoyl-CoA dioxygenase activity | GO:0048244 |
| KEGG:R05727 | GO:quinine 3-monooxygenase activity | GO:0050591 |
| KEGG:R05728 | GO:4-hydroxyphenylacetaldehyde oxime monooxygenase activity | GO:0050592 |
| KEGG:R05732 | GO:N-methylcoclaurine 3'-monooxygenase activity | GO:0050593 |
| KEGG:R05735 | GO:acetone carboxylase activity | GO:0018710 |
| KEGG:R05740 | GO:delta12-fatty acid dehydrogenase activity | GO:0016720 |
| KEGG:R05742 | GO:thiophene-2-carbonyl-CoA monooxygenase activity | GO:0050603 |
| KEGG:R05745 | GO:ethylbenzene hydroxylase activity | GO:0018693 |
| KEGG:R05746 | GO:phosphonate dehydrogenase activity | GO:0050609 |
| KEGG:R05748 | GO:methylarsonate reductase activity | GO:0050610 |
| KEGG:R05749 | GO:deacetylipecoside synthase activity | GO:0050557 |
| KEGG:R05750 | GO:deacetylisoipecoside synthase activity | GO:0050556 |
| KEGG:R05752 | GO:arsenate reductase (donor) activity | GO:0050612 |
| KEGG:R05757 | GO:3'-demethylstaurosporine O-methyltransferase activity | GO:0030793 |
| KEGG:R05758 | GO:threo-3-hydroxyaspartate ammonia-lyase activity | GO:0030848 |
| KEGG:R05760 | GO:cycloartenol 24-C-methyltransferase activity | GO:0030796 |
| KEGG:R05763 | GO:trans-aconitate 2-methyltransferase activity | GO:0030798 |
| KEGG:R05764 | GO:trans-aconitate 3-methyltransferase activity | GO:0046547 |
| KEGG:R05768 | GO:mannosyl-3-phosphoglycerate synthase activity | GO:0050504 |
| KEGG:R05769 | GO:hydroquinone glucosyltransferase activity | GO:0050505 |
| KEGG:R05771 | GO:cyclohexyl-isocyanide hydratase activity | GO:0050549 |
| KEGG:R05773 | GO:vanillin synthase activity | GO:0050547 |
| KEGG:R05774 | GO:sulfopyruvate decarboxylase activity | GO:0050545 |
| KEGG:R05775 | GO:UDPsulfoquinovose synthase activity | GO:0046507 |
| KEGG:R05776 | GO:24-methylenesterol C-methyltransferase activity | GO:0030797 |
| KEGG:R05777 | GO:diphosphoinositol-polyphosphate diphosphatase activity | GO:0008486 |
| KEGG:R05779 | GO:inositol diphosphate pentakisphosphate diphosphatase activity | GO:0052842 |
| KEGG:R05780 | GO:thiocyanate hydrolase activity | GO:0018760 |
| KEGG:R05781 | GO:maleimide hydrolase activity | GO:0050539 |
| KEGG:R05783 | GO:mandelamide amidase activity | GO:0050537 |
| KEGG:R05789 | GO:2-phosphosulfolactate phosphatase activity | GO:0050532 |
| KEGG:R05790 | GO:mannosyl-3-phosphoglycerate phosphatase activity | GO:0050531 |
| KEGG:R05791 | GO:glucosylglycerol 3-phosphatase activity | GO:0050530 |
| KEGG:R05794 | GO:phosphatidylcholine synthase activity | GO:0050520 |
| KEGG:R05795 | GO:1-phosphatidylinositol-4-phosphate 3-kinase activity | GO:0035005 |
| KEGG:R05799 | GO:inositol hexakisphosphate 1-kinase activity | GO:0052723 |
| KEGG:R05800 | GO:inositol-1,4,5-trisphosphate 6-kinase activity | GO:0000823 |
| KEGG:R05802 | GO:1-phosphatidylinositol-3-phosphate 5-kinase activity | GO:0000285 |
| KEGG:R05825 | GO:polyneuridine-aldehyde esterase activity | GO:0050529 |
| KEGG:R05827 | GO:vellosimine dehydrogenase activity | GO:0050579 |
| KEGG:R05828 | GO:deoxysarpagine hydroxylase activity | GO:0033775 |
| KEGG:R05838 | GO:pyridoxine 5'-phosphate synthase activity | GO:0033856 |
| KEGG:R05850 | GO:L-ribulose-phosphate 4-epimerase activity | GO:0008742 |
| KEGG:R05855 | GO:tabersonine 16-hydroxylase activity | GO:0050594 |
| KEGG:R05857 | GO:desacetoxyvindoline 4-hydroxylase activity | GO:0050590 |
| KEGG:R05876 | GO:vinorine synthase activity | GO:0050636 |
| KEGG:R05877 | GO:vinorine hydroxylase activity | GO:0050596 |
| KEGG:R05878 | GO:vomilenine reductase activity | GO:0050624 |
| KEGG:R05879 | GO:1,2-dihydrovomilenine reductase activity | GO:0050615 |
| KEGG:R05882 | GO:vomilenine glucosyltransferase activity | GO:0050506 |
| KEGG:R05982 | GO:mannosyl-oligosaccharide 1,2-alpha-mannosidase activity | GO:0004571 |
| KEGG:R05983 | GO:alpha-1,3-mannosylglycoprotein 2-beta-N-acetylglucosaminyltransferase activity | GO:0003827 |
| KEGG:R05988 | GO:glycoprotein 6-alpha-L-fucosyltransferase activity | GO:0008424 |
| KEGG:R06015 | GO:glycoprotein 3-alpha-L-fucosyltransferase activity | GO:0018392 |
| KEGG:R06016 | GO:glycoprotein 2-beta-D-xylosyltransferase activity | GO:0050513 |
| KEGG:R06048 | GO:indoxyl-UDPG glucosyltransferase activity | GO:0050507 |
| KEGG:R06117 | GO:(+)-trans-carveol dehydrogenase activity | GO:0033702 |
| KEGG:R06119 | GO:(R)-limonene 6-monooxygenase activity | GO:0052741 |
| KEGG:R06120 | GO:(R)-limonene synthase activity | GO:0034002 |
| KEGG:R06126 | GO:serine 3-dehydrogenase activity | GO:0031132 |
| KEGG:R06166 | GO:3beta-hydroxy-5beta-steroid dehydrogenase activity | GO:0033703 |
| KEGG:R06211 | GO:sucrose-phosphate phosphatase activity | GO:0050307 |
| KEGG:R06258 | GO:dol-P-Man:Man(5)GlcNAc(2)-PP-Dol alpha-1,3-mannosyltransferase activity | GO:0052925 |
| KEGG:R06259 | GO:dol-P-Man:Man(6)GlcNAc(2)-PP-Dol alpha-1,2-mannosyltransferase activity | GO:0052926 |
| KEGG:R06260 | GO:dol-P-Man:Man(7)GlcNAc(2)-PP-Dol alpha-1,6-mannosyltransferase activity | GO:0052917 |
| KEGG:R06261 | GO:dol-P-Man:Man(8)GlcNAc(2)-PP-Dol alpha-1,2-mannosyltransferase activity | GO:0052918 |
| KEGG:R06284 | GO:chlorophyll synthetase activity | GO:0046408 |
| KEGG:R06298 | GO:copalyl diphosphate synthase activity | GO:0050559 |
| KEGG:R06301 | GO:abietadiene synthase activity | GO:0050554 |
| KEGG:R06302 | GO:levopimaradiene synthase activity | GO:0052678 |
| KEGG:R06305 | GO:taxadiene synthase activity | GO:0050553 |
| KEGG:R06306 | GO:taxadiene 5-alpha-hydroxylase activity | GO:0050604 |
| KEGG:R06307 | GO:taxadien-5-alpha-ol O-acetyltransferase activity | GO:0050638 |
| KEGG:R06308 | GO:taxane 13-alpha-hydroxylase activity | GO:0050598 |
| KEGG:R06309 | GO:taxane 10-beta-hydroxylase activity | GO:0050597 |
| KEGG:R06310 | GO:2-alpha-hydroxytaxane 2-O-benzoyltransferase activity | GO:0050642 |
| KEGG:R06311 | GO:10-deacetylbaccatin III 10-O-acetyltransferase activity | GO:0050643 |
| KEGG:R06313 | GO:aphidicolan-16 beta-ol synthase activity | GO:0046567 |
| KEGG:R06351 | GO:abieta-7,13-diene hydroxylase activity | GO:0036189 |
| KEGG:R06354 | GO:abieta-7,13-dien-18-ol hydroxylase activity | GO:0036204 |
| KEGG:R06357 | GO:abieta-7,13-dien-18-al dehydrogenase activity | GO:0036188 |
| KEGG:R06367 | GO:perillic acid:CoA ligase (ADP-forming) activity | GO:0052685 |
| KEGG:R06368 | GO:perillic acid:CoA ligase (AMP-forming) activity | GO:0052686 |
| KEGG:R06396 | GO:(3R)-3-isopropenyl-6-oxoheptanoate:CoA ligase (ADP-forming) activity | GO:0052687 |
| KEGG:R06398 | GO:(R)-limonene 1,2-monooxygenase activity | GO:0018635 |
| KEGG:R06417 | GO:(-)-isopiperitenone reductase activity | GO:0052581 |
| KEGG:R06502 | GO:polyenoic fatty acid isomerase activity | GO:0034016 |
| KEGG:R06513 | GO:dTDP-glucose 4,6-dehydratase activity | GO:0008460 |
| KEGG:R06514 | GO:dTDP-4-dehydrorhamnose 3,5-epimerase activity | GO:0008830 |
| KEGG:R06515 | GO:(3R)-3-isopropenyl-6-oxoheptanoate:CoA ligase (AMP-forming) activity | GO:0052688 |
| KEGG:R06523 | GO:vetispiradiene synthase activity | GO:0034003 |
| KEGG:R06528 | GO:phytoceramidase activity | GO:0070774 |
| KEGG:R06530 | GO:threonine-phosphate decarboxylase activity | GO:0048472 |
| KEGG:R06558 | GO:adenosylcobinamide kinase (GTP-specific) activity | GO:0036428 |
| KEGG:R06601 | GO:hydroxyisourate hydrolase activity | GO:0033971 |
| KEGG:R06604 | GO:2-oxo-4-hydroxy-4-carboxy-5-ureidoimidazoline decarboxylase activity | GO:0051997 |
| KEGG:R06613 | GO:thymidylate synthase (FAD) activity | GO:0050797 |
| KEGG:R06722 | GO:mannosyl-oligosaccharide 1,2-alpha-mannosidase activity | GO:0004571 |
| KEGG:R06722 | GO:trimming of terminal mannose on B branch | GO:0036509 |
| KEGG:R06734 | GO:tropinone reductase activity | GO:0050358 |
| KEGG:R06786 | GO:3-(3-hydroxyphenyl)propionate hydroxylase activity | GO:0008688 |
| KEGG:R06807 | GO:kaempferol 4'-O-methyltransferase activity | GO:0033803 |
| KEGG:R06815 | GO:myricetin 3'-O-methyltransferase activity | GO:0033799 |
| KEGG:R06816 | GO:laricitrin 5'-O-methyltransferase activity | GO:0070448 |
| KEGG:R06827 | GO:luteolin-7-O-glucuronide 7-O-glucuronosyltransferase activity | GO:0047246 |
| KEGG:R06828 | GO:luteolin-7-O-diglucuronide 4'-O-glucuronosyltransferase activity | GO:0047247 |
| KEGG:R06836 | GO:ribose 1,5-bisphosphate phosphokinase activity | GO:0033863 |
| KEGG:R06846 | GO:quinate 3-dehydrogenase (NADP+) activity | GO:0052733 |
| KEGG:R06847 | GO:shikimate 3-dehydrogenase (NADP+) activity | GO:0004764 |
| KEGG:R06847 | GO:shikimate 3-dehydrogenase (NAD+) activity | GO:0052734 |
| KEGG:R06859 | GO:2-phytyl-1,4-naphthoquinone methyltransferase activity | GO:0052624 |
| KEGG:R06866 | GO:3-hexaprenyl-4-hydroxy-5-methoxybenzoic acid decarboxylase activity | GO:0036163 |
| KEGG:R06868 | GO:phosphomethylethanolamine N-methyltransferase activity | GO:0052667 |
| KEGG:R06870 | GO:phosphoethanolamine phosphatase activity | GO:0052732 |
| KEGG:R06871 | GO:phosphocholine phosphatase activity | GO:0052731 |
| KEGG:R06892 | GO:4-hydroxyacetophenone monooxygenase activity | GO:0033767 |
| KEGG:R06945 | GO:cyclohexane monooxygenase activity | GO:0034795 |
| KEGG:R06946 | GO:zeaxanthin epoxidase activity | GO:0052662 |
| KEGG:R06947 | GO:antheraxanthin epoxidase activity | GO:0052663 |
| KEGG:R06948 | GO:neoxanthin synthase activity | GO:0034020 |
| KEGG:R06954 | GO:xanthoxin dehydrogenase activity | GO:0010301 |
| KEGG:R06957 | GO:abscisic aldehyde oxidase activity | GO:0010293 |
| KEGG:R06977 | GO:diaminobutyrate-2-oxoglutarate transaminase activity | GO:0045303 |
| KEGG:R06978 | GO:diaminobutyrate acetyltransferase activity | GO:0033816 |
| KEGG:R06979 | GO:ectoine synthase activity | GO:0033990 |
| KEGG:R06982 | GO:S-(hydroxymethyl)glutathione synthase activity | GO:0051907 |
| KEGG:R06983 | GO:S-(hydroxymethyl)glutathione dehydrogenase activity | GO:0051903 |
| KEGG:R06988 | GO:3-(hydroxyamino)phenol mutase activity | GO:0034022 |
| KEGG:R07041 | GO:arachidonic acid omega-hydroxylase activity | GO:0052869 |
| KEGG:R07053 | GO:arachidonate 8(S)-lipoxygenase activity | GO:0036403 |
| KEGG:R07061 | GO:linoleate 8R-lipoxygenase activity | GO:0052878 |
| KEGG:R07062 | GO:9,12-octadecadienoate 8-hydroperoxide 8S-isomerase activity | GO:0052879 |
| KEGG:R07125 | GO:3-dehydro-L-gulonate-6-phosphate decarboxylase activity | GO:0033982 |
| KEGG:R07134 | GO:D-arabinitol 2-dehydrogenase activity | GO:0047038 |
| KEGG:R07135 | GO:mannitol dehydrogenase activity | GO:0046029 |
| KEGG:R07138 | GO:3beta-hydroxy-5alpha-steroid dehydrogenase activity | GO:0033704 |
| KEGG:R07140 | GO:S-(hydroxymethyl)glutathione dehydrogenase activity | GO:0051903 |
| KEGG:R07143 | GO:D-arabinitol dehydrogenase, D-xylulose forming (NADP+) activity | GO:0052677 |
| KEGG:R07144 | GO:D-arabinitol dehydrogenase, D-ribulose forming (NADP+) activity | GO:0033709 |
| KEGG:R07151 | GO:hydroxyphytanate oxidase activity | GO:0047996 |
| KEGG:R07153 | GO:dehydrogluconate dehydrogenase activity | GO:0047843 |
| KEGG:R07163 | GO:all-trans-retinol 13,14-reductase activity | GO:0051786 |
| KEGG:R07164 | GO:aspartate dehydrogenase activity | GO:0033735 |
| KEGG:R07165 | GO:aspartate dehydrogenase activity | GO:0033735 |
| KEGG:R07167 | GO:taurine dehydrogenase activity | GO:0050323 |
| KEGG:R07168 | GO:methylenetetrahydrofolate reductase (NAD(P)H) activity | GO:0004489 |
| KEGG:R07169 | GO:berberine reductase activity | GO:0050623 |
| KEGG:R07170 | GO:(R)-6-hydroxynicotine oxidase activity | GO:0018530 |
| KEGG:R07177 | GO:thiosulfate dehydrogenase (quinone) activity | GO:0043831 |
| KEGG:R07185 | GO:2'-deoxymugineic-acid 2'-dioxygenase activity | GO:0033760 |
| KEGG:R07186 | GO:mugineic-acid 3-dioxygenase activity | GO:0033761 |
| KEGG:R07187 | GO:2'-deoxymugineic-acid 3-dioxygenase activity | GO:0052860 |
| KEGG:R07198 | GO:licodione synthase activity | GO:0033771 |
| KEGG:R07199 | GO:zeaxanthin epoxidase activity | GO:0052662 |
| KEGG:R07200 | GO:antheraxanthin epoxidase activity | GO:0052663 |
| KEGG:R07201 | GO:phenylacetone monooxygenase activity | GO:0033776 |
| KEGG:R07202 | GO:(+)-abscisic acid 8'-hydroxylase activity | GO:0010295 |
| KEGG:R07203 | GO:lithocholate 6beta-hydroxylase activity | GO:0033777 |
| KEGG:R07204 | GO:5beta-cholestane-3alpha,7alpha-diol 12alpha-hydroxylase activity | GO:0033779 |
| KEGG:R07207 | GO:cholesterol 24-hydroxylase activity | GO:0033781 |
| KEGG:R07208 | GO:24-hydroxycholesterol 7alpha-hydroxylase activity | GO:0033782 |
| KEGG:R07214 | GO:1-aminocyclopropane-1-carboxylate oxidase activity | GO:0009815 |
| KEGG:R07218 | GO:cholesterol 25-hydroxylase activity | GO:0001567 |
| KEGG:R07221 | GO:6-hydroxynicotinate dehydrogenase activity | GO:0043732 |
| KEGG:R07222 | GO:phenylacetyl-CoA dehydrogenase activity | GO:0033789 |
| KEGG:R07226 | GO:glycine reductase activity | GO:0030699 |
| KEGG:R07229 | GO:selenate reductase activity | GO:0033797 |
| KEGG:R07232 | GO:23S rRNA (adenine(1618)-N(6))-methyltransferase activity | GO:0052907 |
| KEGG:R07233 | GO:23S rRNA (guanine(745)-N(1))-methyltransferase activity | GO:0052911 |
| KEGG:R07233 | GO:23S rRNA (guanine(748)-N(1))-methyltransferase activity | GO:0052912 |
| KEGG:R07234 | GO:16S rRNA (guanine(966)-N(2))-methyltransferase activity | GO:0052913 |
| KEGG:R07234 | GO:16S rRNA (guanine(1207)-N(2))-methyltransferase activity | GO:0052914 |
| KEGG:R07234 | GO:23S rRNA (guanine(2445)-N(2))-methyltransferase activity | GO:0052915 |
| KEGG:R07234 | GO:23S rRNA (guanine(1835)-N(2))-methyltransferase activity | GO:0052916 |
| KEGG:R07241 | GO:corydaline synthase activity | GO:0050631 |
| KEGG:R07242 | GO:isoliquiritigenin 2'-O-methyltransferase activity | GO:0033802 |
| KEGG:R07243 | GO:sarcosine N-methyltransferase activity | GO:0052730 |
| KEGG:R07244 | GO:dimethylglycine N-methyltransferase activity | GO:0052729 |
| KEGG:R07245 | GO:N-acetylornithine carbamoyltransferase activity | GO:0043857 |
| KEGG:R07248 | GO:trehalose O-mycolyltransferase activity | GO:0050348 |
| KEGG:R07250 | GO:acridone synthase activity | GO:0050635 |
| KEGG:R07251 | GO:lovastatin nonaketide synthase activity | GO:0050637 |
| KEGG:R07252 | GO:10-hydroxytaxane O-acetyltransferase activity | GO:0050639 |
| KEGG:R07253 | GO:6-methylsalicylic acid synthase activity | GO:0050641 |
| KEGG:R07257 | GO:ditrans, polycis-undecaprenyl-phosphate mannosyltransferase activity | GO:0036426 |
| KEGG:R07260 | GO:cis-zeatin O-beta-D-glucosyltransferase activity | GO:0050502 |
| KEGG:R07264 | GO:kojibiose phosphorylase activity | GO:0033831 |
| KEGG:R07267 | GO:trans-octaprenyltranstransferase activity | GO:0050347 |
| KEGG:R07276 | GO:glutamate-prephenate aminotransferase activity | GO:0033854 |
| KEGG:R07277 | GO:nicotianamine aminotransferase activity | GO:0033855 |
| KEGG:R07281 | GO:3,4-dihydroxy-2-butanone-4-phosphate synthase activity | GO:0008686 |
| KEGG:R07288 | GO:dermatan 6-sulfotransferase activity | GO:0036443 |
| KEGG:R07288 | GO:N-acetylgalactosamine 4-sulfate 6-O-sulfotransferase activity | GO:0050659 |
| KEGG:R07289 | GO:glycochenodeoxycholate sulfotransferase activity | GO:0033876 |
| KEGG:R07290 | GO:formyl-CoA transferase activity | GO:0033608 |
| KEGG:R07294 | GO:phenylacetyl-CoA hydrolase activity | GO:0033880 |
| KEGG:R07295 | GO:bile-acid-CoA hydrolase activity | GO:0033881 |
| KEGG:R07300 | GO:acetylspermidine deacetylase activity | GO:0047611 |
| KEGG:R07301 | GO:(S)-N-acetyl-1-phenylethylamine hydrolase activity | GO:0050536 |
| KEGG:R07306 | GO:GTP cyclohydrolase IIa activity | GO:0043740 |
| KEGG:R07307 | GO:dCTP deaminase (dUMP-forming) activity | GO:0033973 |
| KEGG:R07311 | GO:2'-hydroxybiphenyl-2-sulfinate desulfinase activity | GO:0018740 |
| KEGG:R07312 | GO:4-hydroxyphenylacetate decarboxylase activity | GO:0043722 |
| KEGG:R07313 | GO:D-dopachrome decarboxylase activity | GO:0033981 |
| KEGG:R07318 | GO:bile-acid 7alpha-dehydratase activity | GO:0033988 |
| KEGG:R07319 | GO:maltose epimerase activity | GO:0050558 |
| KEGG:R07324 | GO:inositol-3-phosphate synthase activity | GO:0004512 |
| KEGG:R07325 | GO:long-chain fatty acid [acyl-carrier-protein] ligase activity | GO:0008922 |
| KEGG:R07345 | GO:(S)-usnate reductase activity | GO:0046998 |
| KEGG:R07346 | GO:sorbose reductase activity | GO:0032115 |
| KEGG:R07353 | GO:pyrroloquinoline-quinone synthase activity | GO:0033732 |
| KEGG:R07359 | GO:NADPH dehydrogenase (quinone) activity | GO:0008753 |
| KEGG:R07363 | GO:acireductone dioxygenase (Ni2+-requiring) activity | GO:0010308 |
| KEGG:R07364 | GO:acireductone dioxygenase [iron(II)-requiring] activity | GO:0010309 |
| KEGG:R07373 | GO:senecionine N-oxygenase activity | GO:0033784 |
| KEGG:R07392 | GO:methylthioribulose 1-phosphate dehydratase activity | GO:0046570 |
| KEGG:R07394 | GO:2-hydroxy-3-keto-5-methylthiopentenyl-1-phosphate phosphatase activity | GO:0043716 |
| KEGG:R07403 | GO:indole-2-monooxygenase activity | GO:0036190 |
| KEGG:R07404 | GO:5-(carboxyamino)imidazole ribonucleotide synthase activity | GO:0034028 |
| KEGG:R07414 | GO:glutamate-putrescine ligase activity | GO:0034024 |
| KEGG:R07419 | GO:gamma-glutamyl-gamma-aminobutyrate hydrolase activity | GO:0033969 |
| KEGG:R07421 | GO:indolin-2-one monooxygenase activity | GO:0036191 |
| KEGG:R07422 | GO:3-hydroxyindolin-2-one monooxygenase activity | GO:0036192 |
| KEGG:R07423 | GO:2-hydroxy-1,4-benzoxazin-3-one monooxygenase activity | GO:0036193 |
| KEGG:R07476 | GO:phosphosulfolactate synthase activity | GO:0043817 |
| KEGG:R07500 | GO:homogentisate phytyltransferase activity | GO:0010176 |
| KEGG:R07502 | GO:gamma-tocopherol cyclase activity | GO:0052605 |
| KEGG:R07503 | GO:delta-tocopherol cyclase activity | GO:0052604 |
| KEGG:R07511 | GO:9,9'-di-cis-zeta-carotene desaturation to 7,9,7',9'-tetra-cis-lycopene | GO:0052889 |
| KEGG:R07582 | GO:10-hydroxy-9-(phosphonooxy)octadecanoate phosphatase activity | GO:0033885 |
| KEGG:R07598 | GO:L-lysine 6-oxidase activity | GO:0033736 |
| KEGG:R07605 | GO:preQ1 synthase activity | GO:0033739 |
| KEGG:R07606 | GO:L-methionine:thioredoxin-disulfide S-oxidoreductase activity | GO:0033744 |
| KEGG:R07607 | GO:peptide-methionine (R)-S-oxide reductase activity | GO:0033743 |
| KEGG:R07612 | GO:reactive-black-5:hydrogen-peroxide oxidoreductase activity | GO:0052750 |
| KEGG:R07613 | GO:L,L-diaminopimelate aminotransferase activity | GO:0010285 |
| KEGG:R07629 | GO:N-malonylurea hydrolase activity | GO:0033970 |
| KEGG:R07630 | GO:amorpha-4,11-diene synthase activity | GO:0034006 |
| KEGG:R07631 | GO:S-linalool synthase activity | GO:0034007 |
| KEGG:R07632 | GO:R-linalool synthase activity | GO:0034008 |
| KEGG:R07633 | GO:sulfolactate sulfo-lyase activity | GO:0034010 |
| KEGG:R07634 | GO:L-cysteate sulfo-lyase activity | GO:0034011 |
| KEGG:R07638 | GO:phenylacetaldoxime dehydratase activity | GO:0018814 |
| KEGG:R07647 | GO:germacradienol synthase activity | GO:0034004 |
| KEGG:R07648 | GO:germacrene-D synthase activity | GO:0052577 |
| KEGG:R07649 | GO:germacrene-A synthase activity | GO:0034005 |
| KEGG:R07650 | GO:diaminobutyrate decarboxylase activity | GO:0033983 |
| KEGG:R07796 | GO:cinnamoyl-CoA:phenyllactate CoA-transferase activity | GO:0043785 |
| KEGG:R07797 | GO:petromyzonol sulfotransferase activity | GO:0033873 |
| KEGG:R07798 | GO:scymnol sulfotransferase activity | GO:0033874 |
| KEGG:R07830 | GO:epi-isozizaene synthase activity | GO:0052680 |
| KEGG:R07918 | GO:N-methyl nucleosidase activity | GO:0033960 |
| KEGG:R07984 | GO:enamidase activity | GO:0043792 |
| KEGG:R07985 | GO:2-hydroxymethylglutarate dehydrogenase activity | GO:0043718 |
| KEGG:R08051 | GO:ADP dimethylallyltransferase activity | GO:0052623 |
| KEGG:R08052 | GO:ATP dimethylallyltransferase activity | GO:0052622 |
| KEGG:R08057 | GO:diguanylate cyclase activity | GO:0052621 |
| KEGG:R08088 | GO:citronellyl-CoA ligase activity | GO:0034823 |
| KEGG:R08090 | GO:3-hydroxy-3-isohexenylglutaryl-CoA lyase activity | GO:0047445 |
| KEGG:R08159 | GO:myristoyl-[acyl-carrier-protein] hydrolase activity | GO:0016295 |
| KEGG:R08162 | GO:palmitoyl-[acyl-carrier-protein] hydrolase activity | GO:0016296 |
| KEGG:R08165 | GO:2-succinyl-5-enolpyruvyl-6-hydroxy-3-cyclohexene-1-carboxylic-acid synthase activity | GO:0070204 |
| KEGG:R08166 | GO:2-succinyl-6-hydroxy-2,4-cyclohexadiene-1-carboxylate synthase activity | GO:0070205 |
| KEGG:R08193 | GO:phosphoacetylglucosamine mutase activity | GO:0004610 |
| KEGG:R08194 | GO:1,5-anhydro-D-fructose reductase (1,5-anhydro-D-mannitol-forming) activity | GO:0033712 |
| KEGG:R08199 | GO:isoprene synthase activity | GO:0034009 |
| KEGG:R08200 | GO:phosphonopyruvate hydrolase activity | GO:0033978 |
| KEGG:R08201 | GO:2-oxoglutarate carboxylase activity | GO:0034029 |
| KEGG:R08203 | GO:chlorophyllide a oxygenase activity | GO:0052606 |
| KEGG:R08204 | GO:7-hydroxy-chlorophyllide a oxygenase activity | GO:0052607 |
| KEGG:R08369 | GO:cytokinin 9-beta-glucosyltransferase activity | GO:0080062 |
| KEGG:R08370 | GO:alpha-bisabolene synthase activity | GO:0052681 |
| KEGG:R08379 | GO:NADP-retinol dehydrogenase activity | GO:0052650 |
| KEGG:R08388 | GO:all-trans-retinyl-ester hydrolase, 11-cis retinol forming activity | GO:0052885 |
| KEGG:R08528 | GO:Z-farnesyl diphosphate synthase activity | GO:0033850 |
| KEGG:R08547 | GO:tryptophan 2-C-methyltransferase activity | GO:0030772 |
| KEGG:R08549 | GO:oxoglutarate dehydrogenase (NAD+) activity | GO:0034602 |
| KEGG:R08573 | GO:erythrulose reductase activity | GO:0047880 |
| KEGG:R08689 | GO:4-hydroxy-3-methylbut-2-en-1-yl diphosphate synthase activity | GO:0046429 |
| KEGG:R08715 | GO:exo-1,4-beta-D-glucosaminidase activity | GO:0052761 |
| KEGG:R08856 | GO:UDP-N-acetylglucosamine-undecaprenyl-phosphate N-acetylglucosaminephosphotransferase activity | GO:0036380 |
| KEGG:R08889 | GO:apo-beta-carotenoid-14',13'-dioxygenase activity | GO:0050588 |
| KEGG:R08923 | GO:(+)-menthofuran synthase activity | GO:0052582 |
| KEGG:R08948 | GO:chrysanthemyl diphosphate synthase activity | GO:0033849 |
| KEGG:R08950 | GO:lavandulyl diphosphate synthase activity | GO:0033851 |
| KEGG:R08969 | GO:sphingomyelin synthase activity | GO:0033188 |
| KEGG:R08991 | GO:cyclic-guanylate-specific phosphodiesterase activity | GO:0071111 |
| KEGG:R09074 | GO:N1-acetylspermidine:oxygen oxidoreductase (3-acetamidopropanal-forming) activity | GO:0052904 |
| KEGG:R09075 | GO:N8-acetylspermidine:oxygen oxidoreductase (propane-1,3-diamine-forming) activity | GO:0052897 |
| KEGG:R09076 | GO:spermine:oxygen oxidoreductase (spermidine-forming) activity | GO:0052901 |
| KEGG:R09077 | GO:spermidine:oxygen oxidoreductase (3-aminopropanal-forming) activity | GO:0052902 |
| KEGG:R09078 | GO:diacetyl reductase ((S)-acetoin forming) activity | GO:0052588 |
| KEGG:R09087 | GO:inositol hexakisphosphate 5-kinase activity | GO:0000832 |
| KEGG:R09115 | GO:stemar-13-ene synthase activity | GO:0034278 |
| KEGG:R09116 | GO:syn-stemod-13(17)-ene synthase activity | GO:0034283 |
| KEGG:R09117 | GO:syn-pimara-7,15-diene synthase activity | GO:0034279 |
| KEGG:R09119 | GO:ent-cassa-12,15-diene synthase activity | GO:0034277 |
| KEGG:R09120 | GO:ent-sandaracopimaradiene synthase activity | GO:0034280 |
| KEGG:R09121 | GO:ent-pimara-8(14),15-diene synthase activity | GO:0034282 |
| KEGG:R09127 | GO:ethanol cytochrome-c oxidoreductase activity | GO:0052931 |
| KEGG:R09128 | GO:2-chloroethanol cytochrome-c oxidoreductase activity | GO:0052932 |
| KEGG:R09140 | GO:epi-cedrol synthase activity | GO:0052682 |
| KEGG:R09245 | GO:hexaprenyl-diphosphate synthase ((2E,6E)-farnesyl-diphosphate specific) activity | GO:0036423 |
| KEGG:R09246 | GO:hexaprenyl diphosphate synthase (geranylgeranyl-diphosphate specific) activity | GO:0052922 |
| KEGG:R09250 | GO:all-trans-nonaprenyl-diphosphate synthase (geranyl-diphosphate specific) activity | GO:0052923 |
| KEGG:R09251 | GO:all-trans-nonaprenyl-diphosphate synthase (geranylgeranyl-diphosphate specific) activity | GO:0052924 |
| KEGG:R09289 | GO:3-hydroxypropionate dehydrogenase (NADP+) activity | GO:0035527 |
| KEGG:R09358 | GO:(2R)-2-hydroxy-2-methylbutanenitrile lyase activity | GO:0052920 |
| KEGG:R09359 | GO:aromatic (S)-hydroxynitrile lyase activity | GO:0052892 |
| KEGG:R09382 | GO:tRNA 3'-terminal CCA addition | GO:0001680 |
| KEGG:R09383 | GO:CTP:tRNA cytidylyltransferase activity | GO:0052927 |
| KEGG:R09384 | GO:CTP:3'-cytidine-tRNA cytidylyltransferase activity | GO:0052928 |
| KEGG:R09385 | GO:(S)-limonene 1,2-monooxygenase activity | GO:0052601 |
| KEGG:R09386 | GO:ATP:3'-cytidine-cytidine-tRNA adenylyltransferase activity | GO:0052929 |
| KEGG:R09389 | GO:(S)-limonene 1,2-monooxygenase activity | GO:0052601 |
| KEGG:R09393 | GO:(R)-limonene 1,2-monooxygenase activity | GO:0018635 |
| KEGG:R09494 | GO:formate dehydrogenase (quinone) activity | GO:0036397 |
| KEGG:R09532 | GO:bisphosphoglycerate 3-phosphatase activity | GO:0034417 |
| KEGG:R09533 | GO:baicalin beta-D-glucuronidase activity | GO:0052748 |
| KEGG:R09541 | GO:ferric-chelate reductase (NADPH) activity | GO:0052851 |
| KEGG:R09550 | GO:glucose-6-phosphate dehydrogenase (coenzyme F420) activity | GO:0052749 |
| KEGG:R09565 | GO:gallate dioxygenase activity | GO:0036238 |
| KEGG:R09651 | GO:N-acetylglucosaminylinositol deacetylase activity | GO:0035595 |
| KEGG:R09656 | GO:9,9'-dicis-carotene:quinone oxidoreductase activity | GO:0052886 |
| KEGG:R09658 | GO:7,9,9'-tricis-neurosporene:quinone oxidoreductase activity | GO:0052887 |
| KEGG:R09662 | GO:flavin reductase (NADH) activity | GO:0036382 |
| KEGG:R09704 | GO:hydantoin racemase activity | GO:0036348 |
| KEGG:R09750 | GO:riboflavin reductase (NADH) activity | GO:0052875 |
| KEGG:R09819 | GO:3-hydroxy-9,10-secoandrosta-1,3,5(10)-triene-9,17-dione monooxygenase activity | GO:0036383 |
| KEGG:R09838 | GO:phenylacetyl-CoA 1,2-epoxidase activity | GO:0097266 |
| KEGG:R09859 | GO:cholest-4-en-3-one 26-monooxygenase activity | GO:0036199 |
| KEGG:R09860 | GO:3-ketosteroid 9-alpha-monooxygenase activity | GO:0036200 |
| KEGG:R09861 | GO:ent-isokaurene C2-hydroxylase activity | GO:0036201 |
| KEGG:R09865 | GO:9beta-pimara-7,15-diene oxidase activity | GO:0036209 |
| KEGG:R09866 | GO:ent-cassa-12,15-diene 11-hydroxylase activity | GO:0036202 |
| KEGG:R09867 | GO:taxoid 14-beta-hydroxylase activity | GO:0036203 |
| KEGG:R09868 | GO:taxoid 7beta-hydroxylase activity | GO:0036239 |
| KEGG:R10089 | GO:pyridoxal 5'-phosphate synthase (glutamine hydrolysing) activity | GO:0036381 |
| KEGG:ko00730 | GO:1-deoxy-D-xylulose 5-phosphate metabolic process | GO:0052863 |
| KEGG:ko00900 | GO:1-deoxy-D-xylulose 5-phosphate metabolic process | GO:0052863 |
| KEGG:ko01100 | GO:1-deoxy-D-xylulose 5-phosphate metabolic process | GO:0052863 |
| KEGG:ko01110 | GO:1-deoxy-D-xylulose 5-phosphate metabolic process | GO:0052863 |
| KEGG:map01060 | GO:1-deoxy-D-xylulose 5-phosphate metabolic process | GO:0052863 |
| KEGG:map01062 | GO:1-deoxy-D-xylulose 5-phosphate metabolic process | GO:0052863 |
| KEGG:map01066 | GO:1-deoxy-D-xylulose 5-phosphate metabolic process | GO:0052863 |
| KEGG:map01070 | GO:1-deoxy-D-xylulose 5-phosphate metabolic process | GO:0052863 |
